# Engineering Oncolytic Coxsackievirus A21 with Small Transgenes and Enabling Cell-Mediated Virus Delivery by Integrating Viral cDNA into the Genome

**DOI:** 10.1128/jvi.00309-23

**Published:** 2023-04-18

**Authors:** Miranda Sam, Mohammed Selman, Weilong Zhao, Jiwon Jung, Aarron Willingham, Uyen Phan, Gary C. Starling, Qinshan Gao

**Affiliations:** a Discovery Biologics, Merck & Co., Inc., South San Francisco, California, USA; b Discovery Oncology, Merck & Co., Inc., South San Francisco, California, USA; c Scientific Informatics, Merck & Co., Inc., West Point, Pennsylvania, USA; Emory University School of Medicine

**Keywords:** coxsackievirus A21 (CVA21), cell therapy, gene delivery, oncolytic virus, reverse genetics, transgene

## Abstract

Coxsackievirus A21 (CVA21) is a naturally occurring RNA virus that, in preclinical studies and clinical trials, has demonstrated promising potential in treating a range of malignancies. Other oncolytic viruses, such as adenovirus, vesicular stomatitis virus, herpesvirus, and vaccinia virus, all can be engineered to carry one or more transgenes for various purposes, including immune modulation, virus attenuation, and induction of apoptosis of tumor cells. However, it remained unknown whether CVA21 can express therapeutic or immunomodulatory payloads due to its small size and high mutation rate. Using reverse genetics techniques, we demonstrated that a transgene encoding a truncated green fluorescent protein (GFP) of up to 141 amino acids (aa) can be successfully carried in the 5′ end of the coding region. Furthermore, a chimeric virus carrying an eel fluorescent protein, UnaG (139 aa), was also made and shown to be stable, and it maintained efficient tumor cell-killing activity. Similar to other oncolytic viruses, the likelihood of delivering CVA21 by the intravenous route is low due to issues like blood absorption, neutralizing antibodies, and liver clearance. To address this problem, we designed the CVA21 cDNA under the control of a weak RNA polymerase II promoter, and subsequently, a stable cell pool in 293T cells was made by integrating the resulting CVA21 cDNA into the cell genome. We showed that the cells are viable and able to persistently generate rCVA21 *de novo*. The carrier cell approach described here may pave the way to designing new cell therapy strategies by arming with oncolytic viruses.

**IMPORTANCE** As a naturally occurring virus, coxsackievirus A21 is a promising oncolytic virotherapy modality. In this study, we first used reverse genetics to determine whether A21 can stably carry transgenes and found that it could express up to 141 amino acids of foreign GFP. The chimeric virus carrying another fluorescent eel protein UnaG (139 amino acids) gene also appeared to be stable over at least 7 passages. Our results provided guidance on how to select and engineer therapeutic payloads for future A21 anticancer research. Second, the challenges of delivering oncolytic viruses by the intravenous route hamper the broader use of oncolytic viruses in the clinic. Here, we used A21 to show that cells could be engineered to stably carry and persistently release the virus by harboring the viral cDNA in the genome. The approach we presented here may pave a new way for oncolytic virus administration using cells as carriers.

## INTRODUCTION

Oncolytic viruses (OVs) belong to a new class of cancer immunotherapy that employs live viruses to specifically infect and destroy tumors in patients (1, 2). These viruses utilize two mechanisms of action against cancer. The first is direct killing and lysis of tumor cells. In contrast to normal cells, most cancer cell types have lost their ability to mount an effective antiviral response when infected by viruses. Oncolytic viruses utilize this unique feature to infect, replicate, and destroy cancer cells while sparing normal cells (3). The second mechanism is the induction of systemic antitumor immunity as a result of the direct cancer cell killing, which leads to tumor clearance through the action of tumor-specific immune cells (4). Many trials are currently ongoing in the clinic that use oncolytic viruses for cancer therapy, and there have been several approvals by different government regulatory agencies around the world. In the early 2000s, two adenovirus (Ad)-based therapies were approved for treatment of head and neck cancer when used in combination with chemotherapy. They include a replication-incompetent Ad-p53, which lacks the E1 region and expresses a wild-type p53 gene, and a replication-competent H101, which is a type 5 Ad defective of the E1B 55-kDa molecule for selective propagation in cancer cells (5). Notably, in 2015, an engineered herpesvirus-based virotherapy, T-Vec (IMLYGIC), was approved for the treatment of unresectable melanoma as a monotherapy (6). Finally, G47Δ (Delytact), another herpesvirus-based therapy, was granted conditional approval in 2021 as the first virotherapy to treat malignant glioblastoma (7).

Both DNA and RNA viruses have been explored for cancer therapy. DNA viruses include adenovirus, vaccinia virus, herpesvirus, and parvovirus; and RNA viruses are exemplified by reovirus, coxsackievirus, poliovirus, measles virus, Newcastle disease virus, and vesicular stomatitis virus (1, 2). Among these diverse types of viruses, most are genetically engineered to either attenuate their pathogenicity for safety or carry transgenes expressing various immune modulators to augment antitumor immunogenicity (8). In contrast, there are only a few naturally occurring viruses being used for oncolytic virotherapy, including coxsackievirus A21 (CVA21), a “common cold” enterovirus.

CVA21 infects cells through intercellular adhesion molecule-1 (ICAM-1) and decay-accelerating factor (DAF) receptors (9, 10). It has a proven record of safety and efficacy through multiple phase 1 and 2 clinical trials against various solid tumors (11–14). However, the very nature of using a naturally occurring virus as an anticancer modality has fundamental limitations due to the lack of benefits added by transgenes. Prior studies have demonstrated feasibility of inserting small microRNA (miRNA) target sequences into the 5′ untranslated region (5′ UTR) and 3′ UTR (15–17). However, maximum transgene size and other tolerable insert locations are unknown. In this study, we investigated the engineering of foreign genes into the genome of CVA21 and explored the maximum insert length considering the small and rigid nature of CVA21 virus particles (18). We used the random transposon screening method to scan the entire CVA21 genome and found that the region upstream of the first gene, VP4, has more flexibility to accommodate a foreign insert. We also used a truncated green fluorescent protein (GFP) gene to show that the CVA21 virus can express a foreign protein of up to 141 amino acids; likewise, we successfully made a stable chimeric CVA21 expressing a small fluorescent protein, UnaG (19, 20), of 139 amino acids, which maintained robust tumor cell killing *in vitro*.

In addition, the intratumoral route of drug delivery remains the dominant method for most virotherapies in the clinic. Ideally, systemic delivery, such as intravenous (i.v.) administration, is the most desirable delivery method due to its potential to reach both primary and metastasized tumors. From a practical point of view, it is also more convenient and easier to administer the drug in a regular medical setup. However, there are enormous challenges facing the i.v. option. Issues like blood and liver binding and clearance, preexisting antibody neutralization, cytokine storm risks, and inefficient targeting of tumor sites remain to be addressed (21). In recent years, novel cancer therapies such as chimeric antigen receptor (CAR)-T-cell and CAR-NK have revolutionized the oncology field (22). The concept of using cells to express and then deliver the protein- or RNA-based therapeutic has begun to emerge. In this report, we initiated a study to determine whether cells can be used as carriers to deliver oncolytic virus. We demonstrated that the cDNA of CVA21 can be successfully integrated into the cell genome when placed under a weak promoter; the cells are able to stably carry the virus genes over multiple passages and release live CVA21 virus into the media. This cell-mediated and genome-integrated strategy may help to solve the issues associated with systemic i.v. delivery of OV for cancer treatment.

## RESULTS

### Random transposon insertion mutagenesis of CVA21 cDNA indicated that the 5′ UTR region adjacent to the open reading frame (ORF) is more amenable to changes and insertions.

**FIG 1:**
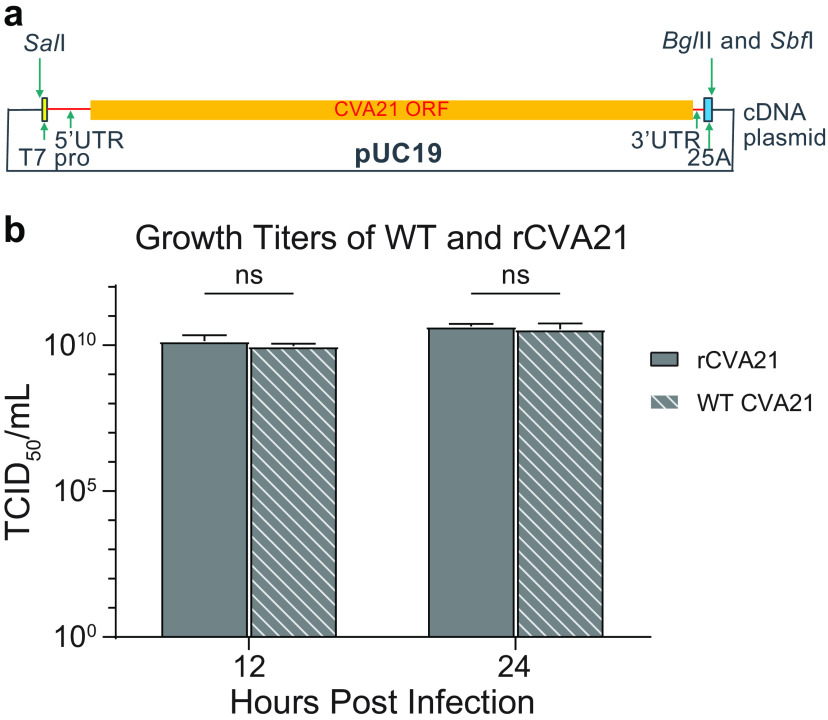
Reverse genetics system for CVA21. (a) Schematic of reverse genetics system of CVA21 cDNA plasmid to yield rCVA21. The CVA21 cDNA is flanked by a T7 promoter at the 5′ end and a 25A sequence at the 3′ end. The plasmid was linearized and transcribed *in vitro* to generate vRNA. rCVA21 was generated following transfection of SK-Mel-28 cells with transcribed vRNA. (b) Growth titers of original WT CVA21 versus rCVA21 at 12 and 24 h after infection of SK-Mel-28 cells at an MOI of 1. The experiments were performed in triplicate, and titers were determined using the TCID_50_ method (mean with SD; *n* = 3). Error bars represent standard deviations. ns, *P* ≥ 0.05, not significant.

Here, a reverse genetics system was established to generate recombinant CVA21 from cDNA plasmid (Fig. 1a). CVA21 cDNA was inserted into pUC19 plasmid backbone with a T7 promoter and a 25-base-long poly(A) tail. Linearized DNA was transcribed into infectious RNA. Transfection of SK-Mel-28 cells with CVA21 RNA resulted in the generation of recombinant CVA21 (rCVA21), and its growth rate is indistinguishable from that of the original wild-type (WT) CVA21 by measuring titers at 12 and 24 h after infection of SK-Mel-28 cells at a multiplicity of infection (MOI) of 1 (Fig. 1b).

**FIG 2:**
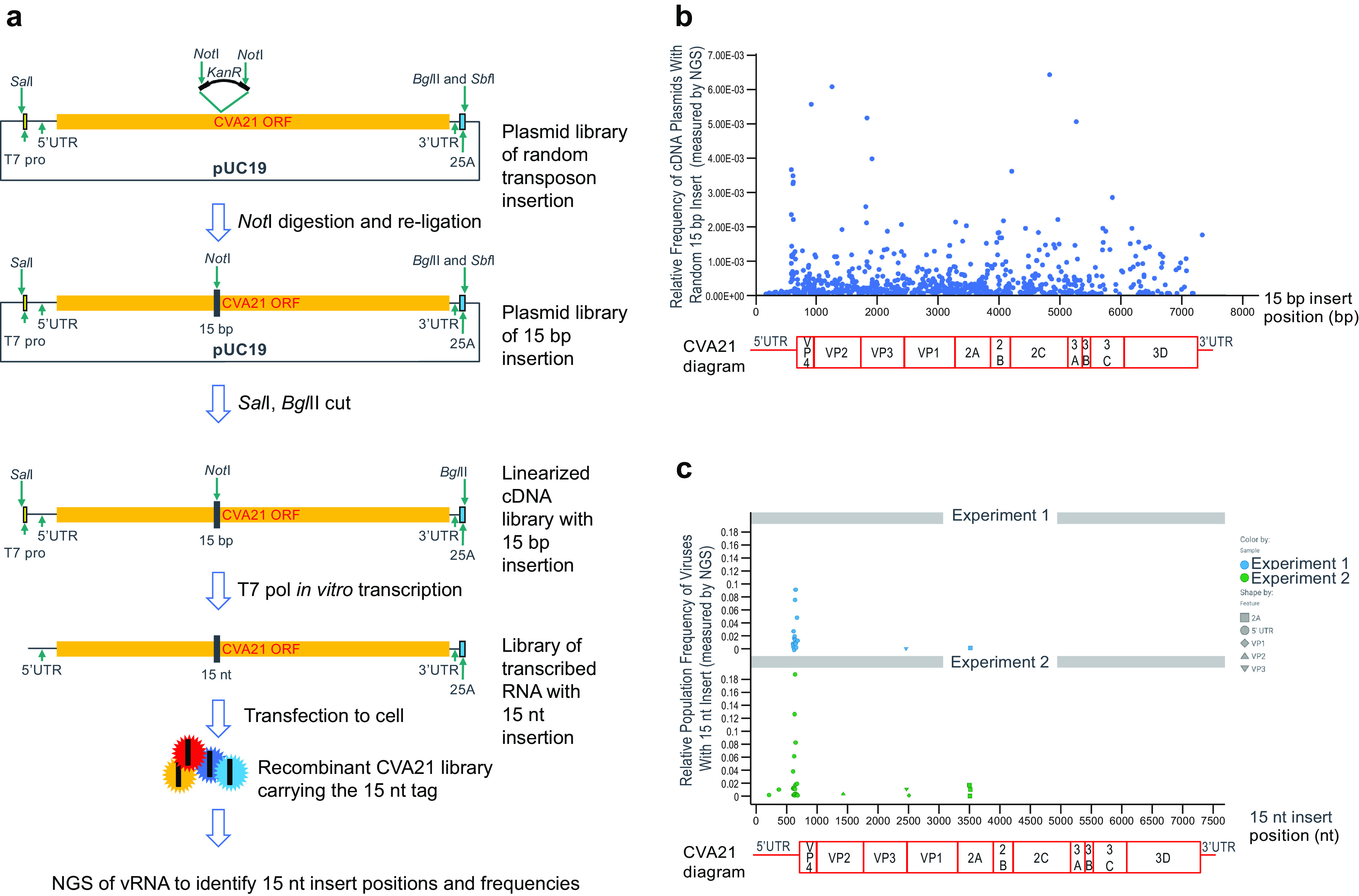
Random transposon insertion mutagenesis system identifies potential transgene locations in the CVA21 genome. (a) Schematic of Mutation Generation System (Thermo Scientific) applied to CVA21 cDNA followed by recombinant CVA21 virus library generation. The vRNA was purified from the resulting virus library and sent for standard RNA next-generation sequencing (NGS). (b) Relative frequency of cDNA plasmids with 15-bp insert in plasmid library measured by NGS indicates approximately random, equal insertion across CVA21 cDNA. Only a random small portion of the insertions (5%) was plotted in the graph here due to limited space. Right below the *x* axis, the CVA21 genome diagram was drawn to scale to show the positions of viral genes. (c) Relative frequency of rCVA21 with 15-nt insert in rescued virus library measured by NGS indicates tolerable insert locations identified using the procedures listed in panel a. Two independent experiments were performed, and the insertion positions and frequencies are shown for each experiment.

Transgenes encoding small proteins of various sizes have been inserted into the genomes of small RNA viruses like poliovirus (23–25), enterovirus A71 (26), coxsackievirus B3 (CVB3) (27–29), foot-and-mouth disease virus (FMDV) (30), hepatitis A virus (31), and Seneca Valley virus (32), to name a few. It is known that small miRNA target sequences can be engineered into the 5′ UTR and 3′ UTR of CVA21 (15–17). To our knowledge, however, whether CVA21 can stably carry a foreign protein- or peptide-expressing gene in its genome is still unknown. Questions remain regarding which location is most ideal for insertion with minimal impact on CVA21 replication and fitness and to what degree it can tolerate the insertion in terms of length due to the nature of its small and rigid capsid particle. To identify the optimal insertion locations, we performed random transposon insertion mutagenesis to randomly insert a 15-bp sequence into the CVA21 cDNA to create a plasmid library, which was then transcribed *in vitro* to make an RNA library randomly carrying the 15-nucleotide (nt) insert (Fig. 2a). Cells were transfected with the RNA library to generate a recombinant virus library. The virus particles in the library would harbor the 15 nt at genome positions that the virus was able to tolerate (Fig. 2a). Viral RNA (vRNA) purified from the virus library was sequenced by standard RNA next-generation sequencing (NGS), RNA-seq, followed by bioinformatics to selectively focus on sequences carrying the 15-nt insertion. For control comparison, the plasmid library with 15 bp inserted was also sequenced by NGS to ensure equal, random distribution of the inserts throughout the CVA21 cDNA. Indeed, this was largely the case (Fig. 2b). Sequencing of the vRNA recovered from the virus library showed a relatively high frequency of multiple isolates with insertions around nt 610 to 682 positions near the end of 5′ UTR, indicating that changes and insertions in this area are well tolerated (Fig. 2c). Our finding also agrees with a previous report that miRNA target sequences can be engineered into this location with minimal impact on CVA21 infectivity (15). A few additional locations that could tolerate the 15-nt insert were also identified, including two in the middle of 5′ UTR, one in VP2, three in the VP3-VP1 junction, and three in 2A (Fig. 2c). However, relatively lower frequencies were observed for these locations than insertions near the end of 5′ UTR (Fig. 2c), indicating that virus fitness was more impacted by these mutations.

### miRNA21 sponge sequences can be carried at the 5′ UTR.

**FIG 3:**
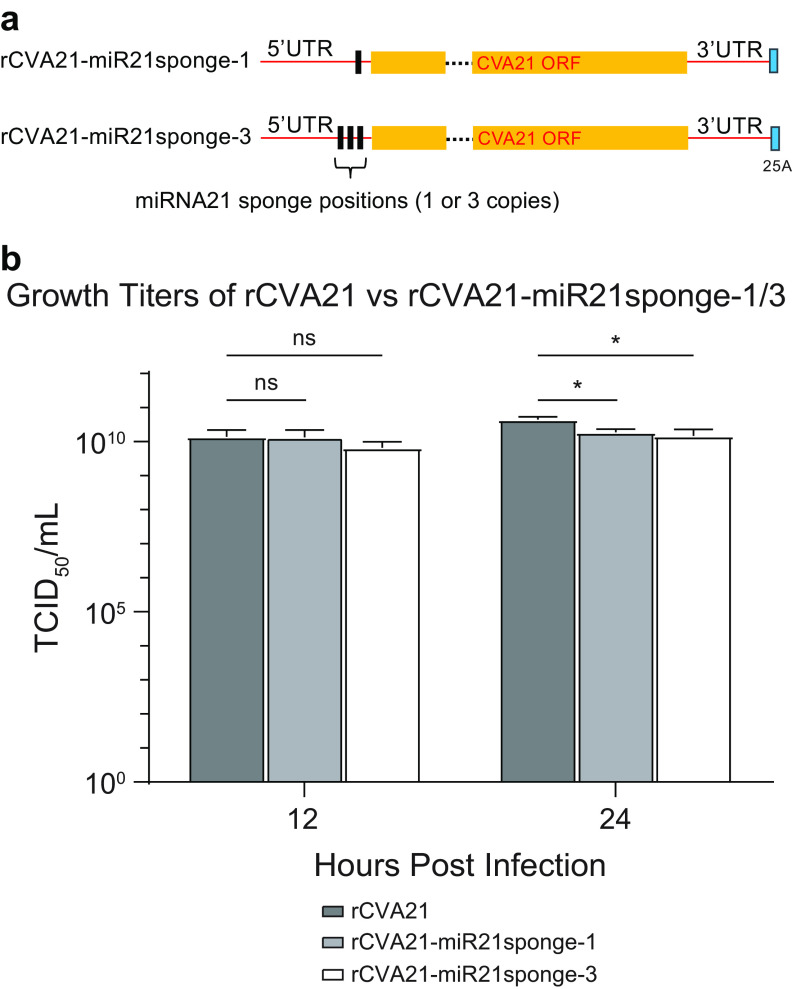
The 5′ UTR region next to the ORF region of the CVA21 genome is amenable to engineering to carry small miRNA sponge sequences. (a) Schematic showing insertion of 1 or 3 copies of miRNA21 (miR21) sponge sequences between nucleotides 641 and 698. (b) Growth titers of rCVA21 versus two rCVA21s carrying miRNA21 (miR21) sponge sequences at 12 and 24 h after infection of SK-Mel-28 cells at an MOI of 1. The experiments were performed in triplicate, and titers were determined using the TCID_50_ method (mean with SD; *n* = 3). Error bars represent standard deviations. ns, *P* ≥ 0.05, not significant; *, 0.01 < *P* < 0.05, significant.

Based on the above random transposon screening results and considering previously published reports on the engineering of miRNA target sequence into both 5′ and 3′ UTRs of CVA21 (15, 17), here, we targeted nt 641 to 698 on the 5′ UTR for making changes and insertions. As proof of concept, we engineered one or three copies of miRNA21 sponge sequences into the nt 641 to 698 region of CVA21 cDNA (Fig. 3a). MicroRNA sponges are decoy miRNA target sequences that derepress the real target sites by binding and absorbing miRNAs (33, 34). Using the reverse genetics system described above, SK-Mel-28 cells were transfected with the modified sponge-carrying CVA21 RNAs, and two recombinant viruses (rCVA21-miR21sponge-1 and rCVA21-miR21sponge-3) were generated (Fig. 3a). We compared the growth of these two recombinant viruses versus rCVA21 at 12 and 24 h after infection of SK-Mel-28 cells at an MOI of 1. The difference was unnoticeable at 12 h. At 24 h, the titers of the two viruses with sponge sequences were slightly lower than rCVA21. However, the difference was less than 10-fold, and all viruses could reach more than 10^10^ 50% tissue culture infective dose (TCID_50_) in titer (Fig. 3b). Sanger sequencing of the reverse transcriptase PCR (RT-PCR) fragments of both vRNAs around this region confirmed the designed sponges were intact (data not shown). These results indicated that there is indeed flexibility in using a modified 5′ UTR to carry potentially functional RNA sequences, and changes in the nt 641 to 698 region apparently have a minimal impact on the virus growth titer.

### Transgenes encoding proteins up to 141 amino acids can be engineered into the 5′ UTR-VP4 junction.

**FIG 4:**
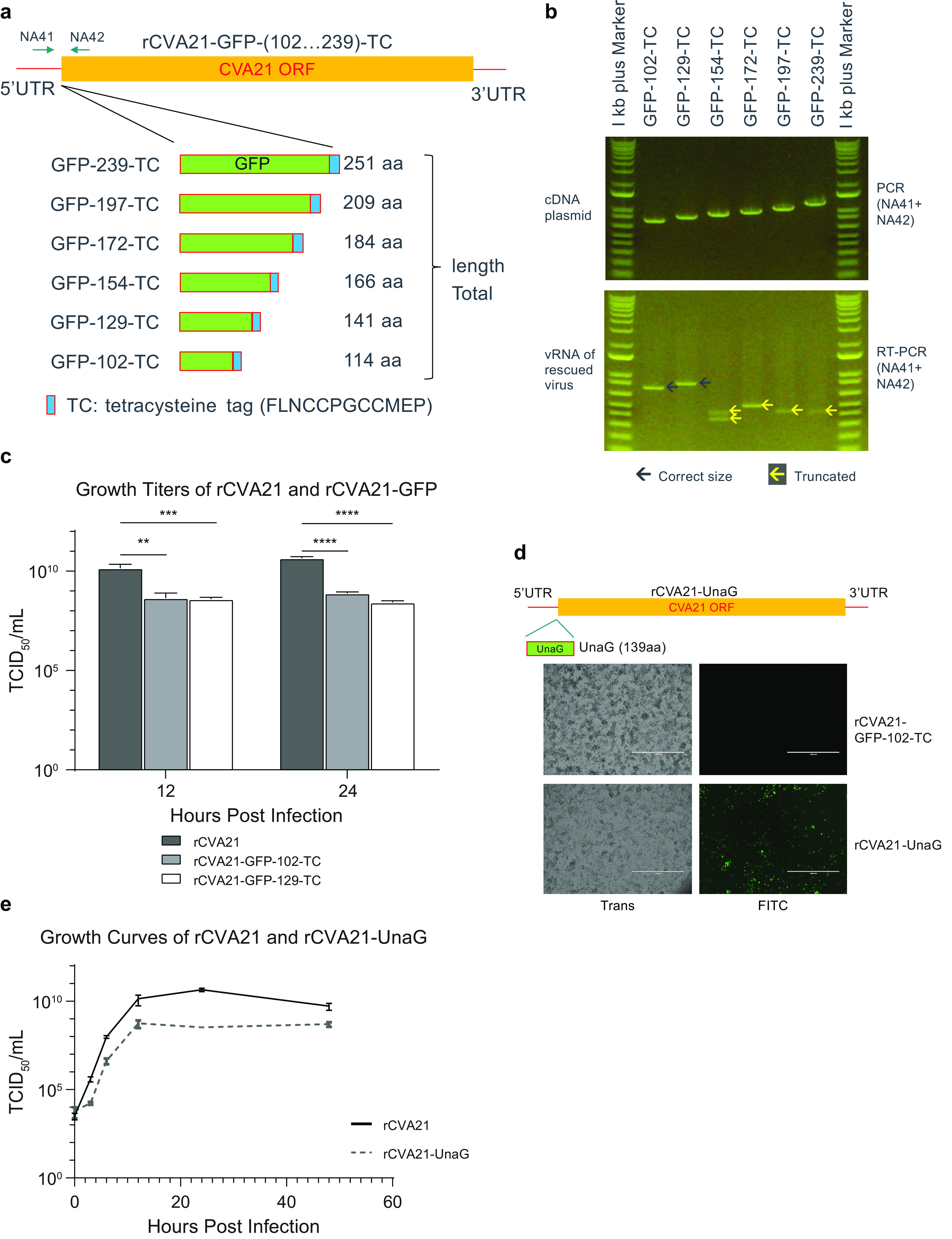
Tolerability of up to 141 amino acids at the 5′ UTR-VP4 junction. (a) Schematic showing insertion of various-sized GFP inserts placed upstream of the VP4 gene. Each truncated (102- to 197-aa) or full-length GFP (239-aa) ORF followed by a tetracysteine (TC) tag sequence was inserted and connected to the VP4 gene through an in-frame 3C cleavage site. (b) RT-PCR results showed the stability of GFP and TC inserts encoding up to 141 aa in rescued recombinant viruses. The primer pair NA41 and NA42, whose positions are shown in panel a, is used for PCR to amplify the region spanning the 5′ UTR-VP4 junction. The top gel shows the PCR from input cDNA plasmids; the bottom is the RT-PCR result from purified vRNAs derived from rescued viruses. The solid arrows designate intact inserts, and hollow ones are shortened inserts compared with cDNA inputs. (c) Growth titers of rCVA21 versus two rCVA21s carrying GFP sequences at 12 and 24 h after infection of SK-Mel-28 cells at an MOI of 1. The experiments were performed in triplicate, and titers were determined using the TCID_50_ method (mean with SD; *n* = 3). Error bars represent standard deviations. **, 0.001 < *P* < 0.01; ***, 0.0001 < *P* < 0.001; ****, *P* < 0.0001. (d) rCVA21-UnaG recombinant virus generation and testing of fluorescent protein expression in SK-Mel-28 cells post-viral infection. The UnaG ORF encoding 139 aa was inserted into the CVA21 cDNA in the same way as GFP truncation inserts in panel a. The resulting rCVA21-UnaG virus was rescued and passaged 7 times in SK-Mel-28 cells under an MOI of 0.001 conditions. At passage 5, the virus was used to infect SK-Mel-28 at an MOI of 0.5. Transmitted light (trans) and fluorescent pictures were taken 12 h postinfection prior to the appearance of severe CPE. The rCVA21-GFP-102-TC virus was also analyzed here for negative control under the same condition. (e) One-step growth curve analysis of rCVA21 versus rCVA21-UnaG in SK-Mel-28 cells. SK-Mel-28 cells were infected at an MOI of 1, and samples were harvested at each time point (0, 3, 6, 12, 24, and 48 h). The experiments were performed in triplicate, and titers were determined using the TCID_50_ method (mean with SD; *n* = 3). Error bars represent standard deviations.

To further explore the maximum transgene length that could be carried by CVA21, transgenes of various-sized GFP ranging from truncated to full-length versions (102 to 239 amino acids [aa]), each tagged with a tetracysteine (TC) sequence, were inserted into the cDNA by fusing in-frame to the downstream VP4 gene using a 3C protease site (Fig. 4a). The 5′ UTR-VP4 junction was chosen here based on the transposon screening results shown above and previously reported findings on other similar viruses (23, 24, 26, 28, 29). These recombinant viruses were generated in SK-Mel-28 cells using the same procedure described above. After one passage under low-MOI conditions, the vRNAs were purified, and the 5′ UTR-VP4 junction was subsequently amplified by RT-PCR to determine whether the inserts were still maintained intact in the genome (Fig. 4b, bottom picture). For control, the same region in cDNA plasmids was also amplified by PCR using the same pair of primers (Fig. 4b, top picture). By comparing the lengths of amplified fragments from plasmids and vRNAs, we determined that only two of the rescued viruses, GFP-102-TC and GFP-129-TC, expressing a partial GFP of 114 and 141 amino acids, respectively, still retained the intact inserts in the genome; in contrast, for GFP-154-TC and the remaining larger constructs, the inserts were truncated: the sizes of amplified fragments from vRNAs were much smaller than those from corresponding plasmids (Fig. 4b). Sanger sequencing of RT-PCR fragments of vRNAs from all rescued viruses (shown in Fig. 4a, passage 1) around this region also confirmed the inserts of GFP-102-TC and GFP-129-TC were intact, while the rest were truncated (data not shown). We compared the growth of the two recombinant viruses, GFP-102-TC and GFP-129-TC, versus rCVA21 at 12 and 24 h after infection of SK-Mel-28 cells at an MOI of 1. There was about 2-log reduction in titers for the viruses with partial GFP insertions, indicating the impact of insertions of transgenes of such lengths on virus growth (Fig. 4c). Furthermore, to see if a transgene inserted at this region is indeed expressed and functional, a small gene encoding an eel fluorescent protein of 139 amino acids, UnaG (19), was inserted, and recombinant virus was generated (Fig. 4d). We grew and passaged the rCVA21-UnaG virus up to 7 times under an MOI of around 0.001. When SK-Mel-28 cells were infected with rescued rCVA21-UnaG, for all 7 passages, a vivid green color was seen under a fluorescence microscope approximately 12 h postinfection; in contrast, we did not see any green signals under the same conditions for the recombinant virus carrying GFP-102-TC due to the lack of a complete GFP chromophore (Fig. 4d). This indicates that the UnaG protein was indeed expressed, and its fluorescent domain was functional. The insert was also confirmed to be intact by Sanger sequencing of the RT-PCR fragment from vRNA (data not shown). We performed a complete one-step growth curve analysis of rCVA21-UnaG versus rCVA21 after infection of SK-Mel-28 cells at an MOI of 1. The impact of UnaG insertion on the growth was clearly noticeable, with about a 2-log reduction in maximal titers (Fig. 4e), similar to what we observed for the viruses carrying partial GFP genes (Fig. 4c).

### Chimeric CVA21 carrying the UnaG transgene maintains efficient cancer cell killing.

**FIG 5:**
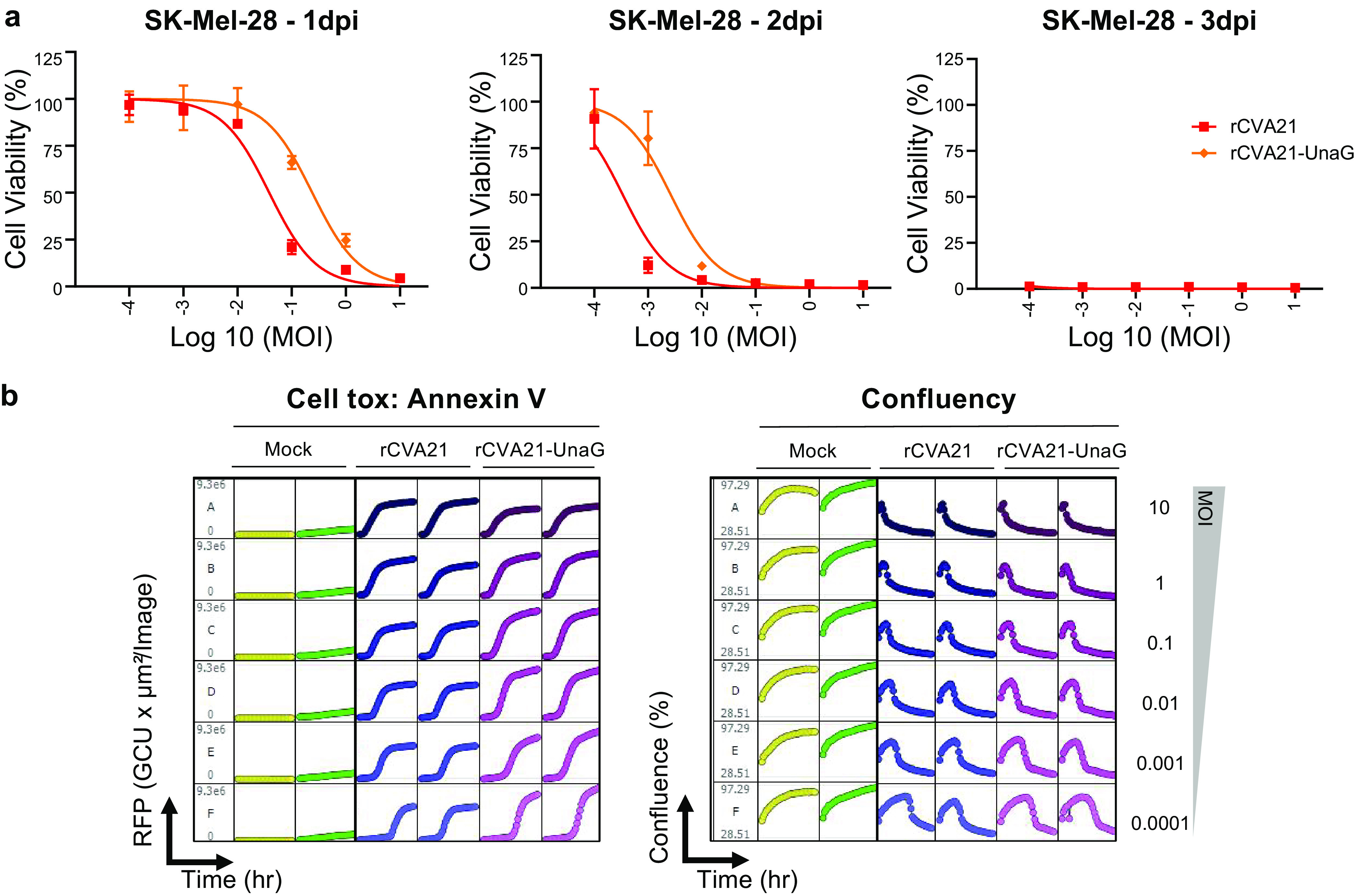
Tumor cell-killing activity of rCVA21-UnaG compared to rCVA21. (a) Cell viability of SK-Mel-28 cells postinfection with rCVA21 or rCVA21-UnaG at an MOI range of 10 to 0.0001 as assessed using CellTiter-Glo 2.0 assay in 96 wells (mean with SD; *n* = 3). For a continuous 3 days (1 to 3 days postinfection [dpi]), the infected cells were subjected to the assay. Data were normalized to uninfected conditions with blank subtracted. (b) Comparison of the apoptosis caused by rCVA21 versus rCVA21-UnaG using annexin V staining monitored by IncuCyte. Cells were seeded in 96-well assay plates, and the viruses and annexin V reagents were added the following day under MOIs of 10 to 0.0001. The data were taken at 2-h intervals for 3 days. (b, Left) Degree of apoptosis measured by red fluorescent protein (RFP) channel in a time-lapsed manner. (b, Right) Cell confluence of SK-Mel-28 cells monitored by IncuCyte. Duplicate experiments are shown here.

To examine whether the additional UnaG transgene impacted the tumor cell-killing capability of the recombinant virus, rCVA21-UnaG was assessed in two *in vitro* cell toxicity assays on SK-Mel-28 cells compared to rCVA21. Cell viability of SK-Mel-28 cells postinfection with rCVA21 or rCVA21-UnaG was compared using CellTiter-Glo 2.0 assay (Fig. 5a) and annexin V dye staining using IncuCyte (Fig. 5b). These assays showed rCVA21-UnaG maintained efficient cell killing (Fig. 5a) and induced apoptosis (Fig. 5b); however, the effects were slightly delayed in both cases compared with rCVA21, indicating that the addition of a 139- aa protein-expressing transgene does cause some degree of virus attenuation.

### When driven by a weak promoter, CVA21 cDNA can be integrated into the cellular genome to enable persistent *de novo* generation of live virus from growing cells.

**FIG 6:**
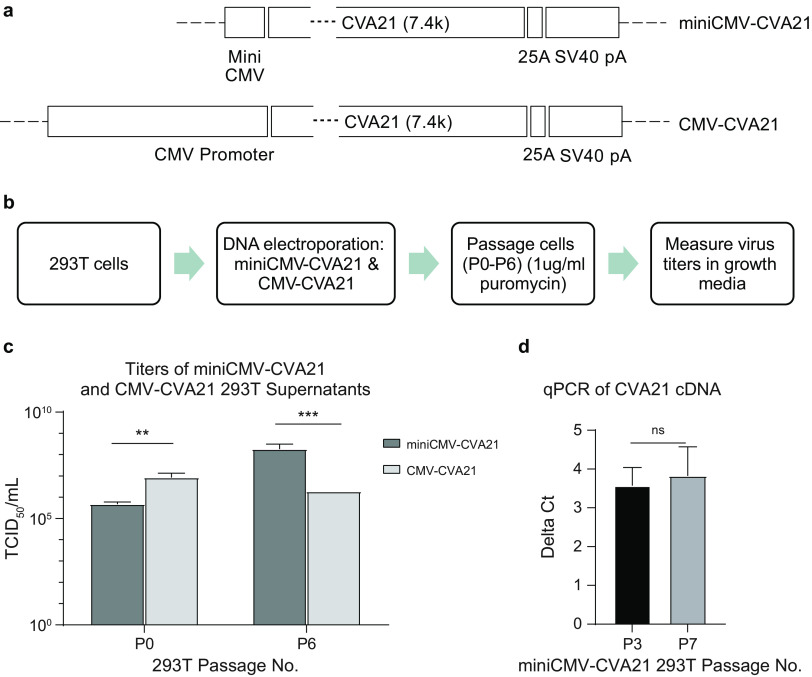
miniCMV promoter-driven CVA21 cDNA integration into the cell genome and subsequent *de novo* generation of recombinant virus from stable cell pool. (a) Schematic of design of CVA21 cDNA cassettes driven by either a miniCMV or normal CMV promoter. A 25A stretch and SV40 poly(A) sequence were placed at the 3′ ends. (b) Diagram of experimental procedures used to generate stable cell pools by integrating miniCMV-CVA21 or CMV-CVA21 into the cell genome followed by cell passaging and virus titer determination in growth media. (c) Titer of supernatants from 293T cells electroporated (P0) and passaged 6 times (P6) in the presence of puromycin selection. The P0 samples were harvested from cells 1 day postelectroporation in triplicate wells for each plasmid. The P6 samples were harvested from triplicate wells on day 3 of passage 6 for each group. Error bars represent standard deviations. **, 0.001 < *P* < 0.01; ***, 0.0001 < *P* < 0.001. (d) qPCR quantification of CVA21 cDNA levels at passages 3 (P3) and 7 (P7) of miniCMV-CVA21 293T cell pool. The experiment was done in 12 repeats for each sample. Error bars represent standard deviations. ns, *P* ≥ 0.05, not significant.

Next, we investigated whether CVA21, a lytic positive-strand RNA virus with no history of host genome integration or latency, can be persistently generated from cells without causing their death. For this purpose, we chose 293T cells, which are negative in CVA21 primary receptor ICAM-1 expression (35). Lack of ICAM-1 ensures there would be no productive second-round virus replication within the carrier cells, while they still can continuously produce live CVA21 virus and release it into the media. Instead of the T7 promoter used in the above-described experiments, here, the CVA21 cDNA is driven by either a weak minimal cytomegalovirus (miniCMV) promoter or strong full-length CMV, which are both recognized by RNA polymerase II. The simian virus 40 (SV40) poly(A) signal was also placed at the end of the 25A stretch to provide efficient transcription termination (Fig. 6a). 293T cells were electroporated with miniCMV-CVA21 or CMV-CVA21 plasmid and passaged 6 times in the presence of puromycin selection (P0 to P6). Then, titers of the supernatants from P0 and P6 were determined in SK-Mel-28 cells using the TCID_50_ method (Fig. 6b). One day after electroporation of 293T cells, both miniCMV-CVA21 and CMV-CVA21 plasmids produced live rCVA21 virus. However, the virus titer in the P0 supernatant of CMV-CVA21 plasmid was more than 10-fold higher than that of miniCMV-CVA21 (Fig. 6c). At this stage, the recombinant viruses were mainly generated from plasmid DNA. The higher titer from the CMV-CVA21 plasmid was evidence that the stronger CMV promoter gave a more robust expression of CVA21 genes, therefore generating more virus. One day postelectroporation, we added puromycin to selectively enrich cells that carry the genome-integrated CVA21 cDNA. The cells were further passaged 6 times under the same condition. At the sixth passage, the supernatants (P6) harvested from day 3 were again analyzed to measure the rCVA21 virus titer. Surprisingly, the titer from miniCMV-CVA21 was about 2 logs higher than that of CMV-CVA21 at P6, even though the latter one carries a much stronger promoter (Fig. 6c). We repeated the experiment twice with slightly different versions of miniCMV promoter sequences, and the results were always the same (results not shown). The cells carrying miniCMV-CVA21 have the same morphology as the CMV-CVA21 ones, with slightly lower growth rates based on passage history. We further passaged the 293T cells carrying the miniCMV-CVA21 up to passage 10; we found that the morphology, growth rate, and virus generation ability were unchanged (results not shown). Although introducing 293T cells with plasmids and growing them in the presence of puromycin is a common laboratory practice to make stable cell pools by randomly integrating the plasmid DNAs into the cell genome, here, we did confirm this was the case. We purified cellular genomic DNA from both passages 3 and 7 of the miniCMV-CVA21 293T cell pool and then used quantitative PCR to measure the CVA21 cDNA level in reference to 18S rRNA coding DNA. The results showed that the signals of CVA21 cDNA were stably maintained between passages, and there was no reduction in the amount of cDNA detected by qPCR (Fig. 6d), indicating that the CVA21 cDNA was genome integrated, as the plasmids used here do not possess any elements that could enable their self-replication within mammalian cells. These results suggested that, indeed, the weak promoter-driven CVA21 cDNA can be integrated into the cellular genome, and the cells were able to survive and persistently produce live virus.

## DISCUSSION

In this study, we used reverse genetics to explore the possibility of engineering CVA21 as a vector to carry transgenes. Naturally occurring CVA21 has been used in clinical trials for treating cancers (11–14). Having the capability of carrying foreign transgenes to express therapeutic proteins or RNAs in tumors may further modulate anticancer immunity of the host and boost the tumor-killing potential of CVA21, as demonstrated by other oncolytic viruses (8). For CVA21, we were only able to find a couple of reports in which miRNA target sequences were engineered into the 5′- or 3′-UTR position of the virus genome to increase its safety profile as a cancer therapy modality: the resulting recombinant virus had an altered tissue tropism and possessed a lesser potential to cause myositis in the skeletal muscle in mice (15, 17). It has been shown that other similar RNA viruses, such as poliovirus (23–25) and coxsackievirus B3 (CVB3) (27–29), were able to carry a transgene expressing a peptide or protein with sizes ranging from a few amino acids to a full-length GFP. Whether CVA21 can be engineered to express the foreign proteins or peptides and what is the size of insertion are still unknown. We addressed these open questions in the current study.

First, we sought to identify which positions within CVA21 are ideal for transgene insertions. We performed random transposon mutagenesis of the entire cDNA and identified several potential tolerable insert locations in the CVA21 genome. The transposon results showed that the stretch of sequence near the end of 5′ UTR is most amenable for changes and insertions (Fig. 2). The flexibility of this part of the sequence on other similar types of viruses has been well documented (36). Although it has been shown that the genomes of many enteroviruses encode a short 6.5- to 9.0-kDa-long upstream ORF (uORF) at this region in addition to the long polyprotein coding region (37), the likely uORF from CVA21 (Kuykendall [9]) at the same location only encodes 31 amino acids, which is too short to have a similar *in vivo* function to that suggested by the previous finding (37). Therefore, insertions at this location likely may not have an impact on CVA21 virus *in vivo*. Agreeing with the screening results, we showed that up to three copies of miRNA21 sponge sequences could be engineered into this position with minimal impact on virus fitness (Fig. 3). Considering this information and the fact that fusing a foreign gene with CVA21 ORF through a virus protease cleavage site is the most efficient in space saving, we chose the 5′ UTR-VP4 junction region as insertion site to express foreign proteins or peptides. Second, considering the rigid nature of small-sized CVA21 particles (18), we also wanted to determine whether there is a length limitation for the foreign inserts. We inserted various lengths of a GFP gene followed by a tetracysteine motif sequence into the 5′ UTR-VP4 junction and found that this position can tolerate a sequence encoding a protein up to 141 amino acids long. Longer inserts encoding 166 amino acids or more were truncated in the rescued virus genomes (Fig. 4). This suggests there is indeed a limit for the insert length, which might lie somewhere between 141 and 166 amino acids. However, the exact lengths might slightly vary if insert sequences other than the GFP gene were used, as previous findings indicated that the nucleotide compositions of virus genome sequence may impact virus fitness. In particular, the number of 5′-CG-3′ dinucleotides on RNA virus genomes was shown to play a role in virus-cell interactions and replication rates (38, 39). The results we obtained here also largely agree with a previous study on another picornavirus, FMDV. It was found that FMDV can tolerate insertion of GFP gene fragment of 303 nucleotides, and truncations were seen when insert length reached 417 (30). It should be noted that the inserts in that report were placed between VP1 and 2A genes of FMDV, instead of the 5′ UTR-VP4 junction used here. In this study, with CVA21, the 5′ UTR-VP4 junction was more tolerant in our hands than other insertion sites, including this VP1-2A position (data not shown).

To track the *in vivo* biodistribution of the CVA21 oncolytic virus in preclinical models, a fluorescent protein expressed directly from the virus might be useful. However, we were unable to insert a full-length GFP (239-aa) gene into the virus genome. We finally did successfully insert a shorter gene encoding an eel fluorescent protein, UnaG (139 aa), into the genome in this study (19) (Fig. 4). The resulting rCVA21-UnaG virus grew well and was stable over at least 7 passages. The infected cells were vivid green in color under a fluorescence microscope (Fig. 4d). We compared the *in vitro* cancer cell-killing activities of rCVA21-UnaG versus rCVA21; we showed that rCVA21-UnaG still maintains efficient lytic properties, albeit slower than parental virus (Fig. 5). This fluorescent protein-expressing virus can be used in future studies on virus replication cycle, virus-host interaction, *in vitro* cancer cell killing, and the *in vivo* biodistribution of the virus in animal cancer models.

Using carrier cells to deliver OV for antitumor therapy is an emerging idea. It was reported that NK-92 cells can be used to systemically deliver an oncolytic coxsackievirus A7 in mice and showed better efficacy than oncolytic virus alone in glioblastoma tumor xenograft model (40). A recently concluded phase 1 clinical trial using neural stem cells to deliver oncolytic adenovirus to treat malignant glioma also showed promise (41). It should be noted that in these studies, the oncolytic viruses were simply mixed with carrier cells and entered the cells through receptor-mediated virus entry, endocytosis, or phagocytosis. The virus cycle of entry, replication, and exit from cells poses great challenges for manufacturing and quality control processes due to the cytopathic effect (CPE) on cells caused by the viruses. In this study, we tested a new way to “load” the cells with oncolytic virus CVA21. Unlike herpesvirus or lentivirus, CVA21 is a lytic positive-strand RNA virus which causes neither latency nor self-genome integration after reverse transcription. Instead of infecting the cells with CVA21, here, we integrated the virus cDNA into the cell genome and showed that the cells were able to not only survive but also produce and release virus into the media in a persistent manner (Fig. 6). Placing the CVA21 cDNA under the control of a weak promoter like miniCMV is the key to the design. We hypothesized that the weak promoter led to lower virus generation, and the cells were therefore able to survive. When a strong CMV promoter is used, the high titer of virus produced within the cells might cause enough damage, leading cells to adapt by either shutting down or kicking out the CVA21 cDNA. Therefore, in contrast to the miniCMV cells, the titer of virus produced from these cells was much lower after multiple passages with puromycin selection (Fig. 6c). In addition, future experiments are needed to further optimize the system, such as changing to more physiologically relevant cell carriers (T or NK) and introduction of inducible expression systems using either natural promoters (NF-κB or nuclear factor of activated T cells [NFAT]) or artificial ones (tetracycline response element or Gal4) (42–44). Such designs may allow the cells to generate more virus by responding to changes in microenvironments or upon target engagement. Considering the recent success of therapies like CAR-T and CAR-NK (22), the concept we initiated here may contribute to the discovery of new methods of systematically delivering oncolytic virus for cancer therapy using cells as carriers.

## MATERIALS AND METHODS

### Cells and viruses.

SK-Mel-28 (ATCC) and 293T (ATCC) cells were maintained in Dulbecco's modified Eagle's medium (DMEM) with 10% fetal calf serum (Gibco). Wild-type (WT) CVA21 (Kuykendall) (9) and recombinant CVA21 (rCVA21) viruses were grown and passaged in SK-Mel-28 cells. 293T cells were used for integrating CVA21 cDNA into the cell genome.

### Construction of cDNA plasmids.

To make the plasmid carrying CVA21 cDNA with the T7 promoter in the 5′ end and a 25-nucleotide (nt) poly(A) sequence in the 3′ end, the viral RNA (vRNA) was purified from wild-type CVA21 virus by using TRIzol reagent (Invitrogen). The cDNA was prepared using Maxima H Minus first-strand cDNA synthesis kit (Fisher Scientific), and then PCR was performed to amplify the entire CVA21 cDNA using primers (sense, aaggtcgactaatacgactcactatagggttaaaacagctctggggttgttccca; anti-sense, ctcctgcaggtagatctttttttttttttttttttttttttctccgaattaaagaaaaatttacccc) and Herculase II Fusion DNA polymerase (Agilent). The 7.5-kb PCR fragment was then digested with SalI and SbfI (New England Biolabs [NEB]) and then ligated to the pUC19 plasmid (Fig. 1a).

rCVA21-miR21sponge-1 and rCVA21-miR21sponge-3 (Fig. 3a) are the two CVA21 cDNA sequences carrying 1 and 3 copies of sponge sequence against microRNA (miRNA) hsa-miR-21-5p (https://mirbase.org/; MIMAT0000076) in the 5′ untranslated region (UTR). The sponge sequence (TCAACATCAGAACATAAGCTA) was designed using the online software miRNAsong (45). We also made 6 CVA21 cDNA constructs carrying various lengths of enhanced green fluorescent protein (EGFP) gene fused to a coding sequence of a 12-amino-acid (aa) tetracysteine (TC) tag (FLNCCPGCCMEP) (46) at the 3′ end as follows: rCVA21-GFP-102-TC, rCVA21-GFP-129-TC, rCVA21-GFP-154-TC, rCVA21-GFP-172-TC, rCVA21-GFP-197-TC, and rCVA21-GFP-239-TC. The EGFP and TC sequences are fused in-frame to the CVA21 VP4 gene by a sequence coding the 3C cleavage site (ALFQGAQ). We also made a CVA21 cDNA sequence, rCVA21-UnaG (Fig. 4d) expressing a small green-colored fluorescent protein, UnaG, derived from the Japanese freshwater eel unagi (19). The UnaG gene is connected to the VP4 gene in the same way as GFP and the TC tag.

The T7 promoter-driven CVA21 cDNA plasmid was also modified to generate two RNA polymerase II-driven plasmids, miniCMV-CVA21 and CMV-CVA21 (Fig. 6a). In both plasmids, the T7 promoter from the original cDNA construct (Fig. 1a) was replaced in the 5′ end by miniCMV or CMV promoter, and an SV40 poly(A) sequence was added downstream of the 25A sequence.

All the above-mentioned CVA21 cDNA plasmid sequences are listed in Supplemental File 1. The WT CVA21 cDNA sequence used in this study is largely the same as the original reported Kuykendall GenBank sequence, accession no. AF465515, but with a few nucleotide differences, which were likely introduced during passages in cells.

### Generation of recombinant CVA21 viruses.

To generate recombinant CVA21 viruses, cDNA plasmids were linearized with BglII and SalI (NEB) and transcribed into RNA *in vitro* using mMessage mMachine T7 transcription kit (Invitrogen). RNA was then transfected into SK-Mel-28 cells using Lipofectamine 2000 (Invitrogen). Cultures were monitored daily under a microscope and harvested when CPE was visible.

### Random transposon mutagenesis of cDNA followed by rCVA21 virus library generation.

The Mutation Generation System kit (Thermo Scientific) was used to employ the transposon method to create a plasmid library with a 15-bp sequence (TGCGGCCGCANNNNN, where NNNNN represents the duplicated target site) randomly inserted into CVA21 cDNA. The plasmid library DNA was then linearized and transcribed, and RNA was transfected to generate the recombinant virus library using the same method as described above for wild-type virus.

### Virus titration by TCID_50_.

We determined the titer of CVA21 viruses on SK-Mel-28 cells. Cells were seeded in 96-well plates at 85,000 cells per well. Serial 10-fold dilutions were made for appropriate viruses in Opti-MEM (Gibco) with 0.35% bovine serum albumin (BSA; Sigma). After 4 h of seeding SK-Mel-28 cells on the plates, the cell medium was removed, and 30 μL of dilution was added to each of 6 to 12 duplicate wells. After incubating for 1 h at 37°C, 70 μL of cell medium was added to each well. After 3 days, wells were examined for cytopathic effects (CPEs). Wells with any amount of CPE were considered positive. TCID_50_ values were then calculated by Reed-Muench method.

### One-step growth analysis of CVA21 viruses.

The day before infection, SK-Mel-28 cells were seeded in 24-well plates at 5 × 10^5^ cells/well. The next day, the cell medium was removed, and CVA21 viruses were added in triplicate per time point at an MOI of 1 in 100 μL of Opti-MEM plus 0.35% BSA for 1 h at 37°C. Virus inoculum was removed, and wells were washed 3× with phosphate-buffered saline (PBS). Then, 0.5 mL fresh medium was added to each well. At each time point (0, 3, 6, 12, 24, 48 h), the plates were frozen at −80°C. Plates were then thawed, frozen, and thawed again. All samples were centrifuged at 11,000 × *g* for 15 min at 4°C. Supernatant was aliquoted and frozen at −80°C, and we determined the titer as described above.

### Next-generation sequencing and bioinformatics.

The vRNA was purified from the virus library carrying the random 15-nt sequence by using TRIzol reagent (Invitrogen) and then submitted for standard RNA-seq deep sequencing (Genewiz). The DNA plasmid library was also submitted for NGS for comparison. Bioinformatics tools were then used to selectively filter sequences containing the inserted 10-nt core sequence (TGCGGCCGC). The positions and frequencies of all insertions were then calculated and visualized in Tibco Spotfire.

### Assessment of cell toxicity by CellTiter-Glo 2.0 assay.

Cell viability of SK-Mel-28 cells was measured using CellTiter-Glo 2.0 (Promega) following kit protocol after infection with rCVA21 or rCVA21-UnaG. Briefly, the day before infection, SK-Mel-28 cells were seeded in 96-well plates at 2.5 × 10^6^ cells/plate. The next day, 10-fold serial dilutions of viral stocks were prepared in DMEM to give an MOI of 10 to 0.0001 for 100 μL of virus added per well of cells. Then, 100 μL of appropriate dilution was added to each well. Plates were incubated at 37°C with 5% CO_2_. On days 1, 2, and 3 postinfection, plates were analyzed. CellTiter reagent was thawed in a 22°C water bath and then mixed by inverting. The plate was equilibrated to room temperature for ~30 min, and then an equal volume of reagent was added to each well. The plate was mixed for 2 min on a shaker and then incubated for 10 min at room temperature. Luminescence was read on a plate reader. Data were normalized to uninfected condition and blank subtracted to give the percentage of cell viability. The experiment was performed in triplicate wells.

### Measuring apoptosis by IncuCyte annexin V reagents.

Apoptosis of SK-Mel-28 cells was measured using annexin V reagents (IncuCyte) following kit protocol after infection with rCVA21 or rCVA21-UnaG. Briefly, the day before infection, SK-Mel-28 cells were seeded in 96-well plates at 2.5 × 10^6^ cells/plate. The next day, 10-fold serial dilutions of viral stocks were prepared in DMEM to give an MOI of 10 to 0.0001 for 100 μL of virus added per well of cells. Annexin V reagents were solubilized with 100 μL DMEM. Then, 100 μL of appropriate diluted virus plus 100 μL annexin V reagent was added per well of cells. Plates were incubated at 37°C with 5% CO_2_. Cell confluence and fluorescence were measured every 2 h for 3 days. The experiment was performed in duplicate wells.

### Electroporation of 293T cells.

293T cells were electroporated with Lonza 4D-Nucleofector using the protocol for HEK293. The next day, cells were harvested and passaged in the presence of 1 μg/mL puromycin (Sigma).

### Quantitative PCR.

Stable integration of miniCMV-CVA21 plasmid DNA into the cell genome was confirmed by SYBR green-based quantitative PCR (qPCR) assay. Genomic DNA from passages 3 and 7 of 293T cells electroporated with miniCMV-CVA21 plasmid was extracted using QIAamp DNA minikit (Qiagen). Primers designed to amplify CVA21 cDNA were 5′-CTTCACTGAGGGCGGATTTAT-3′ and 5′-AAGTCTGGACACGCACTAAC-3′ (IDT). The 18S rRNA coding gene was used as a reference with the following primers: 5′-GTAACCCGTTGAACCCC-3′ and 5′-CCATCCAATCGGTAGTAGCG-3′ (IDT). PowerTrack SYBR green master mix (Applied Biosystems) was added to a 384-well plate with DNA and primers following the manufacturer’s suggested protocol. qPCR was performed by ViiA 7 real-time PCR system (Applied Biosystems) with the cycling steps of 95°C for 2 min for initial denaturation and 40 cycles of 95°C for 15 s and 60°C for 1 min for primer annealing and extension. The threshold cycle (Δ*C_T_*) value (*C_T_*^CVA21^ − C_T_^18S^) was used to compare the level of integrated DNA.

### Statistical analysis.

An unpaired *t* test was used to calculate statistical significance. A *P* value of <0.0001 is extremely significant (****), 0.0001 to 0.001 is extremely significant (***), 0.001 to 0.01 is very significant (**), 0.01 to 0.05 is significant (*), and ≥0.05 is not significant (ns).

### Data availability.

The RNA-Seq NGS raw data collected from two independent experiments (Fig. 2) were uploaded to SRA at https://www.ncbi.nlm.nih.gov/sra, and the BioProject accession number is PRJNA948466.
