# Supplementary material for: Engineering Oncolytic Coxsackievirus A21 with Small Transgenes and Enabling Cell-Mediated Virus Delivery by Integrating Viral cDNA into the Genome
Source: J Virol. 2023 Apr 18;97(5):e00309-23. doi: 10.1128/jvi.00309-23 (PMC10231226; doi:10.1128/jvi.00309-23)
Supplement: Supplemental file 1 — Supplemental material. Download jvi.00309-23-s0001.docx, DOCX file, 0.06 MB [file jvi.00309-23-s0001.docx]

Supplemental materials for:

**Engineering oncolytic coxsackievirus A21 with small transgenes and enabling cell-mediated virus delivery by integrating viral cDNA into the genome**

Miranda Sam^1^, Mohammed Selman^2^#, Weilong Zhao^3^†, Jiwon Jung^1^, Aarron Willingham^1^, Uyen Phan^2^¥, Gary C. Starling^1^€ and Qinshan Gao^1^ *

^1^ Discovery Biologics, Merck & Co., Inc., 213 East Grand Ave., South San Francisco, CA 94080, USA

^2^ Discovery Oncology, Merck & Co., Inc., 213 East Grand Ave., South San Francisco, CA 94080, USA

^3^ Scientific Informatics, Merck & Co., Inc., 770 Sumneytown Pike, West Point, PA 19486, USA

Running Title: CVA21 as a gene delivery vector and a payload.

* Correspondence: Qinshan Gao, Merck & Co., Inc., 213 East Grand Ave., South San Francisco, CA 94080, USA. Email: [Qinshan.Gao@merck.com](mailto:Qinshan.Gao@merck.com). Phone: 650-496-4655.

Present addresses:

#, Mohammed Selman, The Amber and Adam Tarshis Foundation, Los Angeles, California, USA.

†, Weilong Zhao, AbbVie Inc., South San Francisco, California, USA.

¥, Uyen Phan, Foundery Innovations Inc., San Francisco, California, USA.

€, Gary C. Starling, Xyphos Biosciences, an Astellas Company, South San Francisco, California, USA.

Full DNA plasmid sequences:

1. CVA21 cDNA plasmid in pUC19:

gtcgactaatacgactcactatagggttaaaacagctctggggttgttcccaccccagaggcccacgtggcggctagtactctggtattacggtacctttgtacgcctgttttgtatcccttcccccgtaactttagaagcttatcaaaggttcaatagcaggggtacaaaccagtacctctacgaacaagcacttctgtttccccggtgatatcacatagactgtacccacggtcaaaagtgattgatccgttatccgcttgagtacttcgagaagcctagtatcaccttggaatcttcgatgcgttgcgctcaacactctgccccgagtgtagcttaggctgatgagtctgggcactccccaccggcgacggtggcccaggctgcgttggcggcctacccatggctgatgccgtgggacgctagttgtgaacaaggtgtgaagagcctattgagctactcaagagtcctccggcccctgaatgcggctaatcctaaccacggagcaaccgctcacaacccagtgagtaggttgtcgtaatgcgtaagtctgtggcggaaccgactactttgggtgtccgtgtttccctttatattcatactggctgcttatggtgacaatttacaaattgttaccatatagctattggattggccacccagtattgtgcaatatatttgagtgtttctttcataagccttattaacatcacatttttaatcacaataaacagtgcaaatgggggctcaagtttcaacgcaaaagaccggtgcgcacgagaatcaaaacgtggcagccaatggatccaccattaattacactactatcaactattacaaagacagtgcgagtaattccgctactagacaagacctctcccaagatccatcaaaattcacagaaccggttaaggacttaatgttgaaaacagcaccagctctaaactcgcctaacgtggaagcatgtgggtacagtgaccgtgtgaggcaaatcactttaggcaactcgactattactacacaagaagcagccaatgctattgttgcttacggtgaatggcccacttacataaatgattcagaagctaatccggtagatgcacccactgagccagatgttagtagcaaccggttttacaccctagaatcggtgtcttggaagaccacttcaaggggatggtggtggaagttaccagattgtttgaaggacatgggaatgtttggtcagaatatgtactatcactacttggggcgctctggttacaccattcatgtccagtgcaacgcttcaaaatttcaccaaggggcgttaggagtttttctgataccagagtttgtcatggcttgcaacactgagagtaaaacgtcatacgtttcatacatcaatgcaaatcctggtgagagaggcggtgagtttacgaacacctacaatccgttaaatacagacgccagtgagggcagaaagtttgcagcattggattatttgctgggttctggtgttctagcaggaaacgcctttgtgtacccgcaccagatcatcaacctacgtaccaacaacagtgcaacaattgtggtgccatacgtaaactcacttgtgattgattgtatggcaaaacacaataactggggcattgtcatattaccactggcacccttggcctttgccgcaacatcgtcaccacaggtgcctattacagtgaccattgcacccatgtgtacagaattcaatgggttgagaaacatcaccgtcccagtacatcaagggttgccgacaatgaacacacctggttccaatcaattccttacatctgatgacttccagtcgccctgtgccttacctaattttgatgttactccaccaatacacatacccggggaagtaaagaatatgatggaactagctgaaattgacacattgatcccaatgaacgcagtggacgggaaggtgaacacaatggagatgtatcaaataccattgaatgacaatttgagcaaggcacctatattctgtttatccctatcacctgcttctgataaacgactgagccgcaccatgttgggtgaaatcctaaattattacacccattggacggggtccatcaggttcacctttctattttgtggtagtatgatggccactggtaaactgctcctcagctattccccaccgggagctaaaccaccaaccaatcgcaaggatgcaatgctaggcacacacatcatctgggacctagggttacaatccagttgttccatggttgcaccgtggatctccaacacagtgtacagacggtgtgcacgtgatgacttcactgagggcggatttataacttgcttctatcaaactagaattgtggtacctgcttcaacccctaccagtatgttcatgttaggctttgttagtgcgtgtccagacttcagtgtcagactgcttagggacactccccatattagtcaatcgaaactaataggacgtacacaaggcattgaagacctcattgacacagcgataaagaatgccttaagagtgtcccaaccaccctcgacccagtcaactgaagcaactagtggagtgaatagccaggaggtgccagctctaactgctgtggaaacaggagcatctggtcaagcaatccccagtgatgtggtggaaactaggcacgtggtaaattacaaaaccaggtctgaatcgtgtcttgagtcattctttgggagagctgcgtgtgtcacaatcctatccttgaccaactcctccaagagcggagaggagaaaaagcatttcaacatatggaatattacatacaccgacactgtccagttacgcagaaaattagagtttttcacgtattccaggtttgatcttgaaatgacttttgtattcacagagaactatcctagtacagccagtggagaagtgcgaaaccaggtgtaccagatcatgtatattccaccaggggcaccccgcccatcatcctgggatgactacacatggcaatcctcttcaaacccttccatcttctacatgtatggaaatgcacctccacggatgtcaattccttacgtagggattgccaatgcctattcacacttctacgatggctttgcacgggtgccacttgagggtgagaacaccgatgctggcgacacgttttacggtttagtgtccataaatgattttggagttttagcagttagagcagtaaaccgcagtaatccacatacaatacacacatctgtgagagtgtacatgaaaccaaaacacattcggtgttggtgccccagacctcctcgagctgtattatacaggggagagggagtggacatgatatccagtgcaattctacctctgaccaaggtagactcaattaccacttttgggtttggtcatcagaacaaagcagtgtacgttgccggttacaagatttgcaactaccacctagcaaccccaagtgatcacttgaatgcaattagtatgttatgggacagggatttaatggtggtggaatctagagcccagggaactgataccatcgccagatgtagttgcaggtgtggagtttactattgtgaatctaggaggaagtactaccctgtcacttttactggcccaacgtttcgattcatggaagcaaacgactactatccagcaagataccagtctcacatgctgatagggtgcggatttgcagaacccggggactgcggtgggatactgaggtgcactcatggggtaattggtatcattactgcaggaggtgaaggggtagtagcctttgctgacattagagacctctgggtgtatgaagaggaggccatggaacagggaataacaagctacatcgaatctctcggcacagcctttggcgcagggttcacccacacaatcagtgagaaagtgactgaattgacaacaatggttaccagcactatcacagaaaaactactgaaaaacttggtgaaaatagtgtcggctctagtgattgttgtgagaaattatgaggacactaccacgatccttgcaacactagcactactcgggtgtgatatatctccttggcaatggttgaagaagaaggcatgtgacttactagagattccttatgtgatgcgccaaggtgatgggtggatgaagaaattcacagaggcgtgcaatgcagctaaaggcttagagtggattagcaacaaaatttccaagtttatagattggttgaagtgtaaaattatcccagacgctaaggacaaggtggaatttctcaccaagttgaaacagctagacatgttggaaaatcaaattgcaaccatccaccaatcttgccccagccaagaacaacaagagattcttttcaacaatgtgagatggctagcagtccagtcccgtcggtttgcaccattatacgctgtggaggcacgccgaattaacaaaatggagagcacaataaacaattatatacagttcaagagcaaacaccgtattgaaccagtatgtatgctcattcatgggtcaccagggacgggtaaatctatagctacttcattaataggtagagcaatagcagagaaggaaagcacatcagtctattcaatgccacctgacccatctcactttgatggctataaacaacaaggggtagtgattatggacgacctaaaccaaaaccccgatggtatggacatgaaactgttttgccaaatggtatcaacagtggagtttattcctccaatggcctcattagaggagaagggcattttgtttacatctgattatgtcctggcttctaccaactctcattcaattgtaccacccacagtggctcacagtgatgccttaaccagacgatttgcatttgatgtggaggtttacacgatgtctgaacattcagtcaaaggcaaactgaatatggccacggccactcaattgtgtaaggattgtccaacacctgcaaattttaaaaagtgttgccctctcgtttgtggaaaggccttgcaattaatggacaggtacaccagacaaaggttcactgtagatgagattaccacattaatcatgaatgagaaaaacagaagggccaatatcggcaattgcatggaagccttgtttcaaggaccattaaggtataaagatttgaagatcgatgtgaagacagttcccccccctgagtgcatcagtgatttgttacaagcagtggattctcaagaggttagggattactgtgagaagaaaggctggatcgttaacgttactagccagattcaactagaaaggaacatcaatagggccatgactatactccaagctgttaccacattcgcagcagtcgcaggagtagtgtatgtaatgtacaaactcttcgccggtcaacagggtgcatacactggcttgccaaacaaaaaGcccaatgtccctactatcagagtcgctaaagtccaggggccaggatttgactacgcagtggcaatggcaaaaagaaacatagttactgcaaccaccaccaagggtgaatttaccatgctaggggtgcatgataatgtagcaatattgccaacccatgccgctccaggagaaaccattattattgatgggaaagaagtagagatcctagatgccagagccttagaagatcaagcgggaaccaatcttgagatcaccattattactctaaaaagaaatgagaagtttagagacatcagatcacatattcccacccaaattactgaaactaacgatggagtgttgatcgtgaacactagcaagtaccccaatatgtatgtccccgttggtgctgtgaccgaacagggatatcttaatctcagtggacgtcaaactgctcgcactttaatgtacaactttccaacaagggcaggccagtgcggaggaatcatcacttgtactggcaaagtcattgggatgcatgttggcgggaacggttcacatgggtttgcagcagccctcaagcgatcatacttcactcaaaatcagggcgaaatccagtggatgaggtcatcaaaagaagtggggtaccccattataaatgccccatccaagacaaagttagaacccagtgctttccactatgtttttgaaggtgttaaggaaccagctgtactcactaagaatgaccccagactaaaaacagattttgaagaagccatcttttctaaatatgtggggaacaaaattactgaagtggacgagtacatgaaagaagcagtggatcactatgcaggacagttaatgtcactggatatcaacacagaacagatgtgcctggaggatgccatgtacggcaccgatggtcttgaggccctggatcttagcactagtgctggatatccttatgttgcaatggggaaaaagaaaagagacattctaaataaacagaccagagatactaaggagatgcagagacttttagatacctatggaatcaatctaccattagtcacgtacgtgaaagatgaactcaggtcaaagactaaagtggaacaaggaaagtcaagattgattgaagcttccagccttaatgattcagttgcaatgagaatggcctttggcaatctttacgcagctttccacaagaatccaggtgtggtgacaggatcagcagttggttgtgacccagatttgttttggagtaagataccagtgctaatggaagaaaaactcttcgcttttgactacacagggtatgatgcctcactcagccctgcttggtttgaagctcttaaaatggtgttagaaaaaattggatttggcagtagagtagactatatagactacctgaaccactctcaccacctttacaaaaacaagacttattgtgtcaaaggcggcatgccatccggctgctctggcacctcaattttcaactcaatgattaacaacctgatcattaggacgcttttactgagaacctacaagggcatagacttggaccatttaaaaatgattgcctatggtgatgacgtgatagcttcctacccccatgaggttgacgctagtctcctagcccaatcaggaaaagactatggactaaccatgactccagcagataaatcagtaacctttgaaacagtcacatgggagaatgtaacatttctgaaaagatttttcagagcagatgagaagtatccattcctggtgcatccagtgatgccaatgaaagaaattcacgaatcaatcagatggaccaaggaccctagaaacacacaggatcacgtacgctcgttgtgcctattagcttggcacaacggtgaagaagaatacaataaatttttagctaaaatcagaagtgtgccaattggaagagctttattgctcccagagtactctacattgtaccgccgatggctcgactcattttagtaaccctacctcagtcggattggattgggttacactgttgtaggggtaaatttttctttaattcggagaaaaaaaaaaaaaaaaaaaaaaaaagatctacctgcaggCATGCAAGCTTGGCGTAATCATGGTCATAGCTGTTTCCTGTGTGAAATTGTTATCCGCTCACAATTCCACACAACATACGAGCCGGAAGCATAAAGTGTAAAGCCTGGGGTGCCTAATGAGTGAGCTAACTCACATTAATTGCGTTGCGCTCACTGCCCGCTTTCCAGTCGGGAAACCTGTCGTGCCAGCTGCATTAATGAATCGGCCAACGCGCGGGGAGAGGCGGTTTGCGTATTGGGCGCTCTTCCGCTTCCTCGCTCACTGACTCGCTGCGCTCGGTCGTTCGGCTGCGGCGAGCGGTATCAGCTCACTCAAAGGCGGTAATACGGTTATCCACAGAATCAGGGGATAACGCAGGAAAGAACATGTGAGCAAAAGGCCAGCAAAAGGCCAGGAACCGTAAAAAGGCCGCGTTGCTGGCGTTTTTCCATAGGCTCCGCCCCCCTGACGAGCATCACAAAAATCGACGCTCAAGTCAGAGGTGGCGAAACCCGACAGGACTATAAAGATACCAGGCGTTTCCCCCTGGAAGCTCCCTCGTGCGCTCTCCTGTTCCGACCCTGCCGCTTACCGGATACCTGTCCGCCTTTCTCCCTTCGGGAAGCGTGGCGCTTTCTCATAGCTCACGCTGTAGGTATCTCAGTTCGGTGTAGGTCGTTCGCTCCAAGCTGGGCTGTGTGCACGAACCCCCCGTTCAGCCCGACCGCTGCGCCTTATCCGGTAACTATCGTCTTGAGTCCAACCCGGTAAGACACGACTTATCGCCACTGGCAGCAGCCACTGGTAACAGGATTAGCAGAGCGAGGTATGTAGGCGGTGCTACAGAGTTCTTGAAGTGGTGGCCTAACTACGGCTACACTAGAAGAACAGTATTTGGTATCTGCGCTCTGCTGAAGCCAGTTACCTTCGGAAAAAGAGTTGGTAGCTCTTGATCCGGCAAACAAACCACCGCTGGTAGCGGTGGTTTTTTTGTTTGCAAGCAGCAGATTACGCGCAGAAAAAAAGGATCTCAAGAAGATCCTTTGATCTTTTCTACGGGGTCTGACGCTCAGTGGAACGAAAACTCACGTTAAGGGATTTTGGTCATGAGATTATCAAAAAGGATCTTCACCTAGATCCTTTTAAATTAAAAATGAAGTTTTAAATCAATCTAAAGTATATATGAGTAAACTTGGTCTGACAGTTACCAATGCTTAATCAGTGAGGCACCTATCTCAGCGATCTGTCTATTTCGTTCATCCATAGTTGCCTGACTCCCCGTCGTGTAGATAACTACGATACGGGAGGGCTTACCATCTGGCCCCAGTGCTGCAATGATACCGCGAGACCCACGCTCACCGGCTCCAGATTTATCAGCAATAAACCAGCCAGCCGGAAGGGCCGAGCGCAGAAGTGGTCCTGCAACTTTATCCGCCTCCATCCAGTCTATTAATTGTTGCCGGGAAGCTAGAGTAAGTAGTTCGCCAGTTAATAGTTTGCGCAACGTTGTTGCCATTGCTACAGGCATCGTGGTGTCACGCTCGTCGTTTGGTATGGCTTCATTCAGCTCCGGTTCCCAACGATCAAGGCGAGTTACATGATCCCCCATGTTGTGCAAAAAAGCGGTTAGCTCCTTCGGTCCTCCGATCGTTGTCAGAAGTAAGTTGGCCGCAGTGTTATCACTCATGGTTATGGCAGCACTGCATAATTCTCTTACTGTCATGCCATCCGTAAGATGCTTTTCTGTGACTGGTGAGTACTCAACCAAGTCATTCTGAGAATAGTGTATGCGGCGACCGAGTTGCTCTTGCCCGGCGTCAATACGGGATAATACCGCGCCACATAGCAGAACTTTAAAAGTGCTCATCATTGGAAAACGTTCTTCGGGGCGAAAACTCTCAAGGATCTTACCGCTGTTGAGATCCAGTTCGATGTAACCCACTCGTGCACCCAACTGATCTTCAGCATCTTTTACTTTCACCAGCGTTTCTGGGTGAGCAAAAACAGGAAGGCAAAATGCCGCAAAAAAGGGAATAAGGGCGACACGGAAATGTTGAATACTCATACTCTTCCTTTTTCAATATTATTGAAGCATTTATCAGGGTTATTGTCTCATGAGCGGATACATATTTGAATGTATTTAGAAAAATAAACAAATAGGGGTTCCGCGCACATTTCCCCGAAAAGTGCCACCTGACGTCTAAGAAACCATTATTATCATGACATTAACCTATAAAAATAGGCGTATCACGAGGCCCTTTCGTCTCGCGCGTTTCGGTGATGACGGTGAAAACCTCTGACACATGCAGCTCCCGGAGACGGTCACAGCTTGTCTGTAAGCGGATGCCGGGAGCAGACAAGCCCGTCAGGGCGCGTCAGCGGGTGTTGGCGGGTGTCGGGGCTGGCTTAACTATGCGGCATCAGAGCAGATTGTACTGAGAGTGCACCATATGCGGTGTGAAATACCGCACAGATGCGTAAGGAGAAAATACCGCATCAGGCGCCATTCGCCATTCAGGCTGCGCAACTGTTGGGAAGGGCGATCGGTGCGGGCCTCTTCGCTATTACGCCAGCTGGCGAAAGGGGGATGTGCTGCAAGGCGATTAAGTTGGGTAACGCCAGGGTTTTCCCAGTCACGACGTTGTAAAACGACGGCCAGTGAATTCGAGCTCGGTACCCGGGGATCCTCTAGA

1. rCVA21-miR21sponge-1:

gtcgactaatacgactcactatagggttaaaacagctctggggttgttcccaccccagaggcccacgtggcggctagtactctggtattacggtacctttgtacgcctgttttgtatcccttcccccgtaactttagaagcttatcaaaggttcaatagcaggggtacaaaccagtacctctacgaacaagcacttctgtttccccggtgatatcacatagactgtacccacggtcaaaagtgattgatccgttatccgcttgagtacttcgagaagcctagtatcaccttggaatcttcgatgcgttgcgctcaacactctgccccgagtgtagcttaggctgatgagtctgggcactccccaccggcgacggtggcccaggctgcgttggcggcctacccatggctgatgccgtgggacgctagttgtgaacaaggtgtgaagagcctattgagctactcaagagtcctccggcccctgaatgcggctaatcctaaccacggagcaaccgctcacaacccagtgagtaggttgtcgtaatgcgtaagtctgtggcggaaccgactactttgggtgtccgtgtttccctttatattcatactggctgcttatggtgacaatttacaaattgttaccatatagctattggattggccacccagtattgtgcaatatatttgagtgtttctttcataagccttatCaacatcaGaACAtAaGCTacaataaacagtgcaaatgggggctcaagtttcaacgcaaaagaccggtgcgcacgagaatcaaaacgtggcagccaatggatccaccattaattacactactatcaactattacaaagacagtgcgagtaattccgctactagacaagacctctcccaagatccatcaaaattcacagaaccggttaaggacttaatgttgaaaacagcaccagctctaaactcgcctaacgtggaagcatgtgggtacagtgaccgtgtgaggcaaatcactttaggcaactcgactattactacacaagaagcagccaatgctattgttgcttacggtgaatggcccacttacataaatgattcagaagctaatccggtagatgcacccactgagccagatgttagtagcaaccggttttacaccctagaatcggtgtcttggaagaccacttcaaggggatggtggtggaagttaccagattgtttgaaggacatgggaatgtttggtcagaatatgtactatcactacttggggcgctctggttacaccattcatgtccagtgcaacgcttcaaaatttcaccaaggggcgttaggagtttttctgataccagagtttgtcatggcttgcaacactgagagtaaaacgtcatacgtttcatacatcaatgcaaatcctggtgagagaggcggtgagtttacgaacacctacaatccgttaaatacagacgccagtgagggcagaaagtttgcagcattggattatttgctgggttctggtgttctagcaggaaacgcctttgtgtacccgcaccagatcatcaacctacgtaccaacaacagtgcaacaattgtggtgccatacgtaaactcacttgtgattgattgtatggcaaaacacaataactggggcattgtcatattaccactggcacccttggcctttgccgcaacatcgtcaccacaggtgcctattacagtgaccattgcacccatgtgtacagaattcaatgggttgagaaacatcaccgtcccagtacatcaagggttgccgacaatgaacacacctggttccaatcaattccttacatctgatgacttccagtcgccctgtgccttacctaattttgatgttactccaccaatacacatacccggggaagtaaagaatatgatggaactagctgaaattgacacattgatcccaatgaacgcagtggacgggaaggtgaacacaatggagatgtatcaaataccattgaatgacaatttgagcaaggcacctatattctgtttatccctatcacctgcttctgataaacgactgagccgcaccatgttgggtgaaatcctaaattattacacccattggacggggtccatcaggttcacctttctattttgtggtagtatgatggccactggtaaactgctcctcagctattccccaccgggagctaaaccaccaaccaatcgcaaggatgcaatgctaggcacacacatcatctgggacctagggttacaatccagttgttccatggttgcaccgtggatctccaacacagtgtacagacggtgtgcacgtgatgacttcactgagggcggatttataacttgcttctatcaaactagaattgtggtacctgcttcaacccctaccagtatgttcatgttaggctttgttagtgcgtgtccagacttcagtgtcagactgcttagggacactccccatattagtcaatcgaaactaataggacgtacacaaggcattgaagacctcattgacacagcgataaagaatgccttaagagtgtcccaaccaccctcgacccagtcaactgaagcaactagtggagtgaatagccaggaggtgccagctctaactgctgtggaaacaggagcatctggtcaagcaatccccagtgatgtggtggaaactaggcacgtggtaaattacaaaaccaggtctgaatcgtgtcttgagtcattctttgggagagctgcgtgtgtcacaatcctatccttgaccaactcctccaagagcggagaggagaaaaagcatttcaacatatggaatattacatacaccgacactgtccagttacgcagaaaattagagtttttcacgtattccaggtttgatcttgaaatgacttttgtattcacagagaactatcctagtacagccagtggagaagtgcgaaaccaggtgtaccagatcatgtatattccaccaggggcaccccgcccatcatcctgggatgactacacatggcaatcctcttcaaacccttccatcttctacatgtatggaaatgcacctccacggatgtcaattccttacgtagggattgccaatgcctattcacacttctacgatggctttgcacgggtgccacttgagggtgagaacaccgatgctggcgacacgttttacggtttagtgtccataaatgattttggagttttagcagttagagcagtaaaccgcagtaatccacatacaatacacacatctgtgagagtgtacatgaaaccaaaacacattcggtgttggtgccccagacctcctcgagctgtattatacaggggagagggagtggacatgatatccagtgcaattctacctctgaccaaggtagactcaattaccacttttgggtttggtcatcagaacaaagcagtgtacgttgccggttacaagatttgcaactaccacctagcaaccccaagtgatcacttgaatgcaattagtatgttatgggacagggatttaatggtggtggaatctagagcccagggaactgataccatcgccagatgtagttgcaggtgtggagtttactattgtgaatctaggaggaagtactaccctgtcacttttactggcccaacgtttcgattcatggaagcaaacgactactatccagcaagataccagtctcacatgctgatagggtgcggatttgcagaacccggggactgcggtgggatactgaggtgcactcatggggtaattggtatcattactgcaggaggtgaaggggtagtagcctttgctgacattagagacctctgggtgtatgaagaggaggccatggaacagggaataacaagctacatcgaatctctcggcacagcctttggcgcagggttcacccacacaatcagtgagaaagtgactgaattgacaacaatggttaccagcactatcacagaaaaactactgaaaaacttggtgaaaatagtgtcggctctagtgattgttgtgagaaattatgaggacactaccacgatccttgcaacactagcactactcgggtgtgatatatctccttggcaatggttgaagaagaaggcatgtgacttactagagattccttatgtgatgcgccaaggtgatgggtggatgaagaaattcacagaggcgtgcaatgcagctaaaggcttagagtggattagcaacaaaatttccaagtttatagattggttgaagtgtaaaattatcccagacgctaaggacaaggtggaatttctcaccaagttgaaacagctagacatgttggaaaatcaaattgcaaccatccaccaatcttgccccagccaagaacaacaagagattcttttcaacaatgtgagatggctagcagtccagtcccgtcggtttgcaccattatacgctgtggaggcacgccgaattaacaaaatggagagcacaataaacaattatatacagttcaagagcaaacaccgtattgaaccagtatgtatgctcattcatgggtcaccagggacgggtaaatctatagctacttcattaataggtagagcaatagcagagaaggaaagcacatcagtctattcaatgccacctgacccatctcactttgatggctataaacaacaaggggtagtgattatggacgacctaaaccaaaaccccgatggtatggacatgaaactgttttgccaaatggtatcaacagtggagtttattcctccaatggcctcattagaggagaagggcattttgtttacatctgattatgtcctggcttctaccaactctcattcaattgtaccacccacagtggctcacagtgatgccttaaccagacgatttgcatttgatgtggaggtttacacgatgtctgaacattcagtcaaaggcaaactgaatatggccacggccactcaattgtgtaaggattgtccaacacctgcaaattttaaaaagtgttgccctctcgtttgtggaaaggccttgcaattaatggacaggtacaccagacaaaggttcactgtagatgagattaccacattaatcatgaatgagaaaaacagaagggccaatatcggcaattgcatggaagccttgtttcaaggaccattaaggtataaagatttgaagatcgatgtgaagacagttcccccccctgagtgcatcagtgatttgttacaagcagtggattctcaagaggttagggattactgtgagaagaaaggctggatcgttaacgttactagccagattcaactagaaaggaacatcaatagggccatgactatactccaagctgttaccacattcgcagcagtcgcaggagtagtgtatgtaatgtacaaactcttcgccggtcaacagggtgcatacactggcttgccaaacaaaaaacccaatgtccctactatcagagtcgctaaagtccaggggccaggatttgactacgcagtggcaatggcaaaaagaaacatagttactgcaaccaccaccaagggtgaatttaccatgctaggggtgcatgataatgtagcaatattgccaacccatgccgctccaggagaaaccattattattgatgggaaagaagtagagatcctagatgccagagccttagaagatcaagcgggaaccaatcttgagatcaccattattactctaaaaagaaatgagaagtttagagacatcagatcacatattcccacccaaattactgaaactaacgatggagtgttgatcgtgaacactagcaagtaccccaatatgtatgtccccgttggtgctgtgaccgaacagggatatcttaatctcagtggacgtcaaactgctcgcactttaatgtacaactttccaacaagggcaggccagtgcggaggaatcatcacttgtactggcaaagtcattgggatgcatgttggcgggaacggttcacatgggtttgcagcagccctcaagcgatcatacttcactcaaaatcagggcgaaatccagtggatgaggtcatcaaaagaagtggggtaccccattataaatgccccatccaagacaaagttagaacccagtgctttccactatgtttttgaaggtgttaaggaaccagctgtactcactaagaatgaccccagactaaaaacagattttgaagaagccatcttttctaaatatgtggggaacaaaattactgaagtggacgagtacatgaaagaagcagtggatcactatgcaggacagttaatgtcactggatatcaacacagaacagatgtgcctggaggatgccatgtacggcaccgatggtcttgaggccctggatcttagcactagtgctggatatccttatgttgcaatggggaaaaagaaaagagacattctaaataaacagaccagagatactaaggagatgcagagacttttagatacctatggaatcaatctaccattagtcacgtacgtgaaagatgaactcaggtcaaagactaaagtggaacaaggaaagtcaagattgattgaagcttccagccttaatgattcagttgcaatgagaatggcctttggcaatctttacgcagctttccacaagaatccaggtgtggtgacaggatcagcagttggttgtgacccagatttgttttggagtaagataccagtgctaatggaagaaaaactcttcgcttttgactacacagggtatgatgcctcactcagccctgcttggtttgaagctcttaaaatggtgttagaaaaaattggatttggcagtagagtagactatatagactacctgaaccactctcaccacctttacaaaaacaagacttattgtgtcaaaggcggcatgccatccggctgctctggcacctcaattttcaactcaatgattaacaacctgatcattaggacgcttttactgagaacctacaagggcatagacttggaccatttaaaaatgattgcctatggtgatgacgtgatagcttcctacccccatgaggttgacgctagtctcctagcccaatcaggaaaagactatggactaaccatgactccagcagataaatcagtaacctttgaaacagtcacatgggagaatgtaacatttctgaaaagatttttcagagcagatgagaagtatccattcctggtgcatccagtgatgccaatgaaagaaattcacgaatcaatcagatggaccaaggaccctagaaacacacaggatcacgtacgctcgttgtgcctattagcttggcacaacggtgaagaagaatacaataaatttttagctaaaatcagaagtgtgccaattggaagagctttattgctcccagagtactctacattgtaccgccgatggctcgactcattttagtaaccctacctcagtcggattggattgggttacactgttgtaggggtaaatttttctttaattcggagaaaaaaaaaaaaaaaaaaaaaaaaagatctacctgcaggCATGCAAGCTTGGCGTAATCATGGTCATAGCTGTTTCCTGTGTGAAATTGTTATCCGCTCACAATTCCACACAACATACGAGCCGGAAGCATAAAGTGTAAAGCCTGGGGTGCCTAATGAGTGAGCTAACTCACATTAATTGCGTTGCGCTCACTGCCCGCTTTCCAGTCGGGAAACCTGTCGTGCCAGCTGCATTAATGAATCGGCCAACGCGCGGGGAGAGGCGGTTTGCGTATTGGGCGCTCTTCCGCTTCCTCGCTCACTGACTCGCTGCGCTCGGTCGTTCGGCTGCGGCGAGCGGTATCAGCTCACTCAAAGGCGGTAATACGGTTATCCACAGAATCAGGGGATAACGCAGGAAAGAACATGTGAGCAAAAGGCCAGCAAAAGGCCAGGAACCGTAAAAAGGCCGCGTTGCTGGCGTTTTTCCATAGGCTCCGCCCCCCTGACGAGCATCACAAAAATCGACGCTCAAGTCAGAGGTGGCGAAACCCGACAGGACTATAAAGATACCAGGCGTTTCCCCCTGGAAGCTCCCTCGTGCGCTCTCCTGTTCCGACCCTGCCGCTTACCGGATACCTGTCCGCCTTTCTCCCTTCGGGAAGCGTGGCGCTTTCTCATAGCTCACGCTGTAGGTATCTCAGTTCGGTGTAGGTCGTTCGCTCCAAGCTGGGCTGTGTGCACGAACCCCCCGTTCAGCCCGACCGCTGCGCCTTATCCGGTAACTATCGTCTTGAGTCCAACCCGGTAAGACACGACTTATCGCCACTGGCAGCAGCCACTGGTAACAGGATTAGCAGAGCGAGGTATGTAGGCGGTGCTACAGAGTTCTTGAAGTGGTGGCCTAACTACGGCTACACTAGAAGAACAGTATTTGGTATCTGCGCTCTGCTGAAGCCAGTTACCTTCGGAAAAAGAGTTGGTAGCTCTTGATCCGGCAAACAAACCACCGCTGGTAGCGGTGGTTTTTTTGTTTGCAAGCAGCAGATTACGCGCAGAAAAAAAGGATCTCAAGAAGATCCTTTGATCTTTTCTACGGGGTCTGACGCTCAGTGGAACGAAAACTCACGTTAAGGGATTTTGGTCATGAGATTATCAAAAAGGATCTTCACCTAGATCCTTTTAAATTAAAAATGAAGTTTTAAATCAATCTAAAGTATATATGAGTAAACTTGGTCTGACAGTTACCAATGCTTAATCAGTGAGGCACCTATCTCAGCGATCTGTCTATTTCGTTCATCCATAGTTGCCTGACTCCCCGTCGTGTAGATAACTACGATACGGGAGGGCTTACCATCTGGCCCCAGTGCTGCAATGATACCGCGAGACCCACGCTCACCGGCTCCAGATTTATCAGCAATAAACCAGCCAGCCGGAAGGGCCGAGCGCAGAAGTGGTCCTGCAACTTTATCCGCCTCCATCCAGTCTATTAATTGTTGCCGGGAAGCTAGAGTAAGTAGTTCGCCAGTTAATAGTTTGCGCAACGTTGTTGCCATTGCTACAGGCATCGTGGTGTCACGCTCGTCGTTTGGTATGGCTTCATTCAGCTCCGGTTCCCAACGATCAAGGCGAGTTACATGATCCCCCATGTTGTGCAAAAAAGCGGTTAGCTCCTTCGGTCCTCCGATCGTTGTCAGAAGTAAGTTGGCCGCAGTGTTATCACTCATGGTTATGGCAGCACTGCATAATTCTCTTACTGTCATGCCATCCGTAAGATGCTTTTCTGTGACTGGTGAGTACTCAACCAAGTCATTCTGAGAATAGTGTATGCGGCGACCGAGTTGCTCTTGCCCGGCGTCAATACGGGATAATACCGCGCCACATAGCAGAACTTTAAAAGTGCTCATCATTGGAAAACGTTCTTCGGGGCGAAAACTCTCAAGGATCTTACCGCTGTTGAGATCCAGTTCGATGTAACCCACTCGTGCACCCAACTGATCTTCAGCATCTTTTACTTTCACCAGCGTTTCTGGGTGAGCAAAAACAGGAAGGCAAAATGCCGCAAAAAAGGGAATAAGGGCGACACGGAAATGTTGAATACTCATACTCTTCCTTTTTCAATATTATTGAAGCATTTATCAGGGTTATTGTCTCATGAGCGGATACATATTTGAATGTATTTAGAAAAATAAACAAATAGGGGTTCCGCGCACATTTCCCCGAAAAGTGCCACCTGACGTCTAAGAAACCATTATTATCATGACATTAACCTATAAAAATAGGCGTATCACGAGGCCCTTTCGTCTCGCGCGTTTCGGTGATGACGGTGAAAACCTCTGACACATGCAGCTCCCGGAGACGGTCACAGCTTGTCTGTAAGCGGATGCCGGGAGCAGACAAGCCCGTCAGGGCGCGTCAGCGGGTGTTGGCGGGTGTCGGGGCTGGCTTAACTATGCGGCATCAGAGCAGATTGTACTGAGAGTGCACCATATGCGGTGTGAAATACCGCACAGATGCGTAAGGAGAAAATACCGCATCAGGCGCCATTCGCCATTCAGGCTGCGCAACTGTTGGGAAGGGCGATCGGTGCGGGCCTCTTCGCTATTACGCCAGCTGGCGAAAGGGGGATGTGCTGCAAGGCGATTAAGTTGGGTAACGCCAGGGTTTTCCCAGTCACGACGTTGTAAAACGACGGCCAGTGAATTCGAGCTCGGTACCCGGGGATCCTCTAGA

1. rCVA21-miR21sponge-3:

gtcgactaatacgactcactatagggttaaaacagctctggggttgttcccaccccagaggcccacgtggcggctagtactctggtattacggtacctttgtacgcctgttttgtatcccttcccccgtaactttagaagcttatcaaaggttcaatagcaggggtacaaaccagtacctctacgaacaagcacttctgtttccccggtgatatcacatagactgtacccacggtcaaaagtgattgatccgttatccgcttgagtacttcgagaagcctagtatcaccttggaatcttcgatgcgttgcgctcaacactctgccccgagtgtagcttaggctgatgagtctgggcactccccaccggcgacggtggcccaggctgcgttggcggcctacccatggctgatgccgtgggacgctagttgtgaacaaggtgtgaagagcctattgagctactcaagagtcctccggcccctgaatgcggctaatcctaaccacggagcaaccgctcacaacccagtgagtaggttgtcgtaatgcgtaagtctgtggcggaaccgactactttgggtgtccgtgtttccctttatattcatactggctgcttatggtgacaatttacaaattgttaccatatagctattggattggccacccagtatTCAACATCAGAAcataagcTAtatttTCAACATCAGAAcataagcTAtatCaacatcaGaACAtAaGCTacaataaacagtgcaaatgggggctcaagtttcaacgcaaaagaccggtgcgcacgagaatcaaaacgtggcagccaatggatccaccattaattacactactatcaactattacaaagacagtgcgagtaattccgctactagacaagacctctcccaagatccatcaaaattcacagaaccggttaaggacttaatgttgaaaacagcaccagctctaaactcgcctaacgtggaagcatgtgggtacagtgaccgtgtgaggcaaatcactttaggcaactcgactattactacacaagaagcagccaatgctattgttgcttacggtgaatggcccacttacataaatgattcagaagctaatccggtagatgcacccactgagccagatgttagtagcaaccggttttacaccctagaatcggtgtcttggaagaccacttcaaggggatggtggtggaagttaccagattgtttgaaggacatgggaatgtttggtcagaatatgtactatcactacttggggcgctctggttacaccattcatgtccagtgcaacgcttcaaaatttcaccaaggggcgttaggagtttttctgataccagagtttgtcatggcttgcaacactgagagtaaaacgtcatacgtttcatacatcaatgcaaatcctggtgagagaggcggtgagtttacgaacacctacaatccgttaaatacagacgccagtgagggcagaaagtttgcagcattggattatttgctgggttctggtgttctagcaggaaacgcctttgtgtacccgcaccagatcatcaacctacgtaccaacaacagtgcaacaattgtggtgccatacgtaaactcacttgtgattgattgtatggcaaaacacaataactggggcattgtcatattaccactggcacccttggcctttgccgcaacatcgtcaccacaggtgcctattacagtgaccattgcacccatgtgtacagaattcaatgggttgagaaacatcaccgtcccagtacatcaagggttgccgacaatgaacacacctggttccaatcaattccttacatctgatgacttccagtcgccctgtgccttacctaattttgatgttactccaccaatacacatacccggggaagtaaagaatatgatggaactagctgaaattgacacattgatcccaatgaacgcagtggacgggaaggtgaacacaatggagatgtatcaaataccattgaatgacaatttgagcaaggcacctatattctgtttatccctatcacctgcttctgataaacgactgagccgcaccatgttgggtgaaatcctaaattattacacccattggacggggtccatcaggttcacctttctattttgtggtagtatgatggccactggtaaactgctcctcagctattccccaccgggagctaaaccaccaaccaatcgcaaggatgcaatgctaggcacacacatcatctgggacctagggttacaatccagttgttccatggttgcaccgtggatctccaacacagtgtacagacggtgtgcacgtgatgacttcactgagggcggatttataacttgcttctatcaaactagaattgtggtacctgcttcaacccctaccagtatgttcatgttaggctttgttagtgcgtgtccagacttcagtgtcagactgcttagggacactccccatattagtcaatcgaaactaataggacgtacacaaggcattgaagacctcattgacacagcgataaagaatgccttaagagtgtcccaaccaccctcgacccagtcaactgaagcaactagtggagtgaatagccaggaggtgccagctctaactgctgtggaaacaggagcatctggtcaagcaatccccagtgatgtggtggaaactaggcacgtggtaaattacaaaaccaggtctgaatcgtgtcttgagtcattctttgggagagctgcgtgtgtcacaatcctatccttgaccaactcctccaagagcggagaggagaaaaagcatttcaacatatggaatattacatacaccgacactgtccagttacgcagaaaattagagtttttcacgtattccaggtttgatcttgaaatgacttttgtattcacagagaactatcctagtacagccagtggagaagtgcgaaaccaggtgtaccagatcatgtatattccaccaggggcaccccgcccatcatcctgggatgactacacatggcaatcctcttcaaacccttccatcttctacatgtatggaaatgcacctccacggatgtcaattccttacgtagggattgccaatgcctattcacacttctacgatggctttgcacgggtgccacttgagggtgagaacaccgatgctggcgacacgttttacggtttagtgtccataaatgattttggagttttagcagttagagcagtaaaccgcagtaatccacatacaatacacacatctgtgagagtgtacatgaaaccaaaacacattcggtgttggtgccccagacctcctcgagctgtattatacaggggagagggagtggacatgatatccagtgcaattctacctctgaccaaggtagactcaattaccacttttgggtttggtcatcagaacaaagcagtgtacgttgccggttacaagatttgcaactaccacctagcaaccccaagtgatcacttgaatgcaattagtatgttatgggacagggatttaatggtggtggaatctagagcccagggaactgataccatcgccagatgtagttgcaggtgtggagtttactattgtgaatctaggaggaagtactaccctgtcacttttactggcccaacgtttcgattcatggaagcaaacgactactatccagcaagataccagtctcacatgctgatagggtgcggatttgcagaacccggggactgcggtgggatactgaggtgcactcatggggtaattggtatcattactgcaggaggtgaaggggtagtagcctttgctgacattagagacctctgggtgtatgaagaggaggccatggaacagggaataacaagctacatcgaatctctcggcacagcctttggcgcagggttcacccacacaatcagtgagaaagtgactgaattgacaacaatggttaccagcactatcacagaaaaactactgaaaaacttggtgaaaatagtgtcggctctagtgattgttgtgagaaattatgaggacactaccacgatccttgcaacactagcactactcgggtgtgatatatctccttggcaatggttgaagaagaaggcatgtgacttactagagattccttatgtgatgcgccaaggtgatgggtggatgaagaaattcacagaggcgtgcaatgcagctaaaggcttagagtggattagcaacaaaatttccaagtttatagattggttgaagtgtaaaattatcccagacgctaaggacaaggtggaatttctcaccaagttgaaacagctagacatgttggaaaatcaaattgcaaccatccaccaatcttgccccagccaagaacaacaagagattcttttcaacaatgtgagatggctagcagtccagtcccgtcggtttgcaccattatacgctgtggaggcacgccgaattaacaaaatggagagcacaataaacaattatatacagttcaagagcaaacaccgtattgaaccagtatgtatgctcattcatgggtcaccagggacgggtaaatctatagctacttcattaataggtagagcaatagcagagaaggaaagcacatcagtctattcaatgccacctgacccatctcactttgatggctataaacaacaaggggtagtgattatggacgacctaaaccaaaaccccgatggtatggacatgaaactgttttgccaaatggtatcaacagtggagtttattcctccaatggcctcattagaggagaagggcattttgtttacatctgattatgtcctggcttctaccaactctcattcaattgtaccacccacagtggctcacagtgatgccttaaccagacgatttgcatttgatgtggaggtttacacgatgtctgaacattcagtcaaaggcaaactgaatatggccacggccactcaattgtgtaaggattgtccaacacctgcaaattttaaaaagtgttgccctctcgtttgtggaaaggccttgcaattaatggacaggtacaccagacaaaggttcactgtagatgagattaccacattaatcatgaatgagaaaaacagaagggccaatatcggcaattgcatggaagccttgtttcaaggaccattaaggtataaagatttgaagatcgatgtgaagacagttcccccccctgagtgcatcagtgatttgttacaagcagtggattctcaagaggttagggattactgtgagaagaaaggctggatcgttaacgttactagccagattcaactagaaaggaacatcaatagggccatgactatactccaagctgttaccacattcgcagcagtcgcaggagtagtgtatgtaatgtacaaactcttcgccggtcaacagggtgcatacactggcttgccaaacaaaaaacccaatgtccctactatcagagtcgctaaagtccaggggccaggatttgactacgcagtggcaatggcaaaaagaaacatagttactgcaaccaccaccaagggtgaatttaccatgctaggggtgcatgataatgtagcaatattgccaacccatgccgctccaggagaaaccattattattgatgggaaagaagtagagatcctagatgccagagccttagaagatcaagcgggaaccaatcttgagatcaccattattactctaaaaagaaatgagaagtttagagacatcagatcacatattcccacccaaattactgaaactaacgatggagtgttgatcgtgaacactagcaagtaccccaatatgtatgtccccgttggtgctgtgaccgaacagggatatcttaatctcagtggacgtcaaactgctcgcactttaatgtacaactttccaacaagggcaggccagtgcggaggaatcatcacttgtactggcaaagtcattgggatgcatgttggcgggaacggttcacatgggtttgcagcagccctcaagcgatcatacttcactcaaaatcagggcgaaatccagtggatgaggtcatcaaaagaagtggggtaccccattataaatgccccatccaagacaaagttagaacccagtgctttccactatgtttttgaaggtgttaaggaaccagctgtactcactaagaatgaccccagactaaaaacagattttgaagaagccatcttttctaaatatgtggggaacaaaattactgaagtggacgagtacatgaaagaagcagtggatcactatgcaggacagttaatgtcactggatatcaacacagaacagatgtgcctggaggatgccatgtacggcaccgatggtcttgaggccctggatcttagcactagtgctggatatccttatgttgcaatggggaaaaagaaaagagacattctaaataaacagaccagagatactaaggagatgcagagacttttagatacctatggaatcaatctaccattagtcacgtacgtgaaagatgaactcaggtcaaagactaaagtggaacaaggaaagtcaagattgattgaagcttccagccttaatgattcagttgcaatgagaatggcctttggcaatctttacgcagctttccacaagaatccaggtgtggtgacaggatcagcagttggttgtgacccagatttgttttggagtaagataccagtgctaatggaagaaaaactcttcgcttttgactacacagggtatgatgcctcactcagccctgcttggtttgaagctcttaaaatggtgttagaaaaaattggatttggcagtagagtagactatatagactacctgaaccactctcaccacctttacaaaaacaagacttattgtgtcaaaggcggcatgccatccggctgctctggcacctcaattttcaactcaatgattaacaacctgatcattaggacgcttttactgagaacctacaagggcatagacttggaccatttaaaaatgattgcctatggtgatgacgtgatagcttcctacccccatgaggttgacgctagtctcctagcccaatcaggaaaagactatggactaaccatgactccagcagataaatcagtaacctttgaaacagtcacatgggagaatgtaacatttctgaaaagatttttcagagcagatgagaagtatccattcctggtgcatccagtgatgccaatgaaagaaattcacgaatcaatcagatggaccaaggaccctagaaacacacaggatcacgtacgctcgttgtgcctattagcttggcacaacggtgaagaagaatacaataaatttttagctaaaatcagaagtgtgccaattggaagagctttattgctcccagagtactctacattgtaccgccgatggctcgactcattttagtaaccctacctcagtcggattggattgggttacactgttgtaggggtaaatttttctttaattcggagaaaaaaaaaaaaaaaaaaaaaaaaagatctacctgcaggCATGCAAGCTTGGCGTAATCATGGTCATAGCTGTTTCCTGTGTGAAATTGTTATCCGCTCACAATTCCACACAACATACGAGCCGGAAGCATAAAGTGTAAAGCCTGGGGTGCCTAATGAGTGAGCTAACTCACATTAATTGCGTTGCGCTCACTGCCCGCTTTCCAGTCGGGAAACCTGTCGTGCCAGCTGCATTAATGAATCGGCCAACGCGCGGGGAGAGGCGGTTTGCGTATTGGGCGCTCTTCCGCTTCCTCGCTCACTGACTCGCTGCGCTCGGTCGTTCGGCTGCGGCGAGCGGTATCAGCTCACTCAAAGGCGGTAATACGGTTATCCACAGAATCAGGGGATAACGCAGGAAAGAACATGTGAGCAAAAGGCCAGCAAAAGGCCAGGAACCGTAAAAAGGCCGCGTTGCTGGCGTTTTTCCATAGGCTCCGCCCCCCTGACGAGCATCACAAAAATCGACGCTCAAGTCAGAGGTGGCGAAACCCGACAGGACTATAAAGATACCAGGCGTTTCCCCCTGGAAGCTCCCTCGTGCGCTCTCCTGTTCCGACCCTGCCGCTTACCGGATACCTGTCCGCCTTTCTCCCTTCGGGAAGCGTGGCGCTTTCTCATAGCTCACGCTGTAGGTATCTCAGTTCGGTGTAGGTCGTTCGCTCCAAGCTGGGCTGTGTGCACGAACCCCCCGTTCAGCCCGACCGCTGCGCCTTATCCGGTAACTATCGTCTTGAGTCCAACCCGGTAAGACACGACTTATCGCCACTGGCAGCAGCCACTGGTAACAGGATTAGCAGAGCGAGGTATGTAGGCGGTGCTACAGAGTTCTTGAAGTGGTGGCCTAACTACGGCTACACTAGAAGAACAGTATTTGGTATCTGCGCTCTGCTGAAGCCAGTTACCTTCGGAAAAAGAGTTGGTAGCTCTTGATCCGGCAAACAAACCACCGCTGGTAGCGGTGGTTTTTTTGTTTGCAAGCAGCAGATTACGCGCAGAAAAAAAGGATCTCAAGAAGATCCTTTGATCTTTTCTACGGGGTCTGACGCTCAGTGGAACGAAAACTCACGTTAAGGGATTTTGGTCATGAGATTATCAAAAAGGATCTTCACCTAGATCCTTTTAAATTAAAAATGAAGTTTTAAATCAATCTAAAGTATATATGAGTAAACTTGGTCTGACAGTTACCAATGCTTAATCAGTGAGGCACCTATCTCAGCGATCTGTCTATTTCGTTCATCCATAGTTGCCTGACTCCCCGTCGTGTAGATAACTACGATACGGGAGGGCTTACCATCTGGCCCCAGTGCTGCAATGATACCGCGAGACCCACGCTCACCGGCTCCAGATTTATCAGCAATAAACCAGCCAGCCGGAAGGGCCGAGCGCAGAAGTGGTCCTGCAACTTTATCCGCCTCCATCCAGTCTATTAATTGTTGCCGGGAAGCTAGAGTAAGTAGTTCGCCAGTTAATAGTTTGCGCAACGTTGTTGCCATTGCTACAGGCATCGTGGTGTCACGCTCGTCGTTTGGTATGGCTTCATTCAGCTCCGGTTCCCAACGATCAAGGCGAGTTACATGATCCCCCATGTTGTGCAAAAAAGCGGTTAGCTCCTTCGGTCCTCCGATCGTTGTCAGAAGTAAGTTGGCCGCAGTGTTATCACTCATGGTTATGGCAGCACTGCATAATTCTCTTACTGTCATGCCATCCGTAAGATGCTTTTCTGTGACTGGTGAGTACTCAACCAAGTCATTCTGAGAATAGTGTATGCGGCGACCGAGTTGCTCTTGCCCGGCGTCAATACGGGATAATACCGCGCCACATAGCAGAACTTTAAAAGTGCTCATCATTGGAAAACGTTCTTCGGGGCGAAAACTCTCAAGGATCTTACCGCTGTTGAGATCCAGTTCGATGTAACCCACTCGTGCACCCAACTGATCTTCAGCATCTTTTACTTTCACCAGCGTTTCTGGGTGAGCAAAAACAGGAAGGCAAAATGCCGCAAAAAAGGGAATAAGGGCGACACGGAAATGTTGAATACTCATACTCTTCCTTTTTCAATATTATTGAAGCATTTATCAGGGTTATTGTCTCATGAGCGGATACATATTTGAATGTATTTAGAAAAATAAACAAATAGGGGTTCCGCGCACATTTCCCCGAAAAGTGCCACCTGACGTCTAAGAAACCATTATTATCATGACATTAACCTATAAAAATAGGCGTATCACGAGGCCCTTTCGTCTCGCGCGTTTCGGTGATGACGGTGAAAACCTCTGACACATGCAGCTCCCGGAGACGGTCACAGCTTGTCTGTAAGCGGATGCCGGGAGCAGACAAGCCCGTCAGGGCGCGTCAGCGGGTGTTGGCGGGTGTCGGGGCTGGCTTAACTATGCGGCATCAGAGCAGATTGTACTGAGAGTGCACCATATGCGGTGTGAAATACCGCACAGATGCGTAAGGAGAAAATACCGCATCAGGCGCCATTCGCCATTCAGGCTGCGCAACTGTTGGGAAGGGCGATCGGTGCGGGCCTCTTCGCTATTACGCCAGCTGGCGAAAGGGGGATGTGCTGCAAGGCGATTAAGTTGGGTAACGCCAGGGTTTTCCCAGTCACGACGTTGTAAAACGACGGCCAGTGAATTCGAGCTCGGTACCCGGGGATCCTCTAGA

1. rCVA21-GFP-102-TC:

gtcgactaatacgactcactatagggttaaaacagctctggggttgttcccaccccagaggcccacgtggcggctagtactctggtattacggtacctttgtacgcctgttttgtatcccttcccccgtaactttagaagcttatcaaaggttcaatagcaggggtacaaaccagtacctctacgaacaagcacttctgtttccccggtgatatcacatagactgtacccacggtcaaaagtgattgatccgttatccgcttgagtacttcgagaagcctagtatcaccttggaatcttcgatgcgttgcgctcaacactctgccccgagtgtagcttaggctgatgagtctgggcactccccaccggcgacggtggcccaggctgcgttggcggcctacccatggctgatgccgtgggacgctagttgtgaacaaggtgtgaagagcctattgagctactcaagagtcctccggcccctgaatgcggctaatcctaaccacggagcaaccgctcacaacccagtgagtaggttgtcgtaatgcgtaagtctgtggcggaaccgactactttgggtgtccgtgtttccctttatattcatactggctgcttatggtgacaatttacaaattgttaccatatagctattggattggccacccagtattgtgcaatatatttgagtgtttctttcataagccttattaacatcacatttttaatcacaataaacagtgcaaatgGTGAGCAAGGGCGAGGAGCTGTTCACCGGGGTGGTGCCCATCCTGGTCGAGCTGGACGGCGACGTAAACGGCCACAAGTTCAGCGTGTCCGGCGAGGGCGAGGGCGATGCCACCTACGGCAAGCTGACCCTGAAGTTCATCTGCACCACCGGCAAGCTGCCCGTGCCCTGGCCCACCCTCGTGACCACCCTGACCTACGGCGTGCAGTGCTTCAGCCGCTACCCCGACCACATGAAGCAGCACGACTTCTTCAAGTCCGCCATGCCCGAAGGCTACGTCCAGGAGCGCACCATCTTCTTCAAGTTCCTGAACTGCTGtCCCGGCTGtTGCATGGAGCCCGCTTTGTTTCAAGGAGCTCAAgtttcaacgcaaaagaccggtgcgcacgagaatcaaaacgtggcagccaatggatccaccattaattacactactatcaactattacaaagacagtgcgagtaattccgctactagacaagacctctcccaagatccatcaaaattcacagaaccggttaaggacttaatgttgaaaacagcaccagctctaaactcgcctaacgtggaagcatgtgggtacagtgaccgtgtgaggcaaatcactttaggcaactcgactattactacacaagaagcagccaatgctattgttgcttacggtgaatggcccacttacataaatgattcagaagctaatccggtagatgcacccactgagccagatgttagtagcaaccggttttacaccctagaatcggtgtcttggaagaccacttcaaggggatggtggtggaagttaccagattgtttgaaggacatgggaatgtttggtcagaatatgtactatcactacttggggcgctctggttacaccattcatgtccagtgcaacgcttcaaaatttcaccaaggggcgttaggagtttttctgataccagagtttgtcatggcttgcaacactgagagtaaaacgtcatacgtttcatacatcaatgcaaatcctggtgagagaggcggtgagtttacgaacacctacaatccgttaaatacagacgccagtgagggcagaaagtttgcagcattggattatttgctgggttctggtgttctagcaggaaacgcctttgtgtacccgcaccagatcatcaacctacgtaccaacaacagtgcaacaattgtggtgccatacgtaaactcacttgtgattgattgtatggcaaaacacaataactggggcattgtcatattaccactggcacccttggcctttgccgcaacatcgtcaccacaggtgcctattacagtgaccattgcacccatgtgtacagaattcaatgggttgagaaacatcaccgtcccagtacatcaagggttgccgacaatgaacacacctggttccaatcaattccttacatctgatgacttccagtcgccctgtgccttacctaattttgatgttactccaccaatacacatacccggggaagtaaagaatatgatggaactagctgaaattgacacattgatcccaatgaacgcagtggacgggaaggtgaacacaatggagatgtatcaaataccattgaatgacaatttgagcaaggcacctatattctgtttatccctatcacctgcttctgataaacgactgagccgcaccatgttgggtgaaatcctaaattattacacccattggacggggtccatcaggttcacctttctattttgtggtagtatgatggccactggtaaactgctcctcagctattccccaccgggagctaaaccaccaaccaatcgcaaggatgcaatgctaggcacacacatcatctgggacctagggttacaatccagttgttccatggttgcaccgtggatctccaacacagtgtacagacggtgtgcacgtgatgacttcactgagggcggatttataacttgcttctatcaaactagaattgtggtacctgcttcaacccctaccagtatgttcatgttaggctttgttagtgcgtgtccagacttcagtgtcagactgcttagggacactccccatattagtcaatcgaaactaataggacgtacacaaggcattgaagacctcattgacacagcgataaagaatgccttaagagtgtcccaaccaccctcgacccagtcaactgaagcaactagtggagtgaatagccaggaggtgccagctctaactgctgtggaaacaggagcatctggtcaagcaatccccagtgatgtggtggaaactaggcacgtggtaaattacaaaaccaggtctgaatcgtgtcttgagtcattctttgggagagctgcgtgtgtcacaatcctatccttgaccaactcctccaagagcggagaggagaaaaagcatttcaacatatggaatattacatacaccgacactgtccagttacgcagaaaattagagtttttcacgtattccaggtttgatcttgaaatgacttttgtattcacagagaactatcctagtacagccagtggagaagtgcgaaaccaggtgtaccagatcatgtatattccaccaggggcaccccgcccatcatcctgggatgactacacatggcaatcctcttcaaacccttccatcttctacatgtatggaaatgcacctccacggatgtcaattccttacgtagggattgccaatgcctattcacacttctacgatggctttgcacgggtgccacttgagggtgagaacaccgatgctggcgacacgttttacggtttagtgtccataaatgattttggagttttagcagttagagcagtaaaccgcagtaatccacatacaatacacacatctgtgagagtgtacatgaaaccaaaacacattcggtgttggtgccccagacctcctcgagctgtattatacaggggagagggagtggacatgatatccagtgcaattctacctctgaccaaggtagactcaattaccacttttgggtttggtcatcagaacaaagcagtgtacgttgccggttacaagatttgcaactaccacctagcaaccccaagtgatcacttgaatgcaattagtatgttatgggacagggatttaatggtggtggaatctagagcccagggaactgataccatcgccagatgtagttgcaggtgtggagtttactattgtgaatctaggaggaagtactaccctgtcacttttactggcccaacgtttcgattcatggaagcaaacgactactatccagcaagataccagtctcacatgctgatagggtgcggatttgcagaacccggggactgcggtgggatactgaggtgcactcatggggtaattggtatcattactgcaggaggtgaaggggtagtagcctttgctgacattagagacctctgggtgtatgaagaggaggccatggaacagggaataacaagctacatcgaatctctcggcacagcctttggcgcagggttcacccacacaatcagtgagaaagtgactgaattgacaacaatggttaccagcactatcacagaaaaactactgaaaaacttggtgaaaatagtgtcggctctagtgattgttgtgagaaattatgaggacactaccacgatccttgcaacactagcactactcgggtgtgatatatctccttggcaatggttgaagaagaaggcatgtgacttactagagattccttatgtgatgcgccaaggtgatgggtggatgaagaaattcacagaggcgtgcaatgcagctaaaggcttagagtggattagcaacaaaatttccaagtttatagattggttgaagtgtaaaattatcccagacgctaaggacaaggtggaatttctcaccaagttgaaacagctagacatgttggaaaatcaaattgcaaccatccaccaatcttgccccagccaagaacaacaagagattcttttcaacaatgtgagatggctagcagtccagtcccgtcggtttgcaccattatacgctgtggaggcacgccgaattaacaaaatggagagcacaataaacaattatatacagttcaagagcaaacaccgtattgaaccagtatgtatgctcattcatgggtcaccagggacgggtaaatctatagctacttcattaataggtagagcaatagcagagaaggaaagcacatcagtctattcaatgccacctgacccatctcactttgatggctataaacaacaaggggtagtgattatggacgacctaaaccaaaaccccgatggtatggacatgaaactgttttgccaaatggtatcaacagtggagtttattcctccaatggcctcattagaggagaagggcattttgtttacatctgattatgtcctggcttctaccaactctcattcaattgtaccacccacagtggctcacagtgatgccttaaccagacgatttgcatttgatgtggaggtttacacgatgtctgaacattcagtcaaaggcaaactgaatatggccacggccactcaattgtgtaaggattgtccaacacctgcaaattttaaaaagtgttgccctctcgtttgtggaaaggccttgcaattaatggacaggtacaccagacaaaggttcactgtagatgagattaccacattaatcatgaatgagaaaaacagaagggccaatatcggcaattgcatggaagccttgtttcaaggaccattaaggtataaagatttgaagatcgatgtgaagacagttcccccccctgagtgcatcagtgatttgttacaagcagtggattctcaagaggttagggattactgtgagaagaaaggctggatcgttaacgttactagccagattcaactagaaaggaacatcaatagggccatgactatactccaagctgttaccacattcgcagcagtcgcaggagtagtgtatgtaatgtacaaactcttcgccggtcaacagggtgcatacactggcttgccaaacaaaaaacccaatgtccctactatcagagtcgctaaagtccaggggccaggatttgactacgcagtggcaatggcaaaaagaaacatagttactgcaaccaccaccaagggtgaatttaccatgctaggggtgcatgataatgtagcaatattgccaacccatgccgctccaggagaaaccattattattgatgggaaagaagtagagatcctagatgccagagccttagaagatcaagcgggaaccaatcttgagatcaccattattactctaaaaagaaatgagaagtttagagacatcagatcacatattcccacccaaattactgaaactaacgatggagtgttgatcgtgaacactagcaagtaccccaatatgtatgtccccgttggtgctgtgaccgaacagggatatcttaatctcagtggacgtcaaactgctcgcactttaatgtacaactttccaacaagggcaggccagtgcggaggaatcatcacttgtactggcaaagtcattgggatgcatgttggcgggaacggttcacatgggtttgcagcagccctcaagcgatcatacttcactcaaaatcagggcgaaatccagtggatgaggtcatcaaaagaagtggggtaccccattataaatgccccatccaagacaaagttagaacccagtgctttccactatgtttttgaaggtgttaaggaaccagctgtactcactaagaatgaccccagactaaaaacagattttgaagaagccatcttttctaaatatgtggggaacaaaattactgaagtggacgagtacatgaaagaagcagtggatcactatgcaggacagttaatgtcactggatatcaacacagaacagatgtgcctggaggatgccatgtacggcaccgatggtcttgaggccctggatcttagcactagtgctggatatccttatgttgcaatggggaaaaagaaaagagacattctaaataaacagaccagagatactaaggagatgcagagacttttagatacctatggaatcaatctaccattagtcacgtacgtgaaagatgaactcaggtcaaagactaaagtggaacaaggaaagtcaagattgattgaagcttccagccttaatgattcagttgcaatgagaatggcctttggcaatctttacgcagctttccacaagaatccaggtgtggtgacaggatcagcagttggttgtgacccagatttgttttggagtaagataccagtgctaatggaagaaaaactcttcgcttttgactacacagggtatgatgcctcactcagccctgcttggtttgaagctcttaaaatggtgttagaaaaaattggatttggcagtagagtagactatatagactacctgaaccactctcaccacctttacaaaaacaagacttattgtgtcaaaggcggcatgccatccggctgctctggcacctcaattttcaactcaatgattaacaacctgatcattaggacgcttttactgagaacctacaagggcatagacttggaccatttaaaaatgattgcctatggtgatgacgtgatagcttcctacccccatgaggttgacgctagtctcctagcccaatcaggaaaagactatggactaaccatgactccagcagataaatcagtaacctttgaaacagtcacatgggagaatgtaacatttctgaaaagatttttcagagcagatgagaagtatccattcctggtgcatccagtgatgccaatgaaagaaattcacgaatcaatcagatggaccaaggaccctagaaacacacaggatcacgtacgctcgttgtgcctattagcttggcacaacggtgaagaagaatacaataaatttttagctaaaatcagaagtgtgccaattggaagagctttattgctcccagagtactctacattgtaccgccgatggctcgactcattttagtaaccctacctcagtcggattggattgggttacactgttgtaggggtaaatttttctttaattcggagaaaaaaaaaaaaaaaaaaaaaaaaagatctacctgcaggCATGCAAGCTTGGCGTAATCATGGTCATAGCTGTTTCCTGTGTGAAATTGTTATCCGCTCACAATTCCACACAACATACGAGCCGGAAGCATAAAGTGTAAAGCCTGGGGTGCCTAATGAGTGAGCTAACTCACATTAATTGCGTTGCGCTCACTGCCCGCTTTCCAGTCGGGAAACCTGTCGTGCCAGCTGCATTAATGAATCGGCCAACGCGCGGGGAGAGGCGGTTTGCGTATTGGGCGCTCTTCCGCTTCCTCGCTCACTGACTCGCTGCGCTCGGTCGTTCGGCTGCGGCGAGCGGTATCAGCTCACTCAAAGGCGGTAATACGGTTATCCACAGAATCAGGGGATAACGCAGGAAAGAACATGTGAGCAAAAGGCCAGCAAAAGGCCAGGAACCGTAAAAAGGCCGCGTTGCTGGCGTTTTTCCATAGGCTCCGCCCCCCTGACGAGCATCACAAAAATCGACGCTCAAGTCAGAGGTGGCGAAACCCGACAGGACTATAAAGATACCAGGCGTTTCCCCCTGGAAGCTCCCTCGTGCGCTCTCCTGTTCCGACCCTGCCGCTTACCGGATACCTGTCCGCCTTTCTCCCTTCGGGAAGCGTGGCGCTTTCTCATAGCTCACGCTGTAGGTATCTCAGTTCGGTGTAGGTCGTTCGCTCCAAGCTGGGCTGTGTGCACGAACCCCCCGTTCAGCCCGACCGCTGCGCCTTATCCGGTAACTATCGTCTTGAGTCCAACCCGGTAAGACACGACTTATCGCCACTGGCAGCAGCCACTGGTAACAGGATTAGCAGAGCGAGGTATGTAGGCGGTGCTACAGAGTTCTTGAAGTGGTGGCCTAACTACGGCTACACTAGAAGAACAGTATTTGGTATCTGCGCTCTGCTGAAGCCAGTTACCTTCGGAAAAAGAGTTGGTAGCTCTTGATCCGGCAAACAAACCACCGCTGGTAGCGGTGGTTTTTTTGTTTGCAAGCAGCAGATTACGCGCAGAAAAAAAGGATCTCAAGAAGATCCTTTGATCTTTTCTACGGGGTCTGACGCTCAGTGGAACGAAAACTCACGTTAAGGGATTTTGGTCATGAGATTATCAAAAAGGATCTTCACCTAGATCCTTTTAAATTAAAAATGAAGTTTTAAATCAATCTAAAGTATATATGAGTAAACTTGGTCTGACAGTTACCAATGCTTAATCAGTGAGGCACCTATCTCAGCGATCTGTCTATTTCGTTCATCCATAGTTGCCTGACTCCCCGTCGTGTAGATAACTACGATACGGGAGGGCTTACCATCTGGCCCCAGTGCTGCAATGATACCGCGAGACCCACGCTCACCGGCTCCAGATTTATCAGCAATAAACCAGCCAGCCGGAAGGGCCGAGCGCAGAAGTGGTCCTGCAACTTTATCCGCCTCCATCCAGTCTATTAATTGTTGCCGGGAAGCTAGAGTAAGTAGTTCGCCAGTTAATAGTTTGCGCAACGTTGTTGCCATTGCTACAGGCATCGTGGTGTCACGCTCGTCGTTTGGTATGGCTTCATTCAGCTCCGGTTCCCAACGATCAAGGCGAGTTACATGATCCCCCATGTTGTGCAAAAAAGCGGTTAGCTCCTTCGGTCCTCCGATCGTTGTCAGAAGTAAGTTGGCCGCAGTGTTATCACTCATGGTTATGGCAGCACTGCATAATTCTCTTACTGTCATGCCATCCGTAAGATGCTTTTCTGTGACTGGTGAGTACTCAACCAAGTCATTCTGAGAATAGTGTATGCGGCGACCGAGTTGCTCTTGCCCGGCGTCAATACGGGATAATACCGCGCCACATAGCAGAACTTTAAAAGTGCTCATCATTGGAAAACGTTCTTCGGGGCGAAAACTCTCAAGGATCTTACCGCTGTTGAGATCCAGTTCGATGTAACCCACTCGTGCACCCAACTGATCTTCAGCATCTTTTACTTTCACCAGCGTTTCTGGGTGAGCAAAAACAGGAAGGCAAAATGCCGCAAAAAAGGGAATAAGGGCGACACGGAAATGTTGAATACTCATACTCTTCCTTTTTCAATATTATTGAAGCATTTATCAGGGTTATTGTCTCATGAGCGGATACATATTTGAATGTATTTAGAAAAATAAACAAATAGGGGTTCCGCGCACATTTCCCCGAAAAGTGCCACCTGACGTCTAAGAAACCATTATTATCATGACATTAACCTATAAAAATAGGCGTATCACGAGGCCCTTTCGTCTCGCGCGTTTCGGTGATGACGGTGAAAACCTCTGACACATGCAGCTCCCGGAGACGGTCACAGCTTGTCTGTAAGCGGATGCCGGGAGCAGACAAGCCCGTCAGGGCGCGTCAGCGGGTGTTGGCGGGTGTCGGGGCTGGCTTAACTATGCGGCATCAGAGCAGATTGTACTGAGAGTGCACCATATGCGGTGTGAAATACCGCACAGATGCGTAAGGAGAAAATACCGCATCAGGCGCCATTCGCCATTCAGGCTGCGCAACTGTTGGGAAGGGCGATCGGTGCGGGCCTCTTCGCTATTACGCCAGCTGGCGAAAGGGGGATGTGCTGCAAGGCGATTAAGTTGGGTAACGCCAGGGTTTTCCCAGTCACGACGTTGTAAAACGACGGCCAGTGAATTCGAGCTCGGTACCCGGGGATCCTCTAGA

1. rCVA21-GFP-129-TC:

gtcgactaatacgactcactatagggttaaaacagctctggggttgttcccaccccagaggcccacgtggcggctagtactctggtattacggtacctttgtacgcctgttttgtatcccttcccccgtaactttagaagcttatcaaaggttcaatagcaggggtacaaaccagtacctctacgaacaagcacttctgtttccccggtgatatcacatagactgtacccacggtcaaaagtgattgatccgttatccgcttgagtacttcgagaagcctagtatcaccttggaatcttcgatgcgttgcgctcaacactctgccccgagtgtagcttaggctgatgagtctgggcactccccaccggcgacggtggcccaggctgcgttggcggcctacccatggctgatgccgtgggacgctagttgtgaacaaggtgtgaagagcctattgagctactcaagagtcctccggcccctgaatgcggctaatcctaaccacggagcaaccgctcacaacccagtgagtaggttgtcgtaatgcgtaagtctgtggcggaaccgactactttgggtgtccgtgtttccctttatattcatactggctgcttatggtgacaatttacaaattgttaccatatagctattggattggccacccagtattgtgcaatatatttgagtgtttctttcataagccttattaacatcacatttttaatcacaataaacagtgcaaatgGTGAGCAAGGGCGAGGAGCTGTTCACCGGGGTGGTGCCCATCCTGGTCGAGCTGGACGGCGACGTAAACGGCCACAAGTTCAGCGTGTCCGGCGAGGGCGAGGGCGATGCCACCTACGGCAAGCTGACCCTGAAGTTCATCTGCACCACCGGCAAGCTGCCCGTGCCCTGGCCCACCCTCGTGACCACCCTGACCTACGGCGTGCAGTGCTTCAGCCGCTACCCCGACCACATGAAGCAGCACGACTTCTTCAAGTCCGCCATGCCCGAAGGCTACGTCCAGGAGCGCACCATCTTCTTCAAGGACGACGGCAACTACAAGACCCGCGCCGAGGTGAAGTTCGAGGGCGACACCCTGGTGAACCGCATCGAGCTGAAGGGCATCTTCCTGAACTGCTGtCCCGGCTGtTGCATGGAGCCCGCTTTGTTTCAAGGAGCTCAAgtttcaacgcaaaagaccggtgcgcacgagaatcaaaacgtggcagccaatggatccaccattaattacactactatcaactattacaaagacagtgcgagtaattccgctactagacaagacctctcccaagatccatcaaaattcacagaaccggttaaggacttaatgttgaaaacagcaccagctctaaactcgcctaacgtggaagcatgtgggtacagtgaccgtgtgaggcaaatcactttaggcaactcgactattactacacaagaagcagccaatgctattgttgcttacggtgaatggcccacttacataaatgattcagaagctaatccggtagatgcacccactgagccagatgttagtagcaaccggttttacaccctagaatcggtgtcttggaagaccacttcaaggggatggtggtggaagttaccagattgtttgaaggacatgggaatgtttggtcagaatatgtactatcactacttggggcgctctggttacaccattcatgtccagtgcaacgcttcaaaatttcaccaaggggcgttaggagtttttctgataccagagtttgtcatggcttgcaacactgagagtaaaacgtcatacgtttcatacatcaatgcaaatcctggtgagagaggcggtgagtttacgaacacctacaatccgttaaatacagacgccagtgagggcagaaagtttgcagcattggattatttgctgggttctggtgttctagcaggaaacgcctttgtgtacccgcaccagatcatcaacctacgtaccaacaacagtgcaacaattgtggtgccatacgtaaactcacttgtgattgattgtatggcaaaacacaataactggggcattgtcatattaccactggcacccttggcctttgccgcaacatcgtcaccacaggtgcctattacagtgaccattgcacccatgtgtacagaattcaatgggttgagaaacatcaccgtcccagtacatcaagggttgccgacaatgaacacacctggttccaatcaattccttacatctgatgacttccagtcgccctgtgccttacctaattttgatgttactccaccaatacacatacccggggaagtaaagaatatgatggaactagctgaaattgacacattgatcccaatgaacgcagtggacgggaaggtgaacacaatggagatgtatcaaataccattgaatgacaatttgagcaaggcacctatattctgtttatccctatcacctgcttctgataaacgactgagccgcaccatgttgggtgaaatcctaaattattacacccattggacggggtccatcaggttcacctttctattttgtggtagtatgatggccactggtaaactgctcctcagctattccccaccgggagctaaaccaccaaccaatcgcaaggatgcaatgctaggcacacacatcatctgggacctagggttacaatccagttgttccatggttgcaccgtggatctccaacacagtgtacagacggtgtgcacgtgatgacttcactgagggcggatttataacttgcttctatcaaactagaattgtggtacctgcttcaacccctaccagtatgttcatgttaggctttgttagtgcgtgtccagacttcagtgtcagactgcttagggacactccccatattagtcaatcgaaactaataggacgtacacaaggcattgaagacctcattgacacagcgataaagaatgccttaagagtgtcccaaccaccctcgacccagtcaactgaagcaactagtggagtgaatagccaggaggtgccagctctaactgctgtggaaacaggagcatctggtcaagcaatccccagtgatgtggtggaaactaggcacgtggtaaattacaaaaccaggtctgaatcgtgtcttgagtcattctttgggagagctgcgtgtgtcacaatcctatccttgaccaactcctccaagagcggagaggagaaaaagcatttcaacatatggaatattacatacaccgacactgtccagttacgcagaaaattagagtttttcacgtattccaggtttgatcttgaaatgacttttgtattcacagagaactatcctagtacagccagtggagaagtgcgaaaccaggtgtaccagatcatgtatattccaccaggggcaccccgcccatcatcctgggatgactacacatggcaatcctcttcaaacccttccatcttctacatgtatggaaatgcacctccacggatgtcaattccttacgtagggattgccaatgcctattcacacttctacgatggctttgcacgggtgccacttgagggtgagaacaccgatgctggcgacacgttttacggtttagtgtccataaatgattttggagttttagcagttagagcagtaaaccgcagtaatccacatacaatacacacatctgtgagagtgtacatgaaaccaaaacacattcggtgttggtgccccagacctcctcgagctgtattatacaggggagagggagtggacatgatatccagtgcaattctacctctgaccaaggtagactcaattaccacttttgggtttggtcatcagaacaaagcagtgtacgttgccggttacaagatttgcaactaccacctagcaaccccaagtgatcacttgaatgcaattagtatgttatgggacagggatttaatggtggtggaatctagagcccagggaactgataccatcgccagatgtagttgcaggtgtggagtttactattgtgaatctaggaggaagtactaccctgtcacttttactggcccaacgtttcgattcatggaagcaaacgactactatccagcaagataccagtctcacatgctgatagggtgcggatttgcagaacccggggactgcggtgggatactgaggtgcactcatggggtaattggtatcattactgcaggaggtgaaggggtagtagcctttgctgacattagagacctctgggtgtatgaagaggaggccatggaacagggaataacaagctacatcgaatctctcggcacagcctttggcgcagggttcacccacacaatcagtgagaaagtgactgaattgacaacaatggttaccagcactatcacagaaaaactactgaaaaacttggtgaaaatagtgtcggctctagtgattgttgtgagaaattatgaggacactaccacgatccttgcaacactagcactactcgggtgtgatatatctccttggcaatggttgaagaagaaggcatgtgacttactagagattccttatgtgatgcgccaaggtgatgggtggatgaagaaattcacagaggcgtgcaatgcagctaaaggcttagagtggattagcaacaaaatttccaagtttatagattggttgaagtgtaaaattatcccagacgctaaggacaaggtggaatttctcaccaagttgaaacagctagacatgttggaaaatcaaattgcaaccatccaccaatcttgccccagccaagaacaacaagagattcttttcaacaatgtgagatggctagcagtccagtcccgtcggtttgcaccattatacgctgtggaggcacgccgaattaacaaaatggagagcacaataaacaattatatacagttcaagagcaaacaccgtattgaaccagtatgtatgctcattcatgggtcaccagggacgggtaaatctatagctacttcattaataggtagagcaatagcagagaaggaaagcacatcagtctattcaatgccacctgacccatctcactttgatggctataaacaacaaggggtagtgattatggacgacctaaaccaaaaccccgatggtatggacatgaaactgttttgccaaatggtatcaacagtggagtttattcctccaatggcctcattagaggagaagggcattttgtttacatctgattatgtcctggcttctaccaactctcattcaattgtaccacccacagtggctcacagtgatgccttaaccagacgatttgcatttgatgtggaggtttacacgatgtctgaacattcagtcaaaggcaaactgaatatggccacggccactcaattgtgtaaggattgtccaacacctgcaaattttaaaaagtgttgccctctcgtttgtggaaaggccttgcaattaatggacaggtacaccagacaaaggttcactgtagatgagattaccacattaatcatgaatgagaaaaacagaagggccaatatcggcaattgcatggaagccttgtttcaaggaccattaaggtataaagatttgaagatcgatgtgaagacagttcccccccctgagtgcatcagtgatttgttacaagcagtggattctcaagaggttagggattactgtgagaagaaaggctggatcgttaacgttactagccagattcaactagaaaggaacatcaatagggccatgactatactccaagctgttaccacattcgcagcagtcgcaggagtagtgtatgtaatgtacaaactcttcgccggtcaacagggtgcatacactggcttgccaaacaaaaaacccaatgtccctactatcagagtcgctaaagtccaggggccaggatttgactacgcagtggcaatggcaaaaagaaacatagttactgcaaccaccaccaagggtgaatttaccatgctaggggtgcatgataatgtagcaatattgccaacccatgccgctccaggagaaaccattattattgatgggaaagaagtagagatcctagatgccagagccttagaagatcaagcgggaaccaatcttgagatcaccattattactctaaaaagaaatgagaagtttagagacatcagatcacatattcccacccaaattactgaaactaacgatggagtgttgatcgtgaacactagcaagtaccccaatatgtatgtccccgttggtgctgtgaccgaacagggatatcttaatctcagtggacgtcaaactgctcgcactttaatgtacaactttccaacaagggcaggccagtgcggaggaatcatcacttgtactggcaaagtcattgggatgcatgttggcgggaacggttcacatgggtttgcagcagccctcaagcgatcatacttcactcaaaatcagggcgaaatccagtggatgaggtcatcaaaagaagtggggtaccccattataaatgccccatccaagacaaagttagaacccagtgctttccactatgtttttgaaggtgttaaggaaccagctgtactcactaagaatgaccccagactaaaaacagattttgaagaagccatcttttctaaatatgtggggaacaaaattactgaagtggacgagtacatgaaagaagcagtggatcactatgcaggacagttaatgtcactggatatcaacacagaacagatgtgcctggaggatgccatgtacggcaccgatggtcttgaggccctggatcttagcactagtgctggatatccttatgttgcaatggggaaaaagaaaagagacattctaaataaacagaccagagatactaaggagatgcagagacttttagatacctatggaatcaatctaccattagtcacgtacgtgaaagatgaactcaggtcaaagactaaagtggaacaaggaaagtcaagattgattgaagcttccagccttaatgattcagttgcaatgagaatggcctttggcaatctttacgcagctttccacaagaatccaggtgtggtgacaggatcagcagttggttgtgacccagatttgttttggagtaagataccagtgctaatggaagaaaaactcttcgcttttgactacacagggtatgatgcctcactcagccctgcttggtttgaagctcttaaaatggtgttagaaaaaattggatttggcagtagagtagactatatagactacctgaaccactctcaccacctttacaaaaacaagacttattgtgtcaaaggcggcatgccatccggctgctctggcacctcaattttcaactcaatgattaacaacctgatcattaggacgcttttactgagaacctacaagggcatagacttggaccatttaaaaatgattgcctatggtgatgacgtgatagcttcctacccccatgaggttgacgctagtctcctagcccaatcaggaaaagactatggactaaccatgactccagcagataaatcagtaacctttgaaacagtcacatgggagaatgtaacatttctgaaaagatttttcagagcagatgagaagtatccattcctggtgcatccagtgatgccaatgaaagaaattcacgaatcaatcagatggaccaaggaccctagaaacacacaggatcacgtacgctcgttgtgcctattagcttggcacaacggtgaagaagaatacaataaatttttagctaaaatcagaagtgtgccaattggaagagctttattgctcccagagtactctacattgtaccgccgatggctcgactcattttagtaaccctacctcagtcggattggattgggttacactgttgtaggggtaaatttttctttaattcggagaaaaaaaaaaaaaaaaaaaaaaaaagatctacctgcaggCATGCAAGCTTGGCGTAATCATGGTCATAGCTGTTTCCTGTGTGAAATTGTTATCCGCTCACAATTCCACACAACATACGAGCCGGAAGCATAAAGTGTAAAGCCTGGGGTGCCTAATGAGTGAGCTAACTCACATTAATTGCGTTGCGCTCACTGCCCGCTTTCCAGTCGGGAAACCTGTCGTGCCAGCTGCATTAATGAATCGGCCAACGCGCGGGGAGAGGCGGTTTGCGTATTGGGCGCTCTTCCGCTTCCTCGCTCACTGACTCGCTGCGCTCGGTCGTTCGGCTGCGGCGAGCGGTATCAGCTCACTCAAAGGCGGTAATACGGTTATCCACAGAATCAGGGGATAACGCAGGAAAGAACATGTGAGCAAAAGGCCAGCAAAAGGCCAGGAACCGTAAAAAGGCCGCGTTGCTGGCGTTTTTCCATAGGCTCCGCCCCCCTGACGAGCATCACAAAAATCGACGCTCAAGTCAGAGGTGGCGAAACCCGACAGGACTATAAAGATACCAGGCGTTTCCCCCTGGAAGCTCCCTCGTGCGCTCTCCTGTTCCGACCCTGCCGCTTACCGGATACCTGTCCGCCTTTCTCCCTTCGGGAAGCGTGGCGCTTTCTCATAGCTCACGCTGTAGGTATCTCAGTTCGGTGTAGGTCGTTCGCTCCAAGCTGGGCTGTGTGCACGAACCCCCCGTTCAGCCCGACCGCTGCGCCTTATCCGGTAACTATCGTCTTGAGTCCAACCCGGTAAGACACGACTTATCGCCACTGGCAGCAGCCACTGGTAACAGGATTAGCAGAGCGAGGTATGTAGGCGGTGCTACAGAGTTCTTGAAGTGGTGGCCTAACTACGGCTACACTAGAAGAACAGTATTTGGTATCTGCGCTCTGCTGAAGCCAGTTACCTTCGGAAAAAGAGTTGGTAGCTCTTGATCCGGCAAACAAACCACCGCTGGTAGCGGTGGTTTTTTTGTTTGCAAGCAGCAGATTACGCGCAGAAAAAAAGGATCTCAAGAAGATCCTTTGATCTTTTCTACGGGGTCTGACGCTCAGTGGAACGAAAACTCACGTTAAGGGATTTTGGTCATGAGATTATCAAAAAGGATCTTCACCTAGATCCTTTTAAATTAAAAATGAAGTTTTAAATCAATCTAAAGTATATATGAGTAAACTTGGTCTGACAGTTACCAATGCTTAATCAGTGAGGCACCTATCTCAGCGATCTGTCTATTTCGTTCATCCATAGTTGCCTGACTCCCCGTCGTGTAGATAACTACGATACGGGAGGGCTTACCATCTGGCCCCAGTGCTGCAATGATACCGCGAGACCCACGCTCACCGGCTCCAGATTTATCAGCAATAAACCAGCCAGCCGGAAGGGCCGAGCGCAGAAGTGGTCCTGCAACTTTATCCGCCTCCATCCAGTCTATTAATTGTTGCCGGGAAGCTAGAGTAAGTAGTTCGCCAGTTAATAGTTTGCGCAACGTTGTTGCCATTGCTACAGGCATCGTGGTGTCACGCTCGTCGTTTGGTATGGCTTCATTCAGCTCCGGTTCCCAACGATCAAGGCGAGTTACATGATCCCCCATGTTGTGCAAAAAAGCGGTTAGCTCCTTCGGTCCTCCGATCGTTGTCAGAAGTAAGTTGGCCGCAGTGTTATCACTCATGGTTATGGCAGCACTGCATAATTCTCTTACTGTCATGCCATCCGTAAGATGCTTTTCTGTGACTGGTGAGTACTCAACCAAGTCATTCTGAGAATAGTGTATGCGGCGACCGAGTTGCTCTTGCCCGGCGTCAATACGGGATAATACCGCGCCACATAGCAGAACTTTAAAAGTGCTCATCATTGGAAAACGTTCTTCGGGGCGAAAACTCTCAAGGATCTTACCGCTGTTGAGATCCAGTTCGATGTAACCCACTCGTGCACCCAACTGATCTTCAGCATCTTTTACTTTCACCAGCGTTTCTGGGTGAGCAAAAACAGGAAGGCAAAATGCCGCAAAAAAGGGAATAAGGGCGACACGGAAATGTTGAATACTCATACTCTTCCTTTTTCAATATTATTGAAGCATTTATCAGGGTTATTGTCTCATGAGCGGATACATATTTGAATGTATTTAGAAAAATAAACAAATAGGGGTTCCGCGCACATTTCCCCGAAAAGTGCCACCTGACGTCTAAGAAACCATTATTATCATGACATTAACCTATAAAAATAGGCGTATCACGAGGCCCTTTCGTCTCGCGCGTTTCGGTGATGACGGTGAAAACCTCTGACACATGCAGCTCCCGGAGACGGTCACAGCTTGTCTGTAAGCGGATGCCGGGAGCAGACAAGCCCGTCAGGGCGCGTCAGCGGGTGTTGGCGGGTGTCGGGGCTGGCTTAACTATGCGGCATCAGAGCAGATTGTACTGAGAGTGCACCATATGCGGTGTGAAATACCGCACAGATGCGTAAGGAGAAAATACCGCATCAGGCGCCATTCGCCATTCAGGCTGCGCAACTGTTGGGAAGGGCGATCGGTGCGGGCCTCTTCGCTATTACGCCAGCTGGCGAAAGGGGGATGTGCTGCAAGGCGATTAAGTTGGGTAACGCCAGGGTTTTCCCAGTCACGACGTTGTAAAACGACGGCCAGTGAATTCGAGCTCGGTACCCGGGGATCCTCTAGA

1. rCVA21-GFP-154-TC:

gtcgactaatacgactcactatagggttaaaacagctctggggttgttcccaccccagaggcccacgtggcggctagtactctggtattacggtacctttgtacgcctgttttgtatcccttcccccgtaactttagaagcttatcaaaggttcaatagcaggggtacaaaccagtacctctacgaacaagcacttctgtttccccggtgatatcacatagactgtacccacggtcaaaagtgattgatccgttatccgcttgagtacttcgagaagcctagtatcaccttggaatcttcgatgcgttgcgctcaacactctgccccgagtgtagcttaggctgatgagtctgggcactccccaccggcgacggtggcccaggctgcgttggcggcctacccatggctgatgccgtgggacgctagttgtgaacaaggtgtgaagagcctattgagctactcaagagtcctccggcccctgaatgcggctaatcctaaccacggagcaaccgctcacaacccagtgagtaggttgtcgtaatgcgtaagtctgtggcggaaccgactactttgggtgtccgtgtttccctttatattcatactggctgcttatggtgacaatttacaaattgttaccatatagctattggattggccacccagtattgtgcaatatatttgagtgtttctttcataagccttattaacatcacatttttaatcacaataaacagtgcaaatgGTGAGCAAGGGCGAGGAGCTGTTCACCGGGGTGGTGCCCATCCTGGTCGAGCTGGACGGCGACGTAAACGGCCACAAGTTCAGCGTGTCCGGCGAGGGCGAGGGCGATGCCACCTACGGCAAGCTGACCCTGAAGTTCATCTGCACCACCGGCAAGCTGCCCGTGCCCTGGCCCACCCTCGTGACCACCCTGACCTACGGCGTGCAGTGCTTCAGCCGCTACCCCGACCACATGAAGCAGCACGACTTCTTCAAGTCCGCCATGCCCGAAGGCTACGTCCAGGAGCGCACCATCTTCTTCAAGGACGACGGCAACTACAAGACCCGCGCCGAGGTGAAGTTCGAGGGCGACACCCTGGTGAACCGCATCGAGCTGAAGGGCATCGACTTCAAGGAGGACGGCAACATCCTGGGGCACAAGCTGGAGTACAACTACAACAGCCACAACGTCTATATCATGTTCCTGAACTGCTGtCCCGGCTGtTGCATGGAGCCCGCTTTGTTTCAAGGAGCTCAAgtttcaacgcaaaagaccggtgcgcacgagaatcaaaacgtggcagccaatggatccaccattaattacactactatcaactattacaaagacagtgcgagtaattccgctactagacaagacctctcccaagatccatcaaaattcacagaaccggttaaggacttaatgttgaaaacagcaccagctctaaactcgcctaacgtggaagcatgtgggtacagtgaccgtgtgaggcaaatcactttaggcaactcgactattactacacaagaagcagccaatgctattgttgcttacggtgaatggcccacttacataaatgattcagaagctaatccggtagatgcacccactgagccagatgttagtagcaaccggttttacaccctagaatcggtgtcttggaagaccacttcaaggggatggtggtggaagttaccagattgtttgaaggacatgggaatgtttggtcagaatatgtactatcactacttggggcgctctggttacaccattcatgtccagtgcaacgcttcaaaatttcaccaaggggcgttaggagtttttctgataccagagtttgtcatggcttgcaacactgagagtaaaacgtcatacgtttcatacatcaatgcaaatcctggtgagagaggcggtgagtttacgaacacctacaatccgttaaatacagacgccagtgagggcagaaagtttgcagcattggattatttgctgggttctggtgttctagcaggaaacgcctttgtgtacccgcaccagatcatcaacctacgtaccaacaacagtgcaacaattgtggtgccatacgtaaactcacttgtgattgattgtatggcaaaacacaataactggggcattgtcatattaccactggcacccttggcctttgccgcaacatcgtcaccacaggtgcctattacagtgaccattgcacccatgtgtacagaattcaatgggttgagaaacatcaccgtcccagtacatcaagggttgccgacaatgaacacacctggttccaatcaattccttacatctgatgacttccagtcgccctgtgccttacctaattttgatgttactccaccaatacacatacccggggaagtaaagaatatgatggaactagctgaaattgacacattgatcccaatgaacgcagtggacgggaaggtgaacacaatggagatgtatcaaataccattgaatgacaatttgagcaaggcacctatattctgtttatccctatcacctgcttctgataaacgactgagccgcaccatgttgggtgaaatcctaaattattacacccattggacggggtccatcaggttcacctttctattttgtggtagtatgatggccactggtaaactgctcctcagctattccccaccgggagctaaaccaccaaccaatcgcaaggatgcaatgctaggcacacacatcatctgggacctagggttacaatccagttgttccatggttgcaccgtggatctccaacacagtgtacagacggtgtgcacgtgatgacttcactgagggcggatttataacttgcttctatcaaactagaattgtggtacctgcttcaacccctaccagtatgttcatgttaggctttgttagtgcgtgtccagacttcagtgtcagactgcttagggacactccccatattagtcaatcgaaactaataggacgtacacaaggcattgaagacctcattgacacagcgataaagaatgccttaagagtgtcccaaccaccctcgacccagtcaactgaagcaactagtggagtgaatagccaggaggtgccagctctaactgctgtggaaacaggagcatctggtcaagcaatccccagtgatgtggtggaaactaggcacgtggtaaattacaaaaccaggtctgaatcgtgtcttgagtcattctttgggagagctgcgtgtgtcacaatcctatccttgaccaactcctccaagagcggagaggagaaaaagcatttcaacatatggaatattacatacaccgacactgtccagttacgcagaaaattagagtttttcacgtattccaggtttgatcttgaaatgacttttgtattcacagagaactatcctagtacagccagtggagaagtgcgaaaccaggtgtaccagatcatgtatattccaccaggggcaccccgcccatcatcctgggatgactacacatggcaatcctcttcaaacccttccatcttctacatgtatggaaatgcacctccacggatgtcaattccttacgtagggattgccaatgcctattcacacttctacgatggctttgcacgggtgccacttgagggtgagaacaccgatgctggcgacacgttttacggtttagtgtccataaatgattttggagttttagcagttagagcagtaaaccgcagtaatccacatacaatacacacatctgtgagagtgtacatgaaaccaaaacacattcggtgttggtgccccagacctcctcgagctgtattatacaggggagagggagtggacatgatatccagtgcaattctacctctgaccaaggtagactcaattaccacttttgggtttggtcatcagaacaaagcagtgtacgttgccggttacaagatttgcaactaccacctagcaaccccaagtgatcacttgaatgcaattagtatgttatgggacagggatttaatggtggtggaatctagagcccagggaactgataccatcgccagatgtagttgcaggtgtggagtttactattgtgaatctaggaggaagtactaccctgtcacttttactggcccaacgtttcgattcatggaagcaaacgactactatccagcaagataccagtctcacatgctgatagggtgcggatttgcagaacccggggactgcggtgggatactgaggtgcactcatggggtaattggtatcattactgcaggaggtgaaggggtagtagcctttgctgacattagagacctctgggtgtatgaagaggaggccatggaacagggaataacaagctacatcgaatctctcggcacagcctttggcgcagggttcacccacacaatcagtgagaaagtgactgaattgacaacaatggttaccagcactatcacagaaaaactactgaaaaacttggtgaaaatagtgtcggctctagtgattgttgtgagaaattatgaggacactaccacgatccttgcaacactagcactactcgggtgtgatatatctccttggcaatggttgaagaagaaggcatgtgacttactagagattccttatgtgatgcgccaaggtgatgggtggatgaagaaattcacagaggcgtgcaatgcagctaaaggcttagagtggattagcaacaaaatttccaagtttatagattggttgaagtgtaaaattatcccagacgctaaggacaaggtggaatttctcaccaagttgaaacagctagacatgttggaaaatcaaattgcaaccatccaccaatcttgccccagccaagaacaacaagagattcttttcaacaatgtgagatggctagcagtccagtcccgtcggtttgcaccattatacgctgtggaggcacgccgaattaacaaaatggagagcacaataaacaattatatacagttcaagagcaaacaccgtattgaaccagtatgtatgctcattcatgggtcaccagggacgggtaaatctatagctacttcattaataggtagagcaatagcagagaaggaaagcacatcagtctattcaatgccacctgacccatctcactttgatggctataaacaacaaggggtagtgattatggacgacctaaaccaaaaccccgatggtatggacatgaaactgttttgccaaatggtatcaacagtggagtttattcctccaatggcctcattagaggagaagggcattttgtttacatctgattatgtcctggcttctaccaactctcattcaattgtaccacccacagtggctcacagtgatgccttaaccagacgatttgcatttgatgtggaggtttacacgatgtctgaacattcagtcaaaggcaaactgaatatggccacggccactcaattgtgtaaggattgtccaacacctgcaaattttaaaaagtgttgccctctcgtttgtggaaaggccttgcaattaatggacaggtacaccagacaaaggttcactgtagatgagattaccacattaatcatgaatgagaaaaacagaagggccaatatcggcaattgcatggaagccttgtttcaaggaccattaaggtataaagatttgaagatcgatgtgaagacagttcccccccctgagtgcatcagtgatttgttacaagcagtggattctcaagaggttagggattactgtgagaagaaaggctggatcgttaacgttactagccagattcaactagaaaggaacatcaatagggccatgactatactccaagctgttaccacattcgcagcagtcgcaggagtagtgtatgtaatgtacaaactcttcgccggtcaacagggtgcatacactggcttgccaaacaaaaaacccaatgtccctactatcagagtcgctaaagtccaggggccaggatttgactacgcagtggcaatggcaaaaagaaacatagttactgcaaccaccaccaagggtgaatttaccatgctaggggtgcatgataatgtagcaatattgccaacccatgccgctccaggagaaaccattattattgatgggaaagaagtagagatcctagatgccagagccttagaagatcaagcgggaaccaatcttgagatcaccattattactctaaaaagaaatgagaagtttagagacatcagatcacatattcccacccaaattactgaaactaacgatggagtgttgatcgtgaacactagcaagtaccccaatatgtatgtccccgttggtgctgtgaccgaacagggatatcttaatctcagtggacgtcaaactgctcgcactttaatgtacaactttccaacaagggcaggccagtgcggaggaatcatcacttgtactggcaaagtcattgggatgcatgttggcgggaacggttcacatgggtttgcagcagccctcaagcgatcatacttcactcaaaatcagggcgaaatccagtggatgaggtcatcaaaagaagtggggtaccccattataaatgccccatccaagacaaagttagaacccagtgctttccactatgtttttgaaggtgttaaggaaccagctgtactcactaagaatgaccccagactaaaaacagattttgaagaagccatcttttctaaatatgtggggaacaaaattactgaagtggacgagtacatgaaagaagcagtggatcactatgcaggacagttaatgtcactggatatcaacacagaacagatgtgcctggaggatgccatgtacggcaccgatggtcttgaggccctggatcttagcactagtgctggatatccttatgttgcaatggggaaaaagaaaagagacattctaaataaacagaccagagatactaaggagatgcagagacttttagatacctatggaatcaatctaccattagtcacgtacgtgaaagatgaactcaggtcaaagactaaagtggaacaaggaaagtcaagattgattgaagcttccagccttaatgattcagttgcaatgagaatggcctttggcaatctttacgcagctttccacaagaatccaggtgtggtgacaggatcagcagttggttgtgacccagatttgttttggagtaagataccagtgctaatggaagaaaaactcttcgcttttgactacacagggtatgatgcctcactcagccctgcttggtttgaagctcttaaaatggtgttagaaaaaattggatttggcagtagagtagactatatagactacctgaaccactctcaccacctttacaaaaacaagacttattgtgtcaaaggcggcatgccatccggctgctctggcacctcaattttcaactcaatgattaacaacctgatcattaggacgcttttactgagaacctacaagggcatagacttggaccatttaaaaatgattgcctatggtgatgacgtgatagcttcctacccccatgaggttgacgctagtctcctagcccaatcaggaaaagactatggactaaccatgactccagcagataaatcagtaacctttgaaacagtcacatgggagaatgtaacatttctgaaaagatttttcagagcagatgagaagtatccattcctggtgcatccagtgatgccaatgaaagaaattcacgaatcaatcagatggaccaaggaccctagaaacacacaggatcacgtacgctcgttgtgcctattagcttggcacaacggtgaagaagaatacaataaatttttagctaaaatcagaagtgtgccaattggaagagctttattgctcccagagtactctacattgtaccgccgatggctcgactcattttagtaaccctacctcagtcggattggattgggttacactgttgtaggggtaaatttttctttaattcggagaaaaaaaaaaaaaaaaaaaaaaaaagatctacctgcaggCATGCAAGCTTGGCGTAATCATGGTCATAGCTGTTTCCTGTGTGAAATTGTTATCCGCTCACAATTCCACACAACATACGAGCCGGAAGCATAAAGTGTAAAGCCTGGGGTGCCTAATGAGTGAGCTAACTCACATTAATTGCGTTGCGCTCACTGCCCGCTTTCCAGTCGGGAAACCTGTCGTGCCAGCTGCATTAATGAATCGGCCAACGCGCGGGGAGAGGCGGTTTGCGTATTGGGCGCTCTTCCGCTTCCTCGCTCACTGACTCGCTGCGCTCGGTCGTTCGGCTGCGGCGAGCGGTATCAGCTCACTCAAAGGCGGTAATACGGTTATCCACAGAATCAGGGGATAACGCAGGAAAGAACATGTGAGCAAAAGGCCAGCAAAAGGCCAGGAACCGTAAAAAGGCCGCGTTGCTGGCGTTTTTCCATAGGCTCCGCCCCCCTGACGAGCATCACAAAAATCGACGCTCAAGTCAGAGGTGGCGAAACCCGACAGGACTATAAAGATACCAGGCGTTTCCCCCTGGAAGCTCCCTCGTGCGCTCTCCTGTTCCGACCCTGCCGCTTACCGGATACCTGTCCGCCTTTCTCCCTTCGGGAAGCGTGGCGCTTTCTCATAGCTCACGCTGTAGGTATCTCAGTTCGGTGTAGGTCGTTCGCTCCAAGCTGGGCTGTGTGCACGAACCCCCCGTTCAGCCCGACCGCTGCGCCTTATCCGGTAACTATCGTCTTGAGTCCAACCCGGTAAGACACGACTTATCGCCACTGGCAGCAGCCACTGGTAACAGGATTAGCAGAGCGAGGTATGTAGGCGGTGCTACAGAGTTCTTGAAGTGGTGGCCTAACTACGGCTACACTAGAAGAACAGTATTTGGTATCTGCGCTCTGCTGAAGCCAGTTACCTTCGGAAAAAGAGTTGGTAGCTCTTGATCCGGCAAACAAACCACCGCTGGTAGCGGTGGTTTTTTTGTTTGCAAGCAGCAGATTACGCGCAGAAAAAAAGGATCTCAAGAAGATCCTTTGATCTTTTCTACGGGGTCTGACGCTCAGTGGAACGAAAACTCACGTTAAGGGATTTTGGTCATGAGATTATCAAAAAGGATCTTCACCTAGATCCTTTTAAATTAAAAATGAAGTTTTAAATCAATCTAAAGTATATATGAGTAAACTTGGTCTGACAGTTACCAATGCTTAATCAGTGAGGCACCTATCTCAGCGATCTGTCTATTTCGTTCATCCATAGTTGCCTGACTCCCCGTCGTGTAGATAACTACGATACGGGAGGGCTTACCATCTGGCCCCAGTGCTGCAATGATACCGCGAGACCCACGCTCACCGGCTCCAGATTTATCAGCAATAAACCAGCCAGCCGGAAGGGCCGAGCGCAGAAGTGGTCCTGCAACTTTATCCGCCTCCATCCAGTCTATTAATTGTTGCCGGGAAGCTAGAGTAAGTAGTTCGCCAGTTAATAGTTTGCGCAACGTTGTTGCCATTGCTACAGGCATCGTGGTGTCACGCTCGTCGTTTGGTATGGCTTCATTCAGCTCCGGTTCCCAACGATCAAGGCGAGTTACATGATCCCCCATGTTGTGCAAAAAAGCGGTTAGCTCCTTCGGTCCTCCGATCGTTGTCAGAAGTAAGTTGGCCGCAGTGTTATCACTCATGGTTATGGCAGCACTGCATAATTCTCTTACTGTCATGCCATCCGTAAGATGCTTTTCTGTGACTGGTGAGTACTCAACCAAGTCATTCTGAGAATAGTGTATGCGGCGACCGAGTTGCTCTTGCCCGGCGTCAATACGGGATAATACCGCGCCACATAGCAGAACTTTAAAAGTGCTCATCATTGGAAAACGTTCTTCGGGGCGAAAACTCTCAAGGATCTTACCGCTGTTGAGATCCAGTTCGATGTAACCCACTCGTGCACCCAACTGATCTTCAGCATCTTTTACTTTCACCAGCGTTTCTGGGTGAGCAAAAACAGGAAGGCAAAATGCCGCAAAAAAGGGAATAAGGGCGACACGGAAATGTTGAATACTCATACTCTTCCTTTTTCAATATTATTGAAGCATTTATCAGGGTTATTGTCTCATGAGCGGATACATATTTGAATGTATTTAGAAAAATAAACAAATAGGGGTTCCGCGCACATTTCCCCGAAAAGTGCCACCTGACGTCTAAGAAACCATTATTATCATGACATTAACCTATAAAAATAGGCGTATCACGAGGCCCTTTCGTCTCGCGCGTTTCGGTGATGACGGTGAAAACCTCTGACACATGCAGCTCCCGGAGACGGTCACAGCTTGTCTGTAAGCGGATGCCGGGAGCAGACAAGCCCGTCAGGGCGCGTCAGCGGGTGTTGGCGGGTGTCGGGGCTGGCTTAACTATGCGGCATCAGAGCAGATTGTACTGAGAGTGCACCATATGCGGTGTGAAATACCGCACAGATGCGTAAGGAGAAAATACCGCATCAGGCGCCATTCGCCATTCAGGCTGCGCAACTGTTGGGAAGGGCGATCGGTGCGGGCCTCTTCGCTATTACGCCAGCTGGCGAAAGGGGGATGTGCTGCAAGGCGATTAAGTTGGGTAACGCCAGGGTTTTCCCAGTCACGACGTTGTAAAACGACGGCCAGTGAATTCGAGCTCGGTACCCGGGGATCCTCTAGA

1. rCVA21-GFP-172-TC:

gtcgactaatacgactcactatagggttaaaacagctctggggttgttcccaccccagaggcccacgtggcggctagtactctggtattacggtacctttgtacgcctgttttgtatcccttcccccgtaactttagaagcttatcaaaggttcaatagcaggggtacaaaccagtacctctacgaacaagcacttctgtttccccggtgatatcacatagactgtacccacggtcaaaagtgattgatccgttatccgcttgagtacttcgagaagcctagtatcaccttggaatcttcgatgcgttgcgctcaacactctgccccgagtgtagcttaggctgatgagtctgggcactccccaccggcgacggtggcccaggctgcgttggcggcctacccatggctgatgccgtgggacgctagttgtgaacaaggtgtgaagagcctattgagctactcaagagtcctccggcccctgaatgcggctaatcctaaccacggagcaaccgctcacaacccagtgagtaggttgtcgtaatgcgtaagtctgtggcggaaccgactactttgggtgtccgtgtttccctttatattcatactggctgcttatggtgacaatttacaaattgttaccatatagctattggattggccacccagtattgtgcaatatatttgagtgtttctttcataagccttattaacatcacatttttaatcacaataaacagtgcaaatgGTGAGCAAGGGCGAGGAGCTGTTCACCGGGGTGGTGCCCATCCTGGTCGAGCTGGACGGCGACGTAAACGGCCACAAGTTCAGCGTGTCCGGCGAGGGCGAGGGCGATGCCACCTACGGCAAGCTGACCCTGAAGTTCATCTGCACCACCGGCAAGCTGCCCGTGCCCTGGCCCACCCTCGTGACCACCCTGACCTACGGCGTGCAGTGCTTCAGCCGCTACCCCGACCACATGAAGCAGCACGACTTCTTCAAGTCCGCCATGCCCGAAGGCTACGTCCAGGAGCGCACCATCTTCTTCAAGGACGACGGCAACTACAAGACCCGCGCCGAGGTGAAGTTCGAGGGCGACACCCTGGTGAACCGCATCGAGCTGAAGGGCATCGACTTCAAGGAGGACGGCAACATCCTGGGGCACAAGCTGGAGTACAACTACAACAGCCACAACGTCTATATCATGGCCGACAAGCAGAAGAACGGCATCAAGGTGAACTTCAAGATCCGCCACAACATCTTCCTGAACTGCTGtCCCGGCTGtTGCATGGAGCCCGCTTTGTTTCAAGGAGCTCAAgtttcaacgcaaaagaccggtgcgcacgagaatcaaaacgtggcagccaatggatccaccattaattacactactatcaactattacaaagacagtgcgagtaattccgctactagacaagacctctcccaagatccatcaaaattcacagaaccggttaaggacttaatgttgaaaacagcaccagctctaaactcgcctaacgtggaagcatgtgggtacagtgaccgtgtgaggcaaatcactttaggcaactcgactattactacacaagaagcagccaatgctattgttgcttacggtgaatggcccacttacataaatgattcagaagctaatccggtagatgcacccactgagccagatgttagtagcaaccggttttacaccctagaatcggtgtcttggaagaccacttcaaggggatggtggtggaagttaccagattgtttgaaggacatgggaatgtttggtcagaatatgtactatcactacttggggcgctctggttacaccattcatgtccagtgcaacgcttcaaaatttcaccaaggggcgttaggagtttttctgataccagagtttgtcatggcttgcaacactgagagtaaaacgtcatacgtttcatacatcaatgcaaatcctggtgagagaggcggtgagtttacgaacacctacaatccgttaaatacagacgccagtgagggcagaaagtttgcagcattggattatttgctgggttctggtgttctagcaggaaacgcctttgtgtacccgcaccagatcatcaacctacgtaccaacaacagtgcaacaattgtggtgccatacgtaaactcacttgtgattgattgtatggcaaaacacaataactggggcattgtcatattaccactggcacccttggcctttgccgcaacatcgtcaccacaggtgcctattacagtgaccattgcacccatgtgtacagaattcaatgggttgagaaacatcaccgtcccagtacatcaagggttgccgacaatgaacacacctggttccaatcaattccttacatctgatgacttccagtcgccctgtgccttacctaattttgatgttactccaccaatacacatacccggggaagtaaagaatatgatggaactagctgaaattgacacattgatcccaatgaacgcagtggacgggaaggtgaacacaatggagatgtatcaaataccattgaatgacaatttgagcaaggcacctatattctgtttatccctatcacctgcttctgataaacgactgagccgcaccatgttgggtgaaatcctaaattattacacccattggacggggtccatcaggttcacctttctattttgtggtagtatgatggccactggtaaactgctcctcagctattccccaccgggagctaaaccaccaaccaatcgcaaggatgcaatgctaggcacacacatcatctgggacctagggttacaatccagttgttccatggttgcaccgtggatctccaacacagtgtacagacggtgtgcacgtgatgacttcactgagggcggatttataacttgcttctatcaaactagaattgtggtacctgcttcaacccctaccagtatgttcatgttaggctttgttagtgcgtgtccagacttcagtgtcagactgcttagggacactccccatattagtcaatcgaaactaataggacgtacacaaggcattgaagacctcattgacacagcgataaagaatgccttaagagtgtcccaaccaccctcgacccagtcaactgaagcaactagtggagtgaatagccaggaggtgccagctctaactgctgtggaaacaggagcatctggtcaagcaatccccagtgatgtggtggaaactaggcacgtggtaaattacaaaaccaggtctgaatcgtgtcttgagtcattctttgggagagctgcgtgtgtcacaatcctatccttgaccaactcctccaagagcggagaggagaaaaagcatttcaacatatggaatattacatacaccgacactgtccagttacgcagaaaattagagtttttcacgtattccaggtttgatcttgaaatgacttttgtattcacagagaactatcctagtacagccagtggagaagtgcgaaaccaggtgtaccagatcatgtatattccaccaggggcaccccgcccatcatcctgggatgactacacatggcaatcctcttcaaacccttccatcttctacatgtatggaaatgcacctccacggatgtcaattccttacgtagggattgccaatgcctattcacacttctacgatggctttgcacgggtgccacttgagggtgagaacaccgatgctggcgacacgttttacggtttagtgtccataaatgattttggagttttagcagttagagcagtaaaccgcagtaatccacatacaatacacacatctgtgagagtgtacatgaaaccaaaacacattcggtgttggtgccccagacctcctcgagctgtattatacaggggagagggagtggacatgatatccagtgcaattctacctctgaccaaggtagactcaattaccacttttgggtttggtcatcagaacaaagcagtgtacgttgccggttacaagatttgcaactaccacctagcaaccccaagtgatcacttgaatgcaattagtatgttatgggacagggatttaatggtggtggaatctagagcccagggaactgataccatcgccagatgtagttgcaggtgtggagtttactattgtgaatctaggaggaagtactaccctgtcacttttactggcccaacgtttcgattcatggaagcaaacgactactatccagcaagataccagtctcacatgctgatagggtgcggatttgcagaacccggggactgcggtgggatactgaggtgcactcatggggtaattggtatcattactgcaggaggtgaaggggtagtagcctttgctgacattagagacctctgggtgtatgaagaggaggccatggaacagggaataacaagctacatcgaatctctcggcacagcctttggcgcagggttcacccacacaatcagtgagaaagtgactgaattgacaacaatggttaccagcactatcacagaaaaactactgaaaaacttggtgaaaatagtgtcggctctagtgattgttgtgagaaattatgaggacactaccacgatccttgcaacactagcactactcgggtgtgatatatctccttggcaatggttgaagaagaaggcatgtgacttactagagattccttatgtgatgcgccaaggtgatgggtggatgaagaaattcacagaggcgtgcaatgcagctaaaggcttagagtggattagcaacaaaatttccaagtttatagattggttgaagtgtaaaattatcccagacgctaaggacaaggtggaatttctcaccaagttgaaacagctagacatgttggaaaatcaaattgcaaccatccaccaatcttgccccagccaagaacaacaagagattcttttcaacaatgtgagatggctagcagtccagtcccgtcggtttgcaccattatacgctgtggaggcacgccgaattaacaaaatggagagcacaataaacaattatatacagttcaagagcaaacaccgtattgaaccagtatgtatgctcattcatgggtcaccagggacgggtaaatctatagctacttcattaataggtagagcaatagcagagaaggaaagcacatcagtctattcaatgccacctgacccatctcactttgatggctataaacaacaaggggtagtgattatggacgacctaaaccaaaaccccgatggtatggacatgaaactgttttgccaaatggtatcaacagtggagtttattcctccaatggcctcattagaggagaagggcattttgtttacatctgattatgtcctggcttctaccaactctcattcaattgtaccacccacagtggctcacagtgatgccttaaccagacgatttgcatttgatgtggaggtttacacgatgtctgaacattcagtcaaaggcaaactgaatatggccacggccactcaattgtgtaaggattgtccaacacctgcaaattttaaaaagtgttgccctctcgtttgtggaaaggccttgcaattaatggacaggtacaccagacaaaggttcactgtagatgagattaccacattaatcatgaatgagaaaaacagaagggccaatatcggcaattgcatggaagccttgtttcaaggaccattaaggtataaagatttgaagatcgatgtgaagacagttcccccccctgagtgcatcagtgatttgttacaagcagtggattctcaagaggttagggattactgtgagaagaaaggctggatcgttaacgttactagccagattcaactagaaaggaacatcaatagggccatgactatactccaagctgttaccacattcgcagcagtcgcaggagtagtgtatgtaatgtacaaactcttcgccggtcaacagggtgcatacactggcttgccaaacaaaaaacccaatgtccctactatcagagtcgctaaagtccaggggccaggatttgactacgcagtggcaatggcaaaaagaaacatagttactgcaaccaccaccaagggtgaatttaccatgctaggggtgcatgataatgtagcaatattgccaacccatgccgctccaggagaaaccattattattgatgggaaagaagtagagatcctagatgccagagccttagaagatcaagcgggaaccaatcttgagatcaccattattactctaaaaagaaatgagaagtttagagacatcagatcacatattcccacccaaattactgaaactaacgatggagtgttgatcgtgaacactagcaagtaccccaatatgtatgtccccgttggtgctgtgaccgaacagggatatcttaatctcagtggacgtcaaactgctcgcactttaatgtacaactttccaacaagggcaggccagtgcggaggaatcatcacttgtactggcaaagtcattgggatgcatgttggcgggaacggttcacatgggtttgcagcagccctcaagcgatcatacttcactcaaaatcagggcgaaatccagtggatgaggtcatcaaaagaagtggggtaccccattataaatgccccatccaagacaaagttagaacccagtgctttccactatgtttttgaaggtgttaaggaaccagctgtactcactaagaatgaccccagactaaaaacagattttgaagaagccatcttttctaaatatgtggggaacaaaattactgaagtggacgagtacatgaaagaagcagtggatcactatgcaggacagttaatgtcactggatatcaacacagaacagatgtgcctggaggatgccatgtacggcaccgatggtcttgaggccctggatcttagcactagtgctggatatccttatgttgcaatggggaaaaagaaaagagacattctaaataaacagaccagagatactaaggagatgcagagacttttagatacctatggaatcaatctaccattagtcacgtacgtgaaagatgaactcaggtcaaagactaaagtggaacaaggaaagtcaagattgattgaagcttccagccttaatgattcagttgcaatgagaatggcctttggcaatctttacgcagctttccacaagaatccaggtgtggtgacaggatcagcagttggttgtgacccagatttgttttggagtaagataccagtgctaatggaagaaaaactcttcgcttttgactacacagggtatgatgcctcactcagccctgcttggtttgaagctcttaaaatggtgttagaaaaaattggatttggcagtagagtagactatatagactacctgaaccactctcaccacctttacaaaaacaagacttattgtgtcaaaggcggcatgccatccggctgctctggcacctcaattttcaactcaatgattaacaacctgatcattaggacgcttttactgagaacctacaagggcatagacttggaccatttaaaaatgattgcctatggtgatgacgtgatagcttcctacccccatgaggttgacgctagtctcctagcccaatcaggaaaagactatggactaaccatgactccagcagataaatcagtaacctttgaaacagtcacatgggagaatgtaacatttctgaaaagatttttcagagcagatgagaagtatccattcctggtgcatccagtgatgccaatgaaagaaattcacgaatcaatcagatggaccaaggaccctagaaacacacaggatcacgtacgctcgttgtgcctattagcttggcacaacggtgaagaagaatacaataaatttttagctaaaatcagaagtgtgccaattggaagagctttattgctcccagagtactctacattgtaccgccgatggctcgactcattttagtaaccctacctcagtcggattggattgggttacactgttgtaggggtaaatttttctttaattcggagaaaaaaaaaaaaaaaaaaaaaaaaagatctacctgcaggCATGCAAGCTTGGCGTAATCATGGTCATAGCTGTTTCCTGTGTGAAATTGTTATCCGCTCACAATTCCACACAACATACGAGCCGGAAGCATAAAGTGTAAAGCCTGGGGTGCCTAATGAGTGAGCTAACTCACATTAATTGCGTTGCGCTCACTGCCCGCTTTCCAGTCGGGAAACCTGTCGTGCCAGCTGCATTAATGAATCGGCCAACGCGCGGGGAGAGGCGGTTTGCGTATTGGGCGCTCTTCCGCTTCCTCGCTCACTGACTCGCTGCGCTCGGTCGTTCGGCTGCGGCGAGCGGTATCAGCTCACTCAAAGGCGGTAATACGGTTATCCACAGAATCAGGGGATAACGCAGGAAAGAACATGTGAGCAAAAGGCCAGCAAAAGGCCAGGAACCGTAAAAAGGCCGCGTTGCTGGCGTTTTTCCATAGGCTCCGCCCCCCTGACGAGCATCACAAAAATCGACGCTCAAGTCAGAGGTGGCGAAACCCGACAGGACTATAAAGATACCAGGCGTTTCCCCCTGGAAGCTCCCTCGTGCGCTCTCCTGTTCCGACCCTGCCGCTTACCGGATACCTGTCCGCCTTTCTCCCTTCGGGAAGCGTGGCGCTTTCTCATAGCTCACGCTGTAGGTATCTCAGTTCGGTGTAGGTCGTTCGCTCCAAGCTGGGCTGTGTGCACGAACCCCCCGTTCAGCCCGACCGCTGCGCCTTATCCGGTAACTATCGTCTTGAGTCCAACCCGGTAAGACACGACTTATCGCCACTGGCAGCAGCCACTGGTAACAGGATTAGCAGAGCGAGGTATGTAGGCGGTGCTACAGAGTTCTTGAAGTGGTGGCCTAACTACGGCTACACTAGAAGAACAGTATTTGGTATCTGCGCTCTGCTGAAGCCAGTTACCTTCGGAAAAAGAGTTGGTAGCTCTTGATCCGGCAAACAAACCACCGCTGGTAGCGGTGGTTTTTTTGTTTGCAAGCAGCAGATTACGCGCAGAAAAAAAGGATCTCAAGAAGATCCTTTGATCTTTTCTACGGGGTCTGACGCTCAGTGGAACGAAAACTCACGTTAAGGGATTTTGGTCATGAGATTATCAAAAAGGATCTTCACCTAGATCCTTTTAAATTAAAAATGAAGTTTTAAATCAATCTAAAGTATATATGAGTAAACTTGGTCTGACAGTTACCAATGCTTAATCAGTGAGGCACCTATCTCAGCGATCTGTCTATTTCGTTCATCCATAGTTGCCTGACTCCCCGTCGTGTAGATAACTACGATACGGGAGGGCTTACCATCTGGCCCCAGTGCTGCAATGATACCGCGAGACCCACGCTCACCGGCTCCAGATTTATCAGCAATAAACCAGCCAGCCGGAAGGGCCGAGCGCAGAAGTGGTCCTGCAACTTTATCCGCCTCCATCCAGTCTATTAATTGTTGCCGGGAAGCTAGAGTAAGTAGTTCGCCAGTTAATAGTTTGCGCAACGTTGTTGCCATTGCTACAGGCATCGTGGTGTCACGCTCGTCGTTTGGTATGGCTTCATTCAGCTCCGGTTCCCAACGATCAAGGCGAGTTACATGATCCCCCATGTTGTGCAAAAAAGCGGTTAGCTCCTTCGGTCCTCCGATCGTTGTCAGAAGTAAGTTGGCCGCAGTGTTATCACTCATGGTTATGGCAGCACTGCATAATTCTCTTACTGTCATGCCATCCGTAAGATGCTTTTCTGTGACTGGTGAGTACTCAACCAAGTCATTCTGAGAATAGTGTATGCGGCGACCGAGTTGCTCTTGCCCGGCGTCAATACGGGATAATACCGCGCCACATAGCAGAACTTTAAAAGTGCTCATCATTGGAAAACGTTCTTCGGGGCGAAAACTCTCAAGGATCTTACCGCTGTTGAGATCCAGTTCGATGTAACCCACTCGTGCACCCAACTGATCTTCAGCATCTTTTACTTTCACCAGCGTTTCTGGGTGAGCAAAAACAGGAAGGCAAAATGCCGCAAAAAAGGGAATAAGGGCGACACGGAAATGTTGAATACTCATACTCTTCCTTTTTCAATATTATTGAAGCATTTATCAGGGTTATTGTCTCATGAGCGGATACATATTTGAATGTATTTAGAAAAATAAACAAATAGGGGTTCCGCGCACATTTCCCCGAAAAGTGCCACCTGACGTCTAAGAAACCATTATTATCATGACATTAACCTATAAAAATAGGCGTATCACGAGGCCCTTTCGTCTCGCGCGTTTCGGTGATGACGGTGAAAACCTCTGACACATGCAGCTCCCGGAGACGGTCACAGCTTGTCTGTAAGCGGATGCCGGGAGCAGACAAGCCCGTCAGGGCGCGTCAGCGGGTGTTGGCGGGTGTCGGGGCTGGCTTAACTATGCGGCATCAGAGCAGATTGTACTGAGAGTGCACCATATGCGGTGTGAAATACCGCACAGATGCGTAAGGAGAAAATACCGCATCAGGCGCCATTCGCCATTCAGGCTGCGCAACTGTTGGGAAGGGCGATCGGTGCGGGCCTCTTCGCTATTACGCCAGCTGGCGAAAGGGGGATGTGCTGCAAGGCGATTAAGTTGGGTAACGCCAGGGTTTTCCCAGTCACGACGTTGTAAAACGACGGCCAGTGAATTCGAGCTCGGTACCCGGGGATCCTCTAGA

1. rCVA21-GFP-197-TC:

gtcgactaatacgactcactatagggttaaaacagctctggggttgttcccaccccagaggcccacgtggcggctagtactctggtattacggtacctttgtacgcctgttttgtatcccttcccccgtaactttagaagcttatcaaaggttcaatagcaggggtacaaaccagtacctctacgaacaagcacttctgtttccccggtgatatcacatagactgtacccacggtcaaaagtgattgatccgttatccgcttgagtacttcgagaagcctagtatcaccttggaatcttcgatgcgttgcgctcaacactctgccccgagtgtagcttaggctgatgagtctgggcactccccaccggcgacggtggcccaggctgcgttggcggcctacccatggctgatgccgtgggacgctagttgtgaacaaggtgtgaagagcctattgagctactcaagagtcctccggcccctgaatgcggctaatcctaaccacggagcaaccgctcacaacccagtgagtaggttgtcgtaatgcgtaagtctgtggcggaaccgactactttgggtgtccgtgtttccctttatattcatactggctgcttatggtgacaatttacaaattgttaccatatagctattggattggccacccagtattgtgcaatatatttgagtgtttctttcataagccttattaacatcacatttttaatcacaataaacagtgcaaatgGTGAGCAAGGGCGAGGAGCTGTTCACCGGGGTGGTGCCCATCCTGGTCGAGCTGGACGGCGACGTAAACGGCCACAAGTTCAGCGTGTCCGGCGAGGGCGAGGGCGATGCCACCTACGGCAAGCTGACCCTGAAGTTCATCTGCACCACCGGCAAGCTGCCCGTGCCCTGGCCCACCCTCGTGACCACCCTGACCTACGGCGTGCAGTGCTTCAGCCGCTACCCCGACCACATGAAGCAGCACGACTTCTTCAAGTCCGCCATGCCCGAAGGCTACGTCCAGGAGCGCACCATCTTCTTCAAGGACGACGGCAACTACAAGACCCGCGCCGAGGTGAAGTTCGAGGGCGACACCCTGGTGAACCGCATCGAGCTGAAGGGCATCGACTTCAAGGAGGACGGCAACATCCTGGGGCACAAGCTGGAGTACAACTACAACAGCCACAACGTCTATATCATGGCCGACAAGCAGAAGAACGGCATCAAGGTGAACTTCAAGATCCGCCACAACATCGAGGACGGCAGCGTGCAGCTCGCCGACCACTACCAGCAGAACACCCCCATCGGCGACGGCCCCGTGCTGCTGCCCTTCCTGAACTGCTGtCCCGGCTGtTGCATGGAGCCCGCTTTGTTTCAAGGAGCTCAAgtttcaacgcaaaagaccggtgcgcacgagaatcaaaacgtggcagccaatggatccaccattaattacactactatcaactattacaaagacagtgcgagtaattccgctactagacaagacctctcccaagatccatcaaaattcacagaaccggttaaggacttaatgttgaaaacagcaccagctctaaactcgcctaacgtggaagcatgtgggtacagtgaccgtgtgaggcaaatcactttaggcaactcgactattactacacaagaagcagccaatgctattgttgcttacggtgaatggcccacttacataaatgattcagaagctaatccggtagatgcacccactgagccagatgttagtagcaaccggttttacaccctagaatcggtgtcttggaagaccacttcaaggggatggtggtggaagttaccagattgtttgaaggacatgggaatgtttggtcagaatatgtactatcactacttggggcgctctggttacaccattcatgtccagtgcaacgcttcaaaatttcaccaaggggcgttaggagtttttctgataccagagtttgtcatggcttgcaacactgagagtaaaacgtcatacgtttcatacatcaatgcaaatcctggtgagagaggcggtgagtttacgaacacctacaatccgttaaatacagacgccagtgagggcagaaagtttgcagcattggattatttgctgggttctggtgttctagcaggaaacgcctttgtgtacccgcaccagatcatcaacctacgtaccaacaacagtgcaacaattgtggtgccatacgtaaactcacttgtgattgattgtatggcaaaacacaataactggggcattgtcatattaccactggcacccttggcctttgccgcaacatcgtcaccacaggtgcctattacagtgaccattgcacccatgtgtacagaattcaatgggttgagaaacatcaccgtcccagtacatcaagggttgccgacaatgaacacacctggttccaatcaattccttacatctgatgacttccagtcgccctgtgccttacctaattttgatgttactccaccaatacacatacccggggaagtaaagaatatgatggaactagctgaaattgacacattgatcccaatgaacgcagtggacgggaaggtgaacacaatggagatgtatcaaataccattgaatgacaatttgagcaaggcacctatattctgtttatccctatcacctgcttctgataaacgactgagccgcaccatgttgggtgaaatcctaaattattacacccattggacggggtccatcaggttcacctttctattttgtggtagtatgatggccactggtaaactgctcctcagctattccccaccgggagctaaaccaccaaccaatcgcaaggatgcaatgctaggcacacacatcatctgggacctagggttacaatccagttgttccatggttgcaccgtggatctccaacacagtgtacagacggtgtgcacgtgatgacttcactgagggcggatttataacttgcttctatcaaactagaattgtggtacctgcttcaacccctaccagtatgttcatgttaggctttgttagtgcgtgtccagacttcagtgtcagactgcttagggacactccccatattagtcaatcgaaactaataggacgtacacaaggcattgaagacctcattgacacagcgataaagaatgccttaagagtgtcccaaccaccctcgacccagtcaactgaagcaactagtggagtgaatagccaggaggtgccagctctaactgctgtggaaacaggagcatctggtcaagcaatccccagtgatgtggtggaaactaggcacgtggtaaattacaaaaccaggtctgaatcgtgtcttgagtcattctttgggagagctgcgtgtgtcacaatcctatccttgaccaactcctccaagagcggagaggagaaaaagcatttcaacatatggaatattacatacaccgacactgtccagttacgcagaaaattagagtttttcacgtattccaggtttgatcttgaaatgacttttgtattcacagagaactatcctagtacagccagtggagaagtgcgaaaccaggtgtaccagatcatgtatattccaccaggggcaccccgcccatcatcctgggatgactacacatggcaatcctcttcaaacccttccatcttctacatgtatggaaatgcacctccacggatgtcaattccttacgtagggattgccaatgcctattcacacttctacgatggctttgcacgggtgccacttgagggtgagaacaccgatgctggcgacacgttttacggtttagtgtccataaatgattttggagttttagcagttagagcagtaaaccgcagtaatccacatacaatacacacatctgtgagagtgtacatgaaaccaaaacacattcggtgttggtgccccagacctcctcgagctgtattatacaggggagagggagtggacatgatatccagtgcaattctacctctgaccaaggtagactcaattaccacttttgggtttggtcatcagaacaaagcagtgtacgttgccggttacaagatttgcaactaccacctagcaaccccaagtgatcacttgaatgcaattagtatgttatgggacagggatttaatggtggtggaatctagagcccagggaactgataccatcgccagatgtagttgcaggtgtggagtttactattgtgaatctaggaggaagtactaccctgtcacttttactggcccaacgtttcgattcatggaagcaaacgactactatccagcaagataccagtctcacatgctgatagggtgcggatttgcagaacccggggactgcggtgggatactgaggtgcactcatggggtaattggtatcattactgcaggaggtgaaggggtagtagcctttgctgacattagagacctctgggtgtatgaagaggaggccatggaacagggaataacaagctacatcgaatctctcggcacagcctttggcgcagggttcacccacacaatcagtgagaaagtgactgaattgacaacaatggttaccagcactatcacagaaaaactactgaaaaacttggtgaaaatagtgtcggctctagtgattgttgtgagaaattatgaggacactaccacgatccttgcaacactagcactactcgggtgtgatatatctccttggcaatggttgaagaagaaggcatgtgacttactagagattccttatgtgatgcgccaaggtgatgggtggatgaagaaattcacagaggcgtgcaatgcagctaaaggcttagagtggattagcaacaaaatttccaagtttatagattggttgaagtgtaaaattatcccagacgctaaggacaaggtggaatttctcaccaagttgaaacagctagacatgttggaaaatcaaattgcaaccatccaccaatcttgccccagccaagaacaacaagagattcttttcaacaatgtgagatggctagcagtccagtcccgtcggtttgcaccattatacgctgtggaggcacgccgaattaacaaaatggagagcacaataaacaattatatacagttcaagagcaaacaccgtattgaaccagtatgtatgctcattcatgggtcaccagggacgggtaaatctatagctacttcattaataggtagagcaatagcagagaaggaaagcacatcagtctattcaatgccacctgacccatctcactttgatggctataaacaacaaggggtagtgattatggacgacctaaaccaaaaccccgatggtatggacatgaaactgttttgccaaatggtatcaacagtggagtttattcctccaatggcctcattagaggagaagggcattttgtttacatctgattatgtcctggcttctaccaactctcattcaattgtaccacccacagtggctcacagtgatgccttaaccagacgatttgcatttgatgtggaggtttacacgatgtctgaacattcagtcaaaggcaaactgaatatggccacggccactcaattgtgtaaggattgtccaacacctgcaaattttaaaaagtgttgccctctcgtttgtggaaaggccttgcaattaatggacaggtacaccagacaaaggttcactgtagatgagattaccacattaatcatgaatgagaaaaacagaagggccaatatcggcaattgcatggaagccttgtttcaaggaccattaaggtataaagatttgaagatcgatgtgaagacagttcccccccctgagtgcatcagtgatttgttacaagcagtggattctcaagaggttagggattactgtgagaagaaaggctggatcgttaacgttactagccagattcaactagaaaggaacatcaatagggccatgactatactccaagctgttaccacattcgcagcagtcgcaggagtagtgtatgtaatgtacaaactcttcgccggtcaacagggtgcatacactggcttgccaaacaaaaaacccaatgtccctactatcagagtcgctaaagtccaggggccaggatttgactacgcagtggcaatggcaaaaagaaacatagttactgcaaccaccaccaagggtgaatttaccatgctaggggtgcatgataatgtagcaatattgccaacccatgccgctccaggagaaaccattattattgatgggaaagaagtagagatcctagatgccagagccttagaagatcaagcgggaaccaatcttgagatcaccattattactctaaaaagaaatgagaagtttagagacatcagatcacatattcccacccaaattactgaaactaacgatggagtgttgatcgtgaacactagcaagtaccccaatatgtatgtccccgttggtgctgtgaccgaacagggatatcttaatctcagtggacgtcaaactgctcgcactttaatgtacaactttccaacaagggcaggccagtgcggaggaatcatcacttgtactggcaaagtcattgggatgcatgttggcgggaacggttcacatgggtttgcagcagccctcaagcgatcatacttcactcaaaatcagggcgaaatccagtggatgaggtcatcaaaagaagtggggtaccccattataaatgccccatccaagacaaagttagaacccagtgctttccactatgtttttgaaggtgttaaggaaccagctgtactcactaagaatgaccccagactaaaaacagattttgaagaagccatcttttctaaatatgtggggaacaaaattactgaagtggacgagtacatgaaagaagcagtggatcactatgcaggacagttaatgtcactggatatcaacacagaacagatgtgcctggaggatgccatgtacggcaccgatggtcttgaggccctggatcttagcactagtgctggatatccttatgttgcaatggggaaaaagaaaagagacattctaaataaacagaccagagatactaaggagatgcagagacttttagatacctatggaatcaatctaccattagtcacgtacgtgaaagatgaactcaggtcaaagactaaagtggaacaaggaaagtcaagattgattgaagcttccagccttaatgattcagttgcaatgagaatggcctttggcaatctttacgcagctttccacaagaatccaggtgtggtgacaggatcagcagttggttgtgacccagatttgttttggagtaagataccagtgctaatggaagaaaaactcttcgcttttgactacacagggtatgatgcctcactcagccctgcttggtttgaagctcttaaaatggtgttagaaaaaattggatttggcagtagagtagactatatagactacctgaaccactctcaccacctttacaaaaacaagacttattgtgtcaaaggcggcatgccatccggctgctctggcacctcaattttcaactcaatgattaacaacctgatcattaggacgcttttactgagaacctacaagggcatagacttggaccatttaaaaatgattgcctatggtgatgacgtgatagcttcctacccccatgaggttgacgctagtctcctagcccaatcaggaaaagactatggactaaccatgactccagcagataaatcagtaacctttgaaacagtcacatgggagaatgtaacatttctgaaaagatttttcagagcagatgagaagtatccattcctggtgcatccagtgatgccaatgaaagaaattcacgaatcaatcagatggaccaaggaccctagaaacacacaggatcacgtacgctcgttgtgcctattagcttggcacaacggtgaagaagaatacaataaatttttagctaaaatcagaagtgtgccaattggaagagctttattgctcccagagtactctacattgtaccgccgatggctcgactcattttagtaaccctacctcagtcggattggattgggttacactgttgtaggggtaaatttttctttaattcggagaaaaaaaaaaaaaaaaaaaaaaaaagatctacctgcaggCATGCAAGCTTGGCGTAATCATGGTCATAGCTGTTTCCTGTGTGAAATTGTTATCCGCTCACAATTCCACACAACATACGAGCCGGAAGCATAAAGTGTAAAGCCTGGGGTGCCTAATGAGTGAGCTAACTCACATTAATTGCGTTGCGCTCACTGCCCGCTTTCCAGTCGGGAAACCTGTCGTGCCAGCTGCATTAATGAATCGGCCAACGCGCGGGGAGAGGCGGTTTGCGTATTGGGCGCTCTTCCGCTTCCTCGCTCACTGACTCGCTGCGCTCGGTCGTTCGGCTGCGGCGAGCGGTATCAGCTCACTCAAAGGCGGTAATACGGTTATCCACAGAATCAGGGGATAACGCAGGAAAGAACATGTGAGCAAAAGGCCAGCAAAAGGCCAGGAACCGTAAAAAGGCCGCGTTGCTGGCGTTTTTCCATAGGCTCCGCCCCCCTGACGAGCATCACAAAAATCGACGCTCAAGTCAGAGGTGGCGAAACCCGACAGGACTATAAAGATACCAGGCGTTTCCCCCTGGAAGCTCCCTCGTGCGCTCTCCTGTTCCGACCCTGCCGCTTACCGGATACCTGTCCGCCTTTCTCCCTTCGGGAAGCGTGGCGCTTTCTCATAGCTCACGCTGTAGGTATCTCAGTTCGGTGTAGGTCGTTCGCTCCAAGCTGGGCTGTGTGCACGAACCCCCCGTTCAGCCCGACCGCTGCGCCTTATCCGGTAACTATCGTCTTGAGTCCAACCCGGTAAGACACGACTTATCGCCACTGGCAGCAGCCACTGGTAACAGGATTAGCAGAGCGAGGTATGTAGGCGGTGCTACAGAGTTCTTGAAGTGGTGGCCTAACTACGGCTACACTAGAAGAACAGTATTTGGTATCTGCGCTCTGCTGAAGCCAGTTACCTTCGGAAAAAGAGTTGGTAGCTCTTGATCCGGCAAACAAACCACCGCTGGTAGCGGTGGTTTTTTTGTTTGCAAGCAGCAGATTACGCGCAGAAAAAAAGGATCTCAAGAAGATCCTTTGATCTTTTCTACGGGGTCTGACGCTCAGTGGAACGAAAACTCACGTTAAGGGATTTTGGTCATGAGATTATCAAAAAGGATCTTCACCTAGATCCTTTTAAATTAAAAATGAAGTTTTAAATCAATCTAAAGTATATATGAGTAAACTTGGTCTGACAGTTACCAATGCTTAATCAGTGAGGCACCTATCTCAGCGATCTGTCTATTTCGTTCATCCATAGTTGCCTGACTCCCCGTCGTGTAGATAACTACGATACGGGAGGGCTTACCATCTGGCCCCAGTGCTGCAATGATACCGCGAGACCCACGCTCACCGGCTCCAGATTTATCAGCAATAAACCAGCCAGCCGGAAGGGCCGAGCGCAGAAGTGGTCCTGCAACTTTATCCGCCTCCATCCAGTCTATTAATTGTTGCCGGGAAGCTAGAGTAAGTAGTTCGCCAGTTAATAGTTTGCGCAACGTTGTTGCCATTGCTACAGGCATCGTGGTGTCACGCTCGTCGTTTGGTATGGCTTCATTCAGCTCCGGTTCCCAACGATCAAGGCGAGTTACATGATCCCCCATGTTGTGCAAAAAAGCGGTTAGCTCCTTCGGTCCTCCGATCGTTGTCAGAAGTAAGTTGGCCGCAGTGTTATCACTCATGGTTATGGCAGCACTGCATAATTCTCTTACTGTCATGCCATCCGTAAGATGCTTTTCTGTGACTGGTGAGTACTCAACCAAGTCATTCTGAGAATAGTGTATGCGGCGACCGAGTTGCTCTTGCCCGGCGTCAATACGGGATAATACCGCGCCACATAGCAGAACTTTAAAAGTGCTCATCATTGGAAAACGTTCTTCGGGGCGAAAACTCTCAAGGATCTTACCGCTGTTGAGATCCAGTTCGATGTAACCCACTCGTGCACCCAACTGATCTTCAGCATCTTTTACTTTCACCAGCGTTTCTGGGTGAGCAAAAACAGGAAGGCAAAATGCCGCAAAAAAGGGAATAAGGGCGACACGGAAATGTTGAATACTCATACTCTTCCTTTTTCAATATTATTGAAGCATTTATCAGGGTTATTGTCTCATGAGCGGATACATATTTGAATGTATTTAGAAAAATAAACAAATAGGGGTTCCGCGCACATTTCCCCGAAAAGTGCCACCTGACGTCTAAGAAACCATTATTATCATGACATTAACCTATAAAAATAGGCGTATCACGAGGCCCTTTCGTCTCGCGCGTTTCGGTGATGACGGTGAAAACCTCTGACACATGCAGCTCCCGGAGACGGTCACAGCTTGTCTGTAAGCGGATGCCGGGAGCAGACAAGCCCGTCAGGGCGCGTCAGCGGGTGTTGGCGGGTGTCGGGGCTGGCTTAACTATGCGGCATCAGAGCAGATTGTACTGAGAGTGCACCATATGCGGTGTGAAATACCGCACAGATGCGTAAGGAGAAAATACCGCATCAGGCGCCATTCGCCATTCAGGCTGCGCAACTGTTGGGAAGGGCGATCGGTGCGGGCCTCTTCGCTATTACGCCAGCTGGCGAAAGGGGGATGTGCTGCAAGGCGATTAAGTTGGGTAACGCCAGGGTTTTCCCAGTCACGACGTTGTAAAACGACGGCCAGTGAATTCGAGCTCGGTACCCGGGGATCCTCTAGA

1. rCVA21-GFP-239(full length)-TC:

gtcgactaatacgactcactatagggttaaaacagctctggggttgttcccaccccagaggcccacgtggcggctagtactctggtattacggtacctttgtacgcctgttttgtatcccttcccccgtaactttagaagcttatcaaaggttcaatagcaggggtacaaaccagtacctctacgaacaagcacttctgtttccccggtgatatcacatagactgtacccacggtcaaaagtgattgatccgttatccgcttgagtacttcgagaagcctagtatcaccttggaatcttcgatgcgttgcgctcaacactctgccccgagtgtagcttaggctgatgagtctgggcactccccaccggcgacggtggcccaggctgcgttggcggcctacccatggctgatgccgtgggacgctagttgtgaacaaggtgtgaagagcctattgagctactcaagagtcctccggcccctgaatgcggctaatcctaaccacggagcaaccgctcacaacccagtgagtaggttgtcgtaatgcgtaagtctgtggcggaaccgactactttgggtgtccgtgtttccctttatattcatactggctgcttatggtgacaatttacaaattgttaccatatagctattggattggccacccagtattgtgcaatatatttgagtgtttctttcataagccttattaacatcacatttttaatcacaataaacagtgcaaatgGTGAGCAAGGGCGAGGAGCTGTTCACCGGGGTGGTGCCCATCCTGGTCGAGCTGGACGGCGACGTAAACGGCCACAAGTTCAGCGTGTCCGGCGAGGGCGAGGGCGATGCCACCTACGGCAAGCTGACCCTGAAGTTCATCTGCACCACCGGCAAGCTGCCCGTGCCCTGGCCCACCCTCGTGACCACCCTGACCTACGGCGTGCAGTGCTTCAGCCGCTACCCCGACCACATGAAGCAGCACGACTTCTTCAAGTCCGCCATGCCCGAAGGCTACGTCCAGGAGCGCACCATCTTCTTCAAGGACGACGGCAACTACAAGACCCGCGCCGAGGTGAAGTTCGAGGGCGACACCCTGGTGAACCGCATCGAGCTGAAGGGCATCGACTTCAAGGAGGACGGCAACATCCTGGGGCACAAGCTGGAGTACAACTACAACAGCCACAACGTCTATATCATGGCCGACAAGCAGAAGAACGGCATCAAGGTGAACTTCAAGATCCGCCACAACATCGAGGACGGCAGCGTGCAGCTCGCCGACCACTACCAGCAGAACACCCCCATCGGCGACGGCCCCGTGCTGCTGCCCGACAACCACTACCTGAGCACCCAGTCCGCCCTGAGCAAAGACCCCAACGAGAAGCGCGATCACATGGTCCTGCTGGAGTTCGTGACCGCCGCCGGGATCACTCTCGGCATGGACGAGCTGTACAAGTTCCTGAACTGCTGtCCCGGCTGtTGCATGGAGCCCGCTTTGTTTCAAGGAGCTCAAgtttcaacgcaaaagaccggtgcgcacgagaatcaaaacgtggcagccaatggatccaccattaattacactactatcaactattacaaagacagtgcgagtaattccgctactagacaagacctctcccaagatccatcaaaattcacagaaccggttaaggacttaatgttgaaaacagcaccagctctaaactcgcctaacgtggaagcatgtgggtacagtgaccgtgtgaggcaaatcactttaggcaactcgactattactacacaagaagcagccaatgctattgttgcttacggtgaatggcccacttacataaatgattcagaagctaatccggtagatgcacccactgagccagatgttagtagcaaccggttttacaccctagaatcggtgtcttggaagaccacttcaaggggatggtggtggaagttaccagattgtttgaaggacatgggaatgtttggtcagaatatgtactatcactacttggggcgctctggttacaccattcatgtccagtgcaacgcttcaaaatttcaccaaggggcgttaggagtttttctgataccagagtttgtcatggcttgcaacactgagagtaaaacgtcatacgtttcatacatcaatgcaaatcctggtgagagaggcggtgagtttacgaacacctacaatccgttaaatacagacgccagtgagggcagaaagtttgcagcattggattatttgctgggttctggtgttctagcaggaaacgcctttgtgtacccgcaccagatcatcaacctacgtaccaacaacagtgcaacaattgtggtgccatacgtaaactcacttgtgattgattgtatggcaaaacacaataactggggcattgtcatattaccactggcacccttggcctttgccgcaacatcgtcaccacaggtgcctattacagtgaccattgcacccatgtgtacagaattcaatgggttgagaaacatcaccgtcccagtacatcaagggttgccgacaatgaacacacctggttccaatcaattccttacatctgatgacttccagtcgccctgtgccttacctaattttgatgttactccaccaatacacatacccggggaagtaaagaatatgatggaactagctgaaattgacacattgatcccaatgaacgcagtggacgggaaggtgaacacaatggagatgtatcaaataccattgaatgacaatttgagcaaggcacctatattctgtttatccctatcacctgcttctgataaacgactgagccgcaccatgttgggtgaaatcctaaattattacacccattggacggggtccatcaggttcacctttctattttgtggtagtatgatggccactggtaaactgctcctcagctattccccaccgggagctaaaccaccaaccaatcgcaaggatgcaatgctaggcacacacatcatctgggacctagggttacaatccagttgttccatggttgcaccgtggatctccaacacagtgtacagacggtgtgcacgtgatgacttcactgagggcggatttataacttgcttctatcaaactagaattgtggtacctgcttcaacccctaccagtatgttcatgttaggctttgttagtgcgtgtccagacttcagtgtcagactgcttagggacactccccatattagtcaatcgaaactaataggacgtacacaaggcattgaagacctcattgacacagcgataaagaatgccttaagagtgtcccaaccaccctcgacccagtcaactgaagcaactagtggagtgaatagccaggaggtgccagctctaactgctgtggaaacaggagcatctggtcaagcaatccccagtgatgtggtggaaactaggcacgtggtaaattacaaaaccaggtctgaatcgtgtcttgagtcattctttgggagagctgcgtgtgtcacaatcctatccttgaccaactcctccaagagcggagaggagaaaaagcatttcaacatatggaatattacatacaccgacactgtccagttacgcagaaaattagagtttttcacgtattccaggtttgatcttgaaatgacttttgtattcacagagaactatcctagtacagccagtggagaagtgcgaaaccaggtgtaccagatcatgtatattccaccaggggcaccccgcccatcatcctgggatgactacacatggcaatcctcttcaaacccttccatcttctacatgtatggaaatgcacctccacggatgtcaattccttacgtagggattgccaatgcctattcacacttctacgatggctttgcacgggtgccacttgagggtgagaacaccgatgctggcgacacgttttacggtttagtgtccataaatgattttggagttttagcagttagagcagtaaaccgcagtaatccacatacaatacacacatctgtgagagtgtacatgaaaccaaaacacattcggtgttggtgccccagacctcctcgagctgtattatacaggggagagggagtggacatgatatccagtgcaattctacctctgaccaaggtagactcaattaccacttttgggtttggtcatcagaacaaagcagtgtacgttgccggttacaagatttgcaactaccacctagcaaccccaagtgatcacttgaatgcaattagtatgttatgggacagggatttaatggtggtggaatctagagcccagggaactgataccatcgccagatgtagttgcaggtgtggagtttactattgtgaatctaggaggaagtactaccctgtcacttttactggcccaacgtttcgattcatggaagcaaacgactactatccagcaagataccagtctcacatgctgatagggtgcggatttgcagaacccggggactgcggtgggatactgaggtgcactcatggggtaattggtatcattactgcaggaggtgaaggggtagtagcctttgctgacattagagacctctgggtgtatgaagaggaggccatggaacagggaataacaagctacatcgaatctctcggcacagcctttggcgcagggttcacccacacaatcagtgagaaagtgactgaattgacaacaatggttaccagcactatcacagaaaaactactgaaaaacttggtgaaaatagtgtcggctctagtgattgttgtgagaaattatgaggacactaccacgatccttgcaacactagcactactcgggtgtgatatatctccttggcaatggttgaagaagaaggcatgtgacttactagagattccttatgtgatgcgccaaggtgatgggtggatgaagaaattcacagaggcgtgcaatgcagctaaaggcttagagtggattagcaacaaaatttccaagtttatagattggttgaagtgtaaaattatcccagacgctaaggacaaggtggaatttctcaccaagttgaaacagctagacatgttggaaaatcaaattgcaaccatccaccaatcttgccccagccaagaacaacaagagattcttttcaacaatgtgagatggctagcagtccagtcccgtcggtttgcaccattatacgctgtggaggcacgccgaattaacaaaatggagagcacaataaacaattatatacagttcaagagcaaacaccgtattgaaccagtatgtatgctcattcatgggtcaccagggacgggtaaatctatagctacttcattaataggtagagcaatagcagagaaggaaagcacatcagtctattcaatgccacctgacccatctcactttgatggctataaacaacaaggggtagtgattatggacgacctaaaccaaaaccccgatggtatggacatgaaactgttttgccaaatggtatcaacagtggagtttattcctccaatggcctcattagaggagaagggcattttgtttacatctgattatgtcctggcttctaccaactctcattcaattgtaccacccacagtggctcacagtgatgccttaaccagacgatttgcatttgatgtggaggtttacacgatgtctgaacattcagtcaaaggcaaactgaatatggccacggccactcaattgtgtaaggattgtccaacacctgcaaattttaaaaagtgttgccctctcgtttgtggaaaggccttgcaattaatggacaggtacaccagacaaaggttcactgtagatgagattaccacattaatcatgaatgagaaaaacagaagggccaatatcggcaattgcatggaagccttgtttcaaggaccattaaggtataaagatttgaagatcgatgtgaagacagttcccccccctgagtgcatcagtgatttgttacaagcagtggattctcaagaggttagggattactgtgagaagaaaggctggatcgttaacgttactagccagattcaactagaaaggaacatcaatagggccatgactatactccaagctgttaccacattcgcagcagtcgcaggagtagtgtatgtaatgtacaaactcttcgccggtcaacagggtgcatacactggcttgccaaacaaaaaacccaatgtccctactatcagagtcgctaaagtccaggggccaggatttgactacgcagtggcaatggcaaaaagaaacatagttactgcaaccaccaccaagggtgaatttaccatgctaggggtgcatgataatgtagcaatattgccaacccatgccgctccaggagaaaccattattattgatgggaaagaagtagagatcctagatgccagagccttagaagatcaagcgggaaccaatcttgagatcaccattattactctaaaaagaaatgagaagtttagagacatcagatcacatattcccacccaaattactgaaactaacgatggagtgttgatcgtgaacactagcaagtaccccaatatgtatgtccccgttggtgctgtgaccgaacagggatatcttaatctcagtggacgtcaaactgctcgcactttaatgtacaactttccaacaagggcaggccagtgcggaggaatcatcacttgtactggcaaagtcattgggatgcatgttggcgggaacggttcacatgggtttgcagcagccctcaagcgatcatacttcactcaaaatcagggcgaaatccagtggatgaggtcatcaaaagaagtggggtaccccattataaatgccccatccaagacaaagttagaacccagtgctttccactatgtttttgaaggtgttaaggaaccagctgtactcactaagaatgaccccagactaaaaacagattttgaagaagccatcttttctaaatatgtggggaacaaaattactgaagtggacgagtacatgaaagaagcagtggatcactatgcaggacagttaatgtcactggatatcaacacagaacagatgtgcctggaggatgccatgtacggcaccgatggtcttgaggccctggatcttagcactagtgctggatatccttatgttgcaatggggaaaaagaaaagagacattctaaataaacagaccagagatactaaggagatgcagagacttttagatacctatggaatcaatctaccattagtcacgtacgtgaaagatgaactcaggtcaaagactaaagtggaacaaggaaagtcaagattgattgaagcttccagccttaatgattcagttgcaatgagaatggcctttggcaatctttacgcagctttccacaagaatccaggtgtggtgacaggatcagcagttggttgtgacccagatttgttttggagtaagataccagtgctaatggaagaaaaactcttcgcttttgactacacagggtatgatgcctcactcagccctgcttggtttgaagctcttaaaatggtgttagaaaaaattggatttggcagtagagtagactatatagactacctgaaccactctcaccacctttacaaaaacaagacttattgtgtcaaaggcggcatgccatccggctgctctggcacctcaattttcaactcaatgattaacaacctgatcattaggacgcttttactgagaacctacaagggcatagacttggaccatttaaaaatgattgcctatggtgatgacgtgatagcttcctacccccatgaggttgacgctagtctcctagcccaatcaggaaaagactatggactaaccatgactccagcagataaatcagtaacctttgaaacagtcacatgggagaatgtaacatttctgaaaagatttttcagagcagatgagaagtatccattcctggtgcatccagtgatgccaatgaaagaaattcacgaatcaatcagatggaccaaggaccctagaaacacacaggatcacgtacgctcgttgtgcctattagcttggcacaacggtgaagaagaatacaataaatttttagctaaaatcagaagtgtgccaattggaagagctttattgctcccagagtactctacattgtaccgccgatggctcgactcattttagtaaccctacctcagtcggattggattgggttacactgttgtaggggtaaatttttctttaattcggagaaaaaaaaaaaaaaaaaaaaaaaaagatctacctgcaggCATGCAAGCTTGGCGTAATCATGGTCATAGCTGTTTCCTGTGTGAAATTGTTATCCGCTCACAATTCCACACAACATACGAGCCGGAAGCATAAAGTGTAAAGCCTGGGGTGCCTAATGAGTGAGCTAACTCACATTAATTGCGTTGCGCTCACTGCCCGCTTTCCAGTCGGGAAACCTGTCGTGCCAGCTGCATTAATGAATCGGCCAACGCGCGGGGAGAGGCGGTTTGCGTATTGGGCGCTCTTCCGCTTCCTCGCTCACTGACTCGCTGCGCTCGGTCGTTCGGCTGCGGCGAGCGGTATCAGCTCACTCAAAGGCGGTAATACGGTTATCCACAGAATCAGGGGATAACGCAGGAAAGAACATGTGAGCAAAAGGCCAGCAAAAGGCCAGGAACCGTAAAAAGGCCGCGTTGCTGGCGTTTTTCCATAGGCTCCGCCCCCCTGACGAGCATCACAAAAATCGACGCTCAAGTCAGAGGTGGCGAAACCCGACAGGACTATAAAGATACCAGGCGTTTCCCCCTGGAAGCTCCCTCGTGCGCTCTCCTGTTCCGACCCTGCCGCTTACCGGATACCTGTCCGCCTTTCTCCCTTCGGGAAGCGTGGCGCTTTCTCATAGCTCACGCTGTAGGTATCTCAGTTCGGTGTAGGTCGTTCGCTCCAAGCTGGGCTGTGTGCACGAACCCCCCGTTCAGCCCGACCGCTGCGCCTTATCCGGTAACTATCGTCTTGAGTCCAACCCGGTAAGACACGACTTATCGCCACTGGCAGCAGCCACTGGTAACAGGATTAGCAGAGCGAGGTATGTAGGCGGTGCTACAGAGTTCTTGAAGTGGTGGCCTAACTACGGCTACACTAGAAGAACAGTATTTGGTATCTGCGCTCTGCTGAAGCCAGTTACCTTCGGAAAAAGAGTTGGTAGCTCTTGATCCGGCAAACAAACCACCGCTGGTAGCGGTGGTTTTTTTGTTTGCAAGCAGCAGATTACGCGCAGAAAAAAAGGATCTCAAGAAGATCCTTTGATCTTTTCTACGGGGTCTGACGCTCAGTGGAACGAAAACTCACGTTAAGGGATTTTGGTCATGAGATTATCAAAAAGGATCTTCACCTAGATCCTTTTAAATTAAAAATGAAGTTTTAAATCAATCTAAAGTATATATGAGTAAACTTGGTCTGACAGTTACCAATGCTTAATCAGTGAGGCACCTATCTCAGCGATCTGTCTATTTCGTTCATCCATAGTTGCCTGACTCCCCGTCGTGTAGATAACTACGATACGGGAGGGCTTACCATCTGGCCCCAGTGCTGCAATGATACCGCGAGACCCACGCTCACCGGCTCCAGATTTATCAGCAATAAACCAGCCAGCCGGAAGGGCCGAGCGCAGAAGTGGTCCTGCAACTTTATCCGCCTCCATCCAGTCTATTAATTGTTGCCGGGAAGCTAGAGTAAGTAGTTCGCCAGTTAATAGTTTGCGCAACGTTGTTGCCATTGCTACAGGCATCGTGGTGTCACGCTCGTCGTTTGGTATGGCTTCATTCAGCTCCGGTTCCCAACGATCAAGGCGAGTTACATGATCCCCCATGTTGTGCAAAAAAGCGGTTAGCTCCTTCGGTCCTCCGATCGTTGTCAGAAGTAAGTTGGCCGCAGTGTTATCACTCATGGTTATGGCAGCACTGCATAATTCTCTTACTGTCATGCCATCCGTAAGATGCTTTTCTGTGACTGGTGAGTACTCAACCAAGTCATTCTGAGAATAGTGTATGCGGCGACCGAGTTGCTCTTGCCCGGCGTCAATACGGGATAATACCGCGCCACATAGCAGAACTTTAAAAGTGCTCATCATTGGAAAACGTTCTTCGGGGCGAAAACTCTCAAGGATCTTACCGCTGTTGAGATCCAGTTCGATGTAACCCACTCGTGCACCCAACTGATCTTCAGCATCTTTTACTTTCACCAGCGTTTCTGGGTGAGCAAAAACAGGAAGGCAAAATGCCGCAAAAAAGGGAATAAGGGCGACACGGAAATGTTGAATACTCATACTCTTCCTTTTTCAATATTATTGAAGCATTTATCAGGGTTATTGTCTCATGAGCGGATACATATTTGAATGTATTTAGAAAAATAAACAAATAGGGGTTCCGCGCACATTTCCCCGAAAAGTGCCACCTGACGTCTAAGAAACCATTATTATCATGACATTAACCTATAAAAATAGGCGTATCACGAGGCCCTTTCGTCTCGCGCGTTTCGGTGATGACGGTGAAAACCTCTGACACATGCAGCTCCCGGAGACGGTCACAGCTTGTCTGTAAGCGGATGCCGGGAGCAGACAAGCCCGTCAGGGCGCGTCAGCGGGTGTTGGCGGGTGTCGGGGCTGGCTTAACTATGCGGCATCAGAGCAGATTGTACTGAGAGTGCACCATATGCGGTGTGAAATACCGCACAGATGCGTAAGGAGAAAATACCGCATCAGGCGCCATTCGCCATTCAGGCTGCGCAACTGTTGGGAAGGGCGATCGGTGCGGGCCTCTTCGCTATTACGCCAGCTGGCGAAAGGGGGATGTGCTGCAAGGCGATTAAGTTGGGTAACGCCAGGGTTTTCCCAGTCACGACGTTGTAAAACGACGGCCAGTGAATTCGAGCTCGGTACCCGGGGATCCTCTAGA

1. rCVA21-UnaG:

gtcgactaatacgactcactatagggttaaaacagctctggggttgttcccaccccagaggcccacgtggcggctagtactctggtattacggtacctttgtacgcctgttttgtatcccttcccccgtaactttagaagcttatcaaaggttcaatagcaggggtacaaaccagtacctctacgaacaagcacttctgtttccccggtgatatcacatagactgtacccacggtcaaaagtgattgatccgttatccgcttgagtacttcgagaagcctagtatcaccttggaatcttcgatgcgttgcgctcaacactctgccccgagtgtagcttaggctgatgagtctgggcactccccaccggcgacggtggcccaggctgcgttggcggcctacccatggctgatgccgtgggacgctagttgtgaacaaggtgtgaagagcctattgagctactcaagagtcctccggcccctgaatgcggctaatcctaaccacggagcaaccgctcacaacccagtgagtaggttgtcgtaatgcgtaagtctgtggcggaaccgactactttgggtgtccgtgtttccctttatattcatactggctgcttatggtgacaatttacaaattgttaccatatagctattggattggccacccagtattgtgcaatatatttgagtgtttctttcataagccttattaacatcacatttttaatcacaataaacagtgcaaatggtcgagaaatttgttggcacctggaagatcgcagacagccataattttggtgaatacctgaaagctatcggagccccaaaggaattaagcgatggtggggatgccacgacgccgacattgtacatctcccagaaggacggagacaaaatgacagtgaaaatagagaatggacctcctacgttccttgacactcaagtaaagttcaaattaggggaggagttcgacgaatttccttctgatcgaagaaaaggcgtaaaatctgtcgtgaacttggtgggagagaagctggtgtacgtacaaaagtgggacggcaaggagacgacgtatgtccgagagataaaggacggtaaactggtcgtgacacttacgatgggagacgtcgtggctgtgcgcagctaccggagggcgacggaaGCTTTGTTTCAAGGAGCTCAAgtttcaacgcaaaagaccggtgcgcacgagaatcaaaacgtggcagccaatggatccaccattaattacactactatcaactattacaaagacagtgcgagtaattccgctactagacaagacctctcccaagatccatcaaaattcacagaaccggttaaggacttaatgttgaaaacagcaccagctctaaactcgcctaacgtggaagcatgtgggtacagtgaccgtgtgaggcaaatcactttaggcaactcgactattactacacaagaagcagccaatgctattgttgcttacggtgaatggcccacttacataaatgattcagaagctaatccggtagatgcacccactgagccagatgttagtagcaaccggttttacaccctagaatcggtgtcttggaagaccacttcaaggggatggtggtggaagttaccagattgtttgaaggacatgggaatgtttggtcagaatatgtactatcactacttggggcgctctggttacaccattcatgtccagtgcaacgcttcaaaatttcaccaaggggcgttaggagtttttctgataccagagtttgtcatggcttgcaacactgagagtaaaacgtcatacgtttcatacatcaatgcaaatcctggtgagagaggcggtgagtttacgaacacctacaatccgttaaatacagacgccagtgagggcagaaagtttgcagcattggattatttgctgggttctggtgttctagcaggaaacgcctttgtgtacccgcaccagatcatcaacctacgtaccaacaacagtgcaacaattgtggtgccatacgtaaactcacttgtgattgattgtatggcaaaacacaataactggggcattgtcatattaccactggcacccttggcctttgccgcaacatcgtcaccacaggtgcctattacagtgaccattgcacccatgtgtacagaattcaatgggttgagaaacatcaccgtcccagtacatcaagggttgccgacaatgaacacacctggttccaatcaattccttacatctgatgacttccagtcgccctgtgccttacctaattttgatgttactccaccaatacacatacccggggaagtaaagaatatgatggaactagctgaaattgacacattgatcccaatgaacgcagtggacgggaaggtgaacacaatggagatgtatcaaataccattgaatgacaatttgagcaaggcacctatattctgtttatccctatcacctgcttctgataaacgactgagccgcaccatgttgggtgaaatcctaaattattacacccattggacggggtccatcaggttcacctttctattttgtggtagtatgatggccactggtaaactgctcctcagctattccccaccgggagctaaaccaccaaccaatcgcaaggatgcaatgctaggcacacacatcatctgggacctagggttacaatccagttgttccatggttgcaccgtggatctccaacacagtgtacagacggtgtgcacgtgatgacttcactgagggcggatttataacttgcttctatcaaactagaattgtggtacctgcttcaacccctaccagtatgttcatgttaggctttgttagtgcgtgtccagacttcagtgtcagactgcttagggacactccccatattagtcaatcgaaactaataggacgtacacaaggcattgaagacctcattgacacagcgataaagaatgccttaagagtgtcccaaccaccctcgacccagtcaactgaagcaactagtggagtgaatagccaggaggtgccagctctaactgctgtggaaacaggagcatctggtcaagcaatccccagtgatgtggtggaaactaggcacgtggtaaattacaaaaccaggtctgaatcgtgtcttgagtcattctttgggagagctgcgtgtgtcacaatcctatccttgaccaactcctccaagagcggagaggagaaaaagcatttcaacatatggaatattacatacaccgacactgtccagttacgcagaaaattagagtttttcacgtattccaggtttgatcttgaaatgacttttgtattcacagagaactatcctagtacagccagtggagaagtgcgaaaccaggtgtaccagatcatgtatattccaccaggggcaccccgcccatcatcctgggatgactacacatggcaatcctcttcaaacccttccatcttctacatgtatggaaatgcacctccacggatgtcaattccttacgtagggattgccaatgcctattcacacttctacgatggctttgcacgggtgccacttgagggtgagaacaccgatgctggcgacacgttttacggtttagtgtccataaatgattttggagttttagcagttagagcagtaaaccgcagtaatccacatacaatacacacatctgtgagagtgtacatgaaaccaaaacacattcggtgttggtgccccagacctcctcgagctgtattatacaggggagagggagtggacatgatatccagtgcaattctacctctgaccaaggtagactcaattaccacttttgggtttggtcatcagaacaaagcagtgtacgttgccggttacaagatttgcaactaccacctagcaaccccaagtgatcacttgaatgcaattagtatgttatgggacagggatttaatggtggtggaatctagagcccagggaactgataccatcgccagatgtagttgcaggtgtggagtttactattgtgaatctaggaggaagtactaccctgtcacttttactggcccaacgtttcgattcatggaagcaaacgactactatccagcaagataccagtctcacatgctgatagggtgcggatttgcagaacccggggactgcggtgggatactgaggtgcactcatggggtaattggtatcattactgcaggaggtgaaggggtagtagcctttgctgacattagagacctctgggtgtatgaagaggaggccatggaacagggaataacaagctacatcgaatctctcggcacagcctttggcgcagggttcacccacacaatcagtgagaaagtgactgaattgacaacaatggttaccagcactatcacagaaaaactactgaaaaacttggtgaaaatagtgtcggctctagtgattgttgtgagaaattatgaggacactaccacgatccttgcaacactagcactactcgggtgtgatatatctccttggcaatggttgaagaagaaggcatgtgacttactagagattccttatgtgatgcgccaaggtgatgggtggatgaagaaattcacagaggcgtgcaatgcagctaaaggcttagagtggattagcaacaaaatttccaagtttatagattggttgaagtgtaaaattatcccagacgctaaggacaaggtggaatttctcaccaagttgaaacagctagacatgttggaaaatcaaattgcaaccatccaccaatcttgccccagccaagaacaacaagagattcttttcaacaatgtgagatggctagcagtccagtcccgtcggtttgcaccattatacgctgtggaggcacgccgaattaacaaaatggagagcacaataaacaattatatacagttcaagagcaaacaccgtattgaaccagtatgtatgctcattcatgggtcaccagggacgggtaaatctatagctacttcattaataggtagagcaatagcagagaaggaaagcacatcagtctattcaatgccacctgacccatctcactttgatggctataaacaacaaggggtagtgattatggacgacctaaaccaaaaccccgatggtatggacatgaaactgttttgccaaatggtatcaacagtggagtttattcctccaatggcctcattagaggagaagggcattttgtttacatctgattatgtcctggcttctaccaactctcattcaattgtaccacccacagtggctcacagtgatgccttaaccagacgatttgcatttgatgtggaggtttacacgatgtctgaacattcagtcaaaggcaaactgaatatggccacggccactcaattgtgtaaggattgtccaacacctgcaaattttaaaaagtgttgccctctcgtttgtggaaaggccttgcaattaatggacaggtacaccagacaaaggttcactgtagatgagattaccacattaatcatgaatgagaaaaacagaagggccaatatcggcaattgcatggaagccttgtttcaaggaccattaaggtataaagatttgaagatcgatgtgaagacagttcccccccctgagtgcatcagtgatttgttacaagcagtggattctcaagaggttagggattactgtgagaagaaaggctggatcgttaacgttactagccagattcaactagaaaggaacatcaatagggccatgactatactccaagctgttaccacattcgcagcagtcgcaggagtagtgtatgtaatgtacaaactcttcgccggtcaacagggtgcatacactggcttgccaaacaaaaaacccaatgtccctactatcagagtcgctaaagtccaggggccaggatttgactacgcagtggcaatggcaaaaagaaacatagttactgcaaccaccaccaagggtgaatttaccatgctaggggtgcatgataatgtagcaatattgccaacccatgccgctccaggagaaaccattattattgatgggaaagaagtagagatcctagatgccagagccttagaagatcaagcgggaaccaatcttgagatcaccattattactctaaaaagaaatgagaagtttagagacatcagatcacatattcccacccaaattactgaaactaacgatggagtgttgatcgtgaacactagcaagtaccccaatatgtatgtccccgttggtgctgtgaccgaacagggatatcttaatctcagtggacgtcaaactgctcgcactttaatgtacaactttccaacaagggcaggccagtgcggaggaatcatcacttgtactggcaaagtcattgggatgcatgttggcgggaacggttcacatgggtttgcagcagccctcaagcgatcatacttcactcaaaatcagggcgaaatccagtggatgaggtcatcaaaagaagtggggtaccccattataaatgccccatccaagacaaagttagaacccagtgctttccactatgtttttgaaggtgttaaggaaccagctgtactcactaagaatgaccccagactaaaaacagattttgaagaagccatcttttctaaatatgtggggaacaaaattactgaagtggacgagtacatgaaagaagcagtggatcactatgcaggacagttaatgtcactggatatcaacacagaacagatgtgcctggaggatgccatgtacggcaccgatggtcttgaggccctggatcttagcactagtgctggatatccttatgttgcaatggggaaaaagaaaagagacattctaaataaacagaccagagatactaaggagatgcagagacttttagatacctatggaatcaatctaccattagtcacgtacgtgaaagatgaactcaggtcaaagactaaagtggaacaaggaaagtcaagattgattgaagcttccagccttaatgattcagttgcaatgagaatggcctttggcaatctttacgcagctttccacaagaatccaggtgtggtgacaggatcagcagttggttgtgacccagatttgttttggagtaagataccagtgctaatggaagaaaaactcttcgcttttgactacacagggtatgatgcctcactcagccctgcttggtttgaagctcttaaaatggtgttagaaaaaattggatttggcagtagagtagactatatagactacctgaaccactctcaccacctttacaaaaacaagacttattgtgtcaaaggcggcatgccatccggctgctctggcacctcaattttcaactcaatgattaacaacctgatcattaggacgcttttactgagaacctacaagggcatagacttggaccatttaaaaatgattgcctatggtgatgacgtgatagcttcctacccccatgaggttgacgctagtctcctagcccaatcaggaaaagactatggactaaccatgactccagcagataaatcagtaacctttgaaacagtcacatgggagaatgtaacatttctgaaaagatttttcagagcagatgagaagtatccattcctggtgcatccagtgatgccaatgaaagaaattcacgaatcaatcagatggaccaaggaccctagaaacacacaggatcacgtacgctcgttgtgcctattagcttggcacaacggtgaagaagaatacaataaatttttagctaaaatcagaagtgtgccaattggaagagctttattgctcccagagtactctacattgtaccgccgatggctcgactcattttagtaaccctacctcagtcggattggattgggttacactgttgtaggggtaaatttttctttaattcggagaaaaaaaaaaaaaaaaaaaaaaaaagatctacctgcaggCATGCAAGCTTGGCGTAATCATGGTCATAGCTGTTTCCTGTGTGAAATTGTTATCCGCTCACAATTCCACACAACATACGAGCCGGAAGCATAAAGTGTAAAGCCTGGGGTGCCTAATGAGTGAGCTAACTCACATTAATTGCGTTGCGCTCACTGCCCGCTTTCCAGTCGGGAAACCTGTCGTGCCAGCTGCATTAATGAATCGGCCAACGCGCGGGGAGAGGCGGTTTGCGTATTGGGCGCTCTTCCGCTTCCTCGCTCACTGACTCGCTGCGCTCGGTCGTTCGGCTGCGGCGAGCGGTATCAGCTCACTCAAAGGCGGTAATACGGTTATCCACAGAATCAGGGGATAACGCAGGAAAGAACATGTGAGCAAAAGGCCAGCAAAAGGCCAGGAACCGTAAAAAGGCCGCGTTGCTGGCGTTTTTCCATAGGCTCCGCCCCCCTGACGAGCATCACAAAAATCGACGCTCAAGTCAGAGGTGGCGAAACCCGACAGGACTATAAAGATACCAGGCGTTTCCCCCTGGAAGCTCCCTCGTGCGCTCTCCTGTTCCGACCCTGCCGCTTACCGGATACCTGTCCGCCTTTCTCCCTTCGGGAAGCGTGGCGCTTTCTCATAGCTCACGCTGTAGGTATCTCAGTTCGGTGTAGGTCGTTCGCTCCAAGCTGGGCTGTGTGCACGAACCCCCCGTTCAGCCCGACCGCTGCGCCTTATCCGGTAACTATCGTCTTGAGTCCAACCCGGTAAGACACGACTTATCGCCACTGGCAGCAGCCACTGGTAACAGGATTAGCAGAGCGAGGTATGTAGGCGGTGCTACAGAGTTCTTGAAGTGGTGGCCTAACTACGGCTACACTAGAAGAACAGTATTTGGTATCTGCGCTCTGCTGAAGCCAGTTACCTTCGGAAAAAGAGTTGGTAGCTCTTGATCCGGCAAACAAACCACCGCTGGTAGCGGTGGTTTTTTTGTTTGCAAGCAGCAGATTACGCGCAGAAAAAAAGGATCTCAAGAAGATCCTTTGATCTTTTCTACGGGGTCTGACGCTCAGTGGAACGAAAACTCACGTTAAGGGATTTTGGTCATGAGATTATCAAAAAGGATCTTCACCTAGATCCTTTTAAATTAAAAATGAAGTTTTAAATCAATCTAAAGTATATATGAGTAAACTTGGTCTGACAGTTACCAATGCTTAATCAGTGAGGCACCTATCTCAGCGATCTGTCTATTTCGTTCATCCATAGTTGCCTGACTCCCCGTCGTGTAGATAACTACGATACGGGAGGGCTTACCATCTGGCCCCAGTGCTGCAATGATACCGCGAGACCCACGCTCACCGGCTCCAGATTTATCAGCAATAAACCAGCCAGCCGGAAGGGCCGAGCGCAGAAGTGGTCCTGCAACTTTATCCGCCTCCATCCAGTCTATTAATTGTTGCCGGGAAGCTAGAGTAAGTAGTTCGCCAGTTAATAGTTTGCGCAACGTTGTTGCCATTGCTACAGGCATCGTGGTGTCACGCTCGTCGTTTGGTATGGCTTCATTCAGCTCCGGTTCCCAACGATCAAGGCGAGTTACATGATCCCCCATGTTGTGCAAAAAAGCGGTTAGCTCCTTCGGTCCTCCGATCGTTGTCAGAAGTAAGTTGGCCGCAGTGTTATCACTCATGGTTATGGCAGCACTGCATAATTCTCTTACTGTCATGCCATCCGTAAGATGCTTTTCTGTGACTGGTGAGTACTCAACCAAGTCATTCTGAGAATAGTGTATGCGGCGACCGAGTTGCTCTTGCCCGGCGTCAATACGGGATAATACCGCGCCACATAGCAGAACTTTAAAAGTGCTCATCATTGGAAAACGTTCTTCGGGGCGAAAACTCTCAAGGATCTTACCGCTGTTGAGATCCAGTTCGATGTAACCCACTCGTGCACCCAACTGATCTTCAGCATCTTTTACTTTCACCAGCGTTTCTGGGTGAGCAAAAACAGGAAGGCAAAATGCCGCAAAAAAGGGAATAAGGGCGACACGGAAATGTTGAATACTCATACTCTTCCTTTTTCAATATTATTGAAGCATTTATCAGGGTTATTGTCTCATGAGCGGATACATATTTGAATGTATTTAGAAAAATAAACAAATAGGGGTTCCGCGCACATTTCCCCGAAAAGTGCCACCTGACGTCTAAGAAACCATTATTATCATGACATTAACCTATAAAAATAGGCGTATCACGAGGCCCTTTCGTCTCGCGCGTTTCGGTGATGACGGTGAAAACCTCTGACACATGCAGCTCCCGGAGACGGTCACAGCTTGTCTGTAAGCGGATGCCGGGAGCAGACAAGCCCGTCAGGGCGCGTCAGCGGGTGTTGGCGGGTGTCGGGGCTGGCTTAACTATGCGGCATCAGAGCAGATTGTACTGAGAGTGCACCATATGCGGTGTGAAATACCGCACAGATGCGTAAGGAGAAAATACCGCATCAGGCGCCATTCGCCATTCAGGCTGCGCAACTGTTGGGAAGGGCGATCGGTGCGGGCCTCTTCGCTATTACGCCAGCTGGCGAAAGGGGGATGTGCTGCAAGGCGATTAAGTTGGGTAACGCCAGGGTTTTCCCAGTCACGACGTTGTAAAACGACGGCCAGTGAATTCGAGCTCGGTACCCGGGGATCCTCTAGA

1. miniCMV-CVA21:

gtcgacggagcactgtcctccgaacgtcggagcactgtcctccgaacgtcggagcactgtcctccgaacgtcggagcactgtcctccgaacgtcGGAGCACTGTCCTCCGAACGcgaggtaggcgtgtacggtgggCgcctataAaagcagagctcgtttagtgaaccgtcagatcgcctggagttaaaacagctctggggttgttcccaccccagaggcccacgtggcggctagtactctggtattacggtacctttgtacgcctgttttgtatcccttcccccgtaactttagaagcttatcaaaggttcaatagcaggggtacaaaccagtacctctacgaacaagcacttctgtttccccggtgatatcacatagactgtacccacggtcaaaagtgattgatccgttatccgcttgagtacttcgagaagcctagtatcaccttggaatcttcgatgcgttgcgctcaacactctgccccgagtgtagcttaggctgatgagtctgggcactccccaccggcgacggtggcccaggctgcgttggcggcctacccatggctgatgccgtgggacgctagttgtgaacaaggtgtgaagagcctattgagctactcaagagtcctccggcccctgaatgcggctaatcctaaccacggagcaaccgctcacaacccagtgagtaggttgtcgtaatgcgtaagtctgtggcggaaccgactactttgggtgtccgtgtttccctttatattcatactggctgcttatggtgacaatttacaaattgttaccatatagctattggattggccacccagtattgtgcaatatatttgagtgtttctttcataagccttattaacatcacatttttaatcacaataaacagtgcaaatgggggctcaagtttcaacgcaaaagaccggtgcgcacgagaatcaaaacgtggcagccaatggatccaccattaattacactactatcaactattacaaagacagtgcgagtaattccgctactagacaagacctctcccaagatccatcaaaattcacagaaccggttaaggacttaatgttgaaaacagcaccagctctaaactcgcctaacgtggaagcatgtgggtacagtgaccgtgtgaggcaaatcactttaggcaactcgactattactacacaagaagcagccaatgctattgttgcttacggtgaatggcccacttacataaatgattcagaagctaatccggtagatgcacccactgagccagatgttagtagcaaccggttttacaccctagaatcggtgtcttggaagaccacttcaaggggatggtggtggaagttaccagattgtttgaaggacatgggaatgtttggtcagaatatgtactatcactacttggggcgctctggttacaccattcatgtccagtgcaacgcttcaaaatttcaccaaggggcgttaggagtttttctgataccagagtttgtcatggcttgcaacactgagagtaaaacgtcatacgtttcatacatcaatgcaaatcctggtgagagaggcggtgagtttacgaacacctacaatccgttaaatacagacgccagtgagggcagaaagtttgcagcattggattatttgctgggttctggtgttctagcaggaaacgcctttgtgtacccgcaccagatcatcaacctacgtaccaacaacagtgcaacaattgtggtgccatacgtaaactcacttgtgattgattgtatggcaaaacacaataactggggcattgtcatattaccactggcacccttggcctttgccgcaacatcgtcaccacaggtgcctattacagtgaccattgcacccatgtgtacagaattcaatgggttgagaaacatcaccgtcccagtacatcaagggttgccgacaatgaacacacctggttccaatcaattccttacatctgatgacttccagtcgccctgtgccttacctaattttgatgttactccaccaatacacatacccggggaagtaaagaatatgatggaactagctgaaattgacacattgatcccaatgaacgcagtggacgggaaggtgaacacaatggagatgtatcaaataccattgaatgacaatttgagcaaggcacctatattctgtttatccctatcacctgcttctgataaacgactgagccgcaccatgttgggtgaaatcctaaattattacacccattggacggggtccatcaggttcacctttctattttgtggtagtatgatggccactggtaaactgctcctcagctattccccaccgggagctaaaccaccaaccaatcgcaaggatgcaatgctaggcacacacatcatctgggacctagggttacaatccagttgttccatggttgcaccgtggatctccaacacagtgtacagacggtgtgcacgtgatgacttcactgagggcggatttataacttgcttctatcaaactagaattgtggtacctgcttcaacccctaccagtatgttcatgttaggctttgttagtgcgtgtccagacttcagtgtcagactgcttagggacactccccatattagtcaatcgaaactaataggacgtacacaaggcattgaagacctcattgacacagcgataaagaatgccttaagagtgtcccaaccaccctcgacccagtcaactgaagcaactagtggagtgaatagccaggaggtgccagctctaactgctgtggaaacaggagcatctggtcaagcaatccccagtgatgtggtggaaactaggcacgtggtaaattacaaaaccaggtctgaatcgtgtcttgagtcattctttgggagagctgcgtgtgtcacaatcctatccttgaccaactcctccaagagcggagaggagaaaaagcatttcaacatatggaatattacatacaccgacactgtccagttacgcagaaaattagagtttttcacgtattccaggtttgatcttgaaatgacttttgtattcacagagaactatcctagtacagccagtggagaagtgcgaaaccaggtgtaccagatcatgtatattccaccaggggcaccccgcccatcatcctgggatgactacacatggcaatcctcttcaaacccttccatcttctacatgtatggaaatgcacctccacggatgtcaattccttacgtagggattgccaatgcctattcacacttctacgatggctttgcacgggtgccacttgagggtgagaacaccgatgctggcgacacgttttacggtttagtgtccataaatgattttggagttttagcagttagagcagtaaaccgcagtaatccacatacaatacacacatctgtgagagtgtacatgaaaccaaaacacattcggtgttggtgccccagacctcctcgagctgtattatacaggggagagggagtggacatgatatccagtgcaattctacctctgaccaaggtagactcaattaccacttttgggtttggtcatcagaacaaagcagtgtacgttgccggttacaagatttgcaactaccacctagcaaccccaagtgatcacttgaatgcaattagtatgttatgggacagggatttaatggtggtggaatctagagcccagggaactgataccatcgccagatgtagttgcaggtgtggagtttactattgtgaatctaggaggaagtactaccctgtcacttttactggcccaacgtttcgattcatggaagcaaacgactactatccagcaagataccagtctcacatgctgatagggtgcggatttgcagaacccggggactgcggtgggatactgaggtgcactcatggggtaattggtatcattactgcaggaggtgaaggggtagtagcctttgctgacattagagacctctgggtgtatgaagaggaggccatggaacagggaataacaagctacatcgaatctctcggcacagcctttggcgcagggttcacccacacaatcagtgagaaagtgactgaattgacaacaatggttaccagcactatcacagaaaaactactgaaaaacttggtgaaaatagtgtcggctctagtgattgttgtgagaaattatgaggacactaccacgatccttgcaacactagcactactcgggtgtgatatatctccttggcaatggttgaagaagaaggcatgtgacttactagagattccttatgtgatgcgccaaggtgatgggtggatgaagaaattcacagaggcgtgcaatgcagctaaaggcttagagtggattagcaacaaaatttccaagtttatagattggttgaagtgtaaaattatcccagacgctaaggacaaggtggaatttctcaccaagttgaaacagctagacatgttggaaaatcaaattgcaaccatccaccaatcttgccccagccaagaacaacaagagattcttttcaacaatgtgagatggctagcagtccagtcccgtcggtttgcaccattatacgctgtggaggcacgccgaattaacaaaatggagagcacaataaacaattatatacagttcaagagcaaacaccgtattgaaccagtatgtatgctcattcatgggtcaccagggacgggtaaatctatagctacttcattaataggtagagcaatagcagagaaggaaagcacatcagtctattcaatgccacctgacccatctcactttgatggctataaacaacaaggggtagtgattatggacgacctaaaccaaaaccccgatggtatggacatgaaactgttttgccaaatggtatcaacagtggagtttattcctccaatggcctcattagaggagaagggcattttgtttacatctgattatgtcctggcttctaccaactctcattcaattgtaccacccacagtggctcacagtgatgccttaaccagacgatttgcatttgatgtggaggtttacacgatgtctgaacattcagtcaaaggcaaactgaatatggccacggccactcaattgtgtaaggattgtccaacacctgcaaattttaaaaagtgttgccctctcgtttgtggaaaggccttgcaattaatggacaggtacaccagacaaaggttcactgtagatgagattaccacattaatcatgaatgagaaaaacagaagggccaatatcggcaattgcatggaagccttgtttcaaggaccattaaggtataaagatttgaagatcgatgtgaagacagttcccccccctgagtgcatcagtgatttgttacaagcagtggattctcaagaggttagggattactgtgagaagaaaggctggatcgttaacgttactagccagattcaactagaaaggaacatcaatagggccatgactatactccaagctgttaccacattcgcagcagtcgcaggagtagtgtatgtaatgtacaaactcttcgccggtcaacagggtgcatacactggcttgccaaacaaaaaGcccaatgtccctactatcagagtcgctaaagtccaggggccaggatttgactacgcagtggcaatggcaaaaagaaacatagttactgcaaccaccaccaagggtgaatttaccatgctaggggtgcatgataatgtagcaatattgccaacccatgccgctccaggagaaaccattattattgatgggaaagaagtagagatcctagatgccagagccttagaagatcaagcgggaaccaatcttgagatcaccattattactctaaaaagaaatgagaagtttagagacatcagatcacatattcccacccaaattactgaaactaacgatggagtgttgatcgtgaacactagcaagtaccccaatatgtatgtccccgttggtgctgtgaccgaacagggatatcttaatctcagtggacgtcaaactgctcgcactttaatgtacaactttccaacaagggcaggccagtgcggaggaatcatcacttgtactggcaaagtcattgggatgcatgttggcgggaacggttcacatgggtttgcagcagccctcaagcgatcatacttcactcaaaatcagggcgaaatccagtggatgaggtcatcaaaagaagtggggtaccccattataaatgccccatccaagacaaagttagaacccagtgctttccactatgtttttgaaggtgttaaggaaccagctgtactcactaagaatgaccccagactaaaaacagattttgaagaagccatcttttctaaatatgtggggaacaaaattactgaagtggacgagtacatgaaagaagcagtggatcactatgcaggacagttaatgtcactggatatcaacacagaacagatgtgcctggaggatgccatgtacggcaccgatggtcttgaggccctggatcttagcactagtgctggatatccttatgttgcaatggggaaaaagaaaagagacattctaaataaacagaccagagatactaaggagatgcagagacttttagatacctatggaatcaatctaccattagtcacgtacgtgaaagatgaactcaggtcaaagactaaagtggaacaaggaaagtcaagattgattgaagcttccagccttaatgattcagttgcaatgagaatggcctttggcaatctttacgcagctttccacaagaatccaggtgtggtgacaggatcagcagttggttgtgacccagatttgttttggagtaagataccagtgctaatggaagaaaaactcttcgcttttgactacacagggtatgatgcctcactcagccctgcttggtttgaagctcttaaaatggtgttagaaaaaattggatttggcagtagagtagactatatagactacctgaaccactctcaccacctttacaaaaacaagacttattgtgtcaaaggcggcatgccatccggctgctctggcacctcaattttcaactcaatgattaacaacctgatcattaggacgcttttactgagaacctacaagggcatagacttggaccatttaaaaatgattgcctatggtgatgacgtgatagcttcctacccccatgaggttgacgctagtctcctagcccaatcaggaaaagactatggactaaccatgactccagcagataaatcagtaacctttgaaacagtcacatgggagaatgtaacatttctgaaaagatttttcagagcagatgagaagtatccattcctggtgcatccagtgatgccaatgaaagaaattcacgaatcaatcagatggaccaaggaccctagaaacacacaggatcacgtacgctcgttgtgcctattagcttggcacaacggtgaagaagaatacaataaatttttagctaaaatcagaagtgtgccaattggaagagctttattgctcccagagtactctacattgtaccgccgatggctcgactcattttagtaaccctacctcagtcggattggattgggttacactgttgtaggggtaaatttttctttaattcggagaaaaaaaaaaaaaaaaaaaaaaaaagatctAGACATGATAAGATACATTGATGAGTTTGGACAAACCACAACAAGAATGCAGTGAAAAAAATGCTTTATTTGTGAAATTTGTGATGCTATTGCTTTATTTGTAACCATTATAAGCTGCAATAAACAAGTTAACAACAACAATTGCATTCATTTTATGTTTCAGGTTCAGGGGGAGATGTGGGAGGTTTTTTAAAGCAAGTAAAACCTCTACAAATGTGGTAccgcgggcctcgcgatctgcgcagcaccatggcctgaaataacctctgaaagaggaacttggttagctaccttctgaggcggaaagaaccagctgtggaatgtgtgtcagttagggtgtggaaagtccccaggctccccagcaggcagaagtatgcaaagcatgcatctcaattagtcagcaaccaggtgtggaaagtccccaggctccccagcaggcagaagtatgcaaagcatgcatctcaattagtcagcaaccatagtcccgcccctaactccgcccatcccgcccctaactccgcccagttccgcccattctccgccccatggctgactaattttttttatttatgcagaggccgaggccgcctctgcctctgagctattccagaagtagtgaggaggcttttttggaggcctaggcttttgcaaaaagctcgattcttctgacactagcgccaccatgaccgagtacaagcctaccgtgcgcctggccactcgcgatgatgtgccccgcgccgtccgcactctggccgccgctttcgccgactaccccgctacccggcacaccgtggaccccgaccggcacatcgagcgtgtgacagagttgcaggagctgttcctgacccgcgtcgggctggacatcggcaaggtgtgggtagccgacgacggcgcggccgtggccgtgtggactacccccgagagcgttgaggccggcgccgtgttcgccgagatcggcccccgaatggccgagctgagcggcagccgcctggccgcccagcagcaaatggagggcctgcttgccccccatcgtcccaaggagcctgcctggtttctggccactgtaggagtgagccccgaccaccagggcaagggcttgggcagcgccgtcgtgttgcccggcgtagaggccgccgaacgcgccggtgtgcccgcctttctcgaaacaagcgcaccaagaaaccttccattctacgagcgcctgggcttcaccgtgaccgccgatgtcgaggtgcccgagggacctaggacctggtgtatgacacgaaaacctggcgcctaatgatctagaaccggtcatggccgcaataaaatatctttattttcattacatctgtgtgttggttttttgtgtgttcgaactagatgctcctgcaggCATGCAAGCTTGGCGTAATCATGGTCATAGCTGTTTCCTGTGTGAAATTGTTATCCGCTCACAATTCCACACAACATACGAGCCGGAAGCATAAAGTGTAAAGCCTGGGGTGCCTAATGAGTGAGCTAACTCACATTAATTGCGTTGCGCTCACTGCCCGCTTTCCAGTCGGGAAACCTGTCGTGCCAGCTGCATTAATGAATCGGCCAACGCGCGGGGAGAGGCGGTTTGCGTATTGGGCGCTCTTCCGCTTCCTCGCTCACTGACTCGCTGCGCTCGGTCGTTCGGCTGCGGCGAGCGGTATCAGCTCACTCAAAGGCGGTAATACGGTTATCCACAGAATCAGGGGATAACGCAGGAAAGAACATGTGAGCAAAAGGCCAGCAAAAGGCCAGGAACCGTAAAAAGGCCGCGTTGCTGGCGTTTTTCCATAGGCTCCGCCCCCCTGACGAGCATCACAAAAATCGACGCTCAAGTCAGAGGTGGCGAAACCCGACAGGACTATAAAGATACCAGGCGTTTCCCCCTGGAAGCTCCCTCGTGCGCTCTCCTGTTCCGACCCTGCCGCTTACCGGATACCTGTCCGCCTTTCTCCCTTCGGGAAGCGTGGCGCTTTCTCATAGCTCACGCTGTAGGTATCTCAGTTCGGTGTAGGTCGTTCGCTCCAAGCTGGGCTGTGTGCACGAACCCCCCGTTCAGCCCGACCGCTGCGCCTTATCCGGTAACTATCGTCTTGAGTCCAACCCGGTAAGACACGACTTATCGCCACTGGCAGCAGCCACTGGTAACAGGATTAGCAGAGCGAGGTATGTAGGCGGTGCTACAGAGTTCTTGAAGTGGTGGCCTAACTACGGCTACACTAGAAGAACAGTATTTGGTATCTGCGCTCTGCTGAAGCCAGTTACCTTCGGAAAAAGAGTTGGTAGCTCTTGATCCGGCAAACAAACCACCGCTGGTAGCGGTGGTTTTTTTGTTTGCAAGCAGCAGATTACGCGCAGAAAAAAAGGATCTCAAGAAGATCCTTTGATCTTTTCTACGGGGTCTGACGCTCAGTGGAACGAAAACTCACGTTAAGGGATTTTGGTCATGAGATTATCAAAAAGGATCTTCACCTAGATCCTTTTAAATTAAAAATGAAGTTTTAAATCAATCTAAAGTATATATGAGTAAACTTGGTCTGACAGTTACCAATGCTTAATCAGTGAGGCACCTATCTCAGCGATCTGTCTATTTCGTTCATCCATAGTTGCCTGACTCCCCGTCGTGTAGATAACTACGATACGGGAGGGCTTACCATCTGGCCCCAGTGCTGCAATGATACCGCGAGACCCACGCTCACCGGCTCCAGATTTATCAGCAATAAACCAGCCAGCCGGAAGGGCCGAGCGCAGAAGTGGTCCTGCAACTTTATCCGCCTCCATCCAGTCTATTAATTGTTGCCGGGAAGCTAGAGTAAGTAGTTCGCCAGTTAATAGTTTGCGCAACGTTGTTGCCATTGCTACAGGCATCGTGGTGTCACGCTCGTCGTTTGGTATGGCTTCATTCAGCTCCGGTTCCCAACGATCAAGGCGAGTTACATGATCCCCCATGTTGTGCAAAAAAGCGGTTAGCTCCTTCGGTCCTCCGATCGTTGTCAGAAGTAAGTTGGCCGCAGTGTTATCACTCATGGTTATGGCAGCACTGCATAATTCTCTTACTGTCATGCCATCCGTAAGATGCTTTTCTGTGACTGGTGAGTACTCAACCAAGTCATTCTGAGAATAGTGTATGCGGCGACCGAGTTGCTCTTGCCCGGCGTCAATACGGGATAATACCGCGCCACATAGCAGAACTTTAAAAGTGCTCATCATTGGAAAACGTTCTTCGGGGCGAAAACTCTCAAGGATCTTACCGCTGTTGAGATCCAGTTCGATGTAACCCACTCGTGCACCCAACTGATCTTCAGCATCTTTTACTTTCACCAGCGTTTCTGGGTGAGCAAAAACAGGAAGGCAAAATGCCGCAAAAAAGGGAATAAGGGCGACACGGAAATGTTGAATACTCATACTCTTCCTTTTTCAATATTATTGAAGCATTTATCAGGGTTATTGTCTCATGAGCGGATACATATTTGAATGTATTTAGAAAAATAAACAAATAGGGGTTCCGCGCACATTTCCCCGAAAAGTGCCACCTGACGTCTAAGAAACCATTATTATCATGACATTAACCTATAAAAATAGGCGTATCACGAGGCCCTTTCGTCTCGCGCGTTTCGGTGATGACGGTGAAAACCTCTGACACATGCAGCTCCCGGAGACGGTCACAGCTTGTCTGTAAGCGGATGCCGGGAGCAGACAAGCCCGTCAGGGCGCGTCAGCGGGTGTTGGCGGGTGTCGGGGCTGGCTTAACTATGCGGCATCAGAGCAGATTGTACTGAGAGTGCACCATATGCGGTGTGAAATACCGCACAGATGCGTAAGGAGAAAATACCGCATCAGGCGCCATTCGCCATTCAGGCTGCGCAACTGTTGGGAAGGGCGATCGGTGCGGGCCTCTTCGCTATTACGCCAGCTGGCGAAAGGGGGATGTGCTGCAAGGCGATTAAGTTGGGTAACGCCAGGGTTTTCCCAGTCACGACGTTGTAAAACGACGGCCAGTGAATTCGAGCTCGGTACCCGGGGATCCTCTAGA

1. CMV-CVA21:

gtcgacTAGTTATTAATAGTAATCAATTACGGGGTCATTAGTTCATAGCCCATATATGGAGTTCCGCGTTACATAACTTACGGTAAATGGCCCGCCTGGCTGACCGCCCAACGACCCCCGCCCATTGACGTCAATAATGACGTATGTTCCCATAGTAACGCCAATAGGGACTTTCCATTGACGTCAATGGGTGGAGTATTTACGGTAAACTGCCCACTTGGCAGTACATCAAGTGTATCATATGCCAAGTACGCCCCCTATTGACGTCAATGACGGTAAATGGCCCGCCTGGCATTATGCCCAGTACATGACCTTATGGGACTTTCCTACTTGGCAGTACATCTACGTATTAGTCATCGCTATTACCATGGTGATGCGGTTTTGGCAGTACATCAATGGGCGTGGATAGCGGTTTGACTCACGGGGATTTCCAAGTCTCCACCCCATTGACGTCAATGGGAGTTTGTTTTGGCACCAAAATCAACGGGACTTTCCAAAATGTCGTAACAACTCCGCCCCATTGACGCAAATGGGCGGTAGGCGTGTACGGTGGGAGGTCTATATAAGCAGAGCTGGTTTAGTGAACCGTCAGATCgcctggagttaaaacagctctggggttgttcccaccccagaggcccacgtggcggctagtactctggtattacggtacctttgtacgcctgttttgtatcccttcccccgtaactttagaagcttatcaaaggttcaatagcaggggtacaaaccagtacctctacgaacaagcacttctgtttccccggtgatatcacatagactgtacccacggtcaaaagtgattgatccgttatccgcttgagtacttcgagaagcctagtatcaccttggaatcttcgatgcgttgcgctcaacactctgccccgagtgtagcttaggctgatgagtctgggcactccccaccggcgacggtggcccaggctgcgttggcggcctacccatggctgatgccgtgggacgctagttgtgaacaaggtgtgaagagcctattgagctactcaagagtcctccggcccctgaatgcggctaatcctaaccacggagcaaccgctcacaacccagtgagtaggttgtcgtaatgcgtaagtctgtggcggaaccgactactttgggtgtccgtgtttccctttatattcatactggctgcttatggtgacaatttacaaattgttaccatatagctattggattggccacccagtattgtgcaatatatttgagtgtttctttcataagccttattaacatcacatttttaatcacaataaacagtgcaaatgggggctcaagtttcaacgcaaaagaccggtgcgcacgagaatcaaaacgtggcagccaatggatccaccattaattacactactatcaactattacaaagacagtgcgagtaattccgctactagacaagacctctcccaagatccatcaaaattcacagaaccggttaaggacttaatgttgaaaacagcaccagctctaaactcgcctaacgtggaagcatgtgggtacagtgaccgtgtgaggcaaatcactttaggcaactcgactattactacacaagaagcagccaatgctattgttgcttacggtgaatggcccacttacataaatgattcagaagctaatccggtagatgcacccactgagccagatgttagtagcaaccggttttacaccctagaatcggtgtcttggaagaccacttcaaggggatggtggtggaagttaccagattgtttgaaggacatgggaatgtttggtcagaatatgtactatcactacttggggcgctctggttacaccattcatgtccagtgcaacgcttcaaaatttcaccaaggggcgttaggagtttttctgataccagagtttgtcatggcttgcaacactgagagtaaaacgtcatacgtttcatacatcaatgcaaatcctggtgagagaggcggtgagtttacgaacacctacaatccgttaaatacagacgccagtgagggcagaaagtttgcagcattggattatttgctgggttctggtgttctagcaggaaacgcctttgtgtacccgcaccagatcatcaacctacgtaccaacaacagtgcaacaattgtggtgccatacgtaaactcacttgtgattgattgtatggcaaaacacaataactggggcattgtcatattaccactggcacccttggcctttgccgcaacatcgtcaccacaggtgcctattacagtgaccattgcacccatgtgtacagaattcaatgggttgagaaacatcaccgtcccagtacatcaagggttgccgacaatgaacacacctggttccaatcaattccttacatctgatgacttccagtcgccctgtgccttacctaattttgatgttactccaccaatacacatacccggggaagtaaagaatatgatggaactagctgaaattgacacattgatcccaatgaacgcagtggacgggaaggtgaacacaatggagatgtatcaaataccattgaatgacaatttgagcaaggcacctatattctgtttatccctatcacctgcttctgataaacgactgagccgcaccatgttgggtgaaatcctaaattattacacccattggacggggtccatcaggttcacctttctattttgtggtagtatgatggccactggtaaactgctcctcagctattccccaccgggagctaaaccaccaaccaatcgcaaggatgcaatgctaggcacacacatcatctgggacctagggttacaatccagttgttccatggttgcaccgtggatctccaacacagtgtacagacggtgtgcacgtgatgacttcactgagggcggatttataacttgcttctatcaaactagaattgtggtacctgcttcaacccctaccagtatgttcatgttaggctttgttagtgcgtgtccagacttcagtgtcagactgcttagggacactccccatattagtcaatcgaaactaataggacgtacacaaggcattgaagacctcattgacacagcgataaagaatgccttaagagtgtcccaaccaccctcgacccagtcaactgaagcaactagtggagtgaatagccaggaggtgccagctctaactgctgtggaaacaggagcatctggtcaagcaatccccagtgatgtggtggaaactaggcacgtggtaaattacaaaaccaggtctgaatcgtgtcttgagtcattctttgggagagctgcgtgtgtcacaatcctatccttgaccaactcctccaagagcggagaggagaaaaagcatttcaacatatggaatattacatacaccgacactgtccagttacgcagaaaattagagtttttcacgtattccaggtttgatcttgaaatgacttttgtattcacagagaactatcctagtacagccagtggagaagtgcgaaaccaggtgtaccagatcatgtatattccaccaggggcaccccgcccatcatcctgggatgactacacatggcaatcctcttcaaacccttccatcttctacatgtatggaaatgcacctccacggatgtcaattccttacgtagggattgccaatgcctattcacacttctacgatggctttgcacgggtgccacttgagggtgagaacaccgatgctggcgacacgttttacggtttagtgtccataaatgattttggagttttagcagttagagcagtaaaccgcagtaatccacatacaatacacacatctgtgagagtgtacatgaaaccaaaacacattcggtgttggtgccccagacctcctcgagctgtattatacaggggagagggagtggacatgatatccagtgcaattctacctctgaccaaggtagactcaattaccacttttgggtttggtcatcagaacaaagcagtgtacgttgccggttacaagatttgcaactaccacctagcaaccccaagtgatcacttgaatgcaattagtatgttatgggacagggatttaatggtggtggaatctagagcccagggaactgataccatcgccagatgtagttgcaggtgtggagtttactattgtgaatctaggaggaagtactaccctgtcacttttactggcccaacgtttcgattcatggaagcaaacgactactatccagcaagataccagtctcacatgctgatagggtgcggatttgcagaacccggggactgcggtgggatactgaggtgcactcatggggtaattggtatcattactgcaggaggtgaaggggtagtagcctttgctgacattagagacctctgggtgtatgaagaggaggccatggaacagggaataacaagctacatcgaatctctcggcacagcctttggcgcagggttcacccacacaatcagtgagaaagtgactgaattgacaacaatggttaccagcactatcacagaaaaactactgaaaaacttggtgaaaatagtgtcggctctagtgattgttgtgagaaattatgaggacactaccacgatccttgcaacactagcactactcgggtgtgatatatctccttggcaatggttgaagaagaaggcatgtgacttactagagattccttatgtgatgcgccaaggtgatgggtggatgaagaaattcacagaggcgtgcaatgcagctaaaggcttagagtggattagcaacaaaatttccaagtttatagattggttgaagtgtaaaattatcccagacgctaaggacaaggtggaatttctcaccaagttgaaacagctagacatgttggaaaatcaaattgcaaccatccaccaatcttgccccagccaagaacaacaagagattcttttcaacaatgtgagatggctagcagtccagtcccgtcggtttgcaccattatacgctgtggaggcacgccgaattaacaaaatggagagcacaataaacaattatatacagttcaagagcaaacaccgtattgaaccagtatgtatgctcattcatgggtcaccagggacgggtaaatctatagctacttcattaataggtagagcaatagcagagaaggaaagcacatcagtctattcaatgccacctgacccatctcactttgatggctataaacaacaaggggtagtgattatggacgacctaaaccaaaaccccgatggtatggacatgaaactgttttgccaaatggtatcaacagtggagtttattcctccaatggcctcattagaggagaagggcattttgtttacatctgattatgtcctggcttctaccaactctcattcaattgtaccacccacagtggctcacagtgatgccttaaccagacgatttgcatttgatgtggaggtttacacgatgtctgaacattcagtcaaaggcaaactgaatatggccacggccactcaattgtgtaaggattgtccaacacctgcaaattttaaaaagtgttgccctctcgtttgtggaaaggccttgcaattaatggacaggtacaccagacaaaggttcactgtagatgagattaccacattaatcatgaatgagaaaaacagaagggccaatatcggcaattgcatggaagccttgtttcaaggaccattaaggtataaagatttgaagatcgatgtgaagacagttcccccccctgagtgcatcagtgatttgttacaagcagtggattctcaagaggttagggattactgtgagaagaaaggctggatcgttaacgttactagccagattcaactagaaaggaacatcaatagggccatgactatactccaagctgttaccacattcgcagcagtcgcaggagtagtgtatgtaatgtacaaactcttcgccggtcaacagggtgcatacactggcttgccaaacaaaaaGcccaatgtccctactatcagagtcgctaaagtccaggggccaggatttgactacgcagtggcaatggcaaaaagaaacatagttactgcaaccaccaccaagggtgaatttaccatgctaggggtgcatgataatgtagcaatattgccaacccatgccgctccaggagaaaccattattattgatgggaaagaagtagagatcctagatgccagagccttagaagatcaagcgggaaccaatcttgagatcaccattattactctaaaaagaaatgagaagtttagagacatcagatcacatattcccacccaaattactgaaactaacgatggagtgttgatcgtgaacactagcaagtaccccaatatgtatgtccccgttggtgctgtgaccgaacagggatatcttaatctcagtggacgtcaaactgctcgcactttaatgtacaactttccaacaagggcaggccagtgcggaggaatcatcacttgtactggcaaagtcattgggatgcatgttggcgggaacggttcacatgggtttgcagcagccctcaagcgatcatacttcactcaaaatcagggcgaaatccagtggatgaggtcatcaaaagaagtggggtaccccattataaatgccccatccaagacaaagttagaacccagtgctttccactatgtttttgaaggtgttaaggaaccagctgtactcactaagaatgaccccagactaaaaacagattttgaagaagccatcttttctaaatatgtggggaacaaaattactgaagtggacgagtacatgaaagaagcagtggatcactatgcaggacagttaatgtcactggatatcaacacagaacagatgtgcctggaggatgccatgtacggcaccgatggtcttgaggccctggatcttagcactagtgctggatatccttatgttgcaatggggaaaaagaaaagagacattctaaataaacagaccagagatactaaggagatgcagagacttttagatacctatggaatcaatctaccattagtcacgtacgtgaaagatgaactcaggtcaaagactaaagtggaacaaggaaagtcaagattgattgaagcttccagccttaatgattcagttgcaatgagaatggcctttggcaatctttacgcagctttccacaagaatccaggtgtggtgacaggatcagcagttggttgtgacccagatttgttttggagtaagataccagtgctaatggaagaaaaactcttcgcttttgactacacagggtatgatgcctcactcagccctgcttggtttgaagctcttaaaatggtgttagaaaaaattggatttggcagtagagtagactatatagactacctgaaccactctcaccacctttacaaaaacaagacttattgtgtcaaaggcggcatgccatccggctgctctggcacctcaattttcaactcaatgattaacaacctgatcattaggacgcttttactgagaacctacaagggcatagacttggaccatttaaaaatgattgcctatggtgatgacgtgatagcttcctacccccatgaggttgacgctagtctcctagcccaatcaggaaaagactatggactaaccatgactccagcagataaatcagtaacctttgaaacagtcacatgggagaatgtaacatttctgaaaagatttttcagagcagatgagaagtatccattcctggtgcatccagtgatgccaatgaaagaaattcacgaatcaatcagatggaccaaggaccctagaaacacacaggatcacgtacgctcgttgtgcctattagcttggcacaacggtgaagaagaatacaataaatttttagctaaaatcagaagtgtgccaattggaagagctttattgctcccagagtactctacattgtaccgccgatggctcgactcattttagtaaccctacctcagtcggattggattgggttacactgttgtaggggtaaatttttctttaattcggagaaaaaaaaaaaaaaaaaaaaaaaaagatctAGACATGATAAGATACATTGATGAGTTTGGACAAACCACAACAAGAATGCAGTGAAAAAAATGCTTTATTTGTGAAATTTGTGATGCTATTGCTTTATTTGTAACCATTATAAGCTGCAATAAACAAGTTAACAACAACAATTGCATTCATTTTATGTTTCAGGTTCAGGGGGAGATGTGGGAGGTTTTTTAAAGCAAGTAAAACCTCTACAAATGTGGTAccgcgggcctcgcgatctgcgcagcaccatggcctgaaataacctctgaaagaggaacttggttagctaccttctgaggcggaaagaaccagctgtggaatgtgtgtcagttagggtgtggaaagtccccaggctccccagcaggcagaagtatgcaaagcatgcatctcaattagtcagcaaccaggtgtggaaagtccccaggctccccagcaggcagaagtatgcaaagcatgcatctcaattagtcagcaaccatagtcccgcccctaactccgcccatcccgcccctaactccgcccagttccgcccattctccgccccatggctgactaattttttttatttatgcagaggccgaggccgcctctgcctctgagctattccagaagtagtgaggaggcttttttggaggcctaggcttttgcaaaaagctcgattcttctgacactagcgccaccatgaccgagtacaagcctaccgtgcgcctggccactcgcgatgatgtgccccgcgccgtccgcactctggccgccgctttcgccgactaccccgctacccggcacaccgtggaccccgaccggcacatcgagcgtgtgacagagttgcaggagctgttcctgacccgcgtcgggctggacatcggcaaggtgtgggtagccgacgacggcgcggccgtggccgtgtggactacccccgagagcgttgaggccggcgccgtgttcgccgagatcggcccccgaatggccgagctgagcggcagccgcctggccgcccagcagcaaatggagggcctgcttgccccccatcgtcccaaggagcctgcctggtttctggccactgtaggagtgagccccgaccaccagggcaagggcttgggcagcgccgtcgtgttgcccggcgtagaggccgccgaacgcgccggtgtgcccgcctttctcgaaacaagcgcaccaagaaaccttccattctacgagcgcctgggcttcaccgtgaccgccgatgtcgaggtgcccgagggacctaggacctggtgtatgacacgaaaacctggcgcctaatgatctagaaccggtcatggccgcaataaaatatctttattttcattacatctgtgtgttggttttttgtgtgttcgaactagatgctcctgcaggCATGCAAGCTTGGCGTAATCATGGTCATAGCTGTTTCCTGTGTGAAATTGTTATCCGCTCACAATTCCACACAACATACGAGCCGGAAGCATAAAGTGTAAAGCCTGGGGTGCCTAATGAGTGAGCTAACTCACATTAATTGCGTTGCGCTCACTGCCCGCTTTCCAGTCGGGAAACCTGTCGTGCCAGCTGCATTAATGAATCGGCCAACGCGCGGGGAGAGGCGGTTTGCGTATTGGGCGCTCTTCCGCTTCCTCGCTCACTGACTCGCTGCGCTCGGTCGTTCGGCTGCGGCGAGCGGTATCAGCTCACTCAAAGGCGGTAATACGGTTATCCACAGAATCAGGGGATAACGCAGGAAAGAACATGTGAGCAAAAGGCCAGCAAAAGGCCAGGAACCGTAAAAAGGCCGCGTTGCTGGCGTTTTTCCATAGGCTCCGCCCCCCTGACGAGCATCACAAAAATCGACGCTCAAGTCAGAGGTGGCGAAACCCGACAGGACTATAAAGATACCAGGCGTTTCCCCCTGGAAGCTCCCTCGTGCGCTCTCCTGTTCCGACCCTGCCGCTTACCGGATACCTGTCCGCCTTTCTCCCTTCGGGAAGCGTGGCGCTTTCTCATAGCTCACGCTGTAGGTATCTCAGTTCGGTGTAGGTCGTTCGCTCCAAGCTGGGCTGTGTGCACGAACCCCCCGTTCAGCCCGACCGCTGCGCCTTATCCGGTAACTATCGTCTTGAGTCCAACCCGGTAAGACACGACTTATCGCCACTGGCAGCAGCCACTGGTAACAGGATTAGCAGAGCGAGGTATGTAGGCGGTGCTACAGAGTTCTTGAAGTGGTGGCCTAACTACGGCTACACTAGAAGAACAGTATTTGGTATCTGCGCTCTGCTGAAGCCAGTTACCTTCGGAAAAAGAGTTGGTAGCTCTTGATCCGGCAAACAAACCACCGCTGGTAGCGGTGGTTTTTTTGTTTGCAAGCAGCAGATTACGCGCAGAAAAAAAGGATCTCAAGAAGATCCTTTGATCTTTTCTACGGGGTCTGACGCTCAGTGGAACGAAAACTCACGTTAAGGGATTTTGGTCATGAGATTATCAAAAAGGATCTTCACCTAGATCCTTTTAAATTAAAAATGAAGTTTTAAATCAATCTAAAGTATATATGAGTAAACTTGGTCTGACAGTTACCAATGCTTAATCAGTGAGGCACCTATCTCAGCGATCTGTCTATTTCGTTCATCCATAGTTGCCTGACTCCCCGTCGTGTAGATAACTACGATACGGGAGGGCTTACCATCTGGCCCCAGTGCTGCAATGATACCGCGAGACCCACGCTCACCGGCTCCAGATTTATCAGCAATAAACCAGCCAGCCGGAAGGGCCGAGCGCAGAAGTGGTCCTGCAACTTTATCCGCCTCCATCCAGTCTATTAATTGTTGCCGGGAAGCTAGAGTAAGTAGTTCGCCAGTTAATAGTTTGCGCAACGTTGTTGCCATTGCTACAGGCATCGTGGTGTCACGCTCGTCGTTTGGTATGGCTTCATTCAGCTCCGGTTCCCAACGATCAAGGCGAGTTACATGATCCCCCATGTTGTGCAAAAAAGCGGTTAGCTCCTTCGGTCCTCCGATCGTTGTCAGAAGTAAGTTGGCCGCAGTGTTATCACTCATGGTTATGGCAGCACTGCATAATTCTCTTACTGTCATGCCATCCGTAAGATGCTTTTCTGTGACTGGTGAGTACTCAACCAAGTCATTCTGAGAATAGTGTATGCGGCGACCGAGTTGCTCTTGCCCGGCGTCAATACGGGATAATACCGCGCCACATAGCAGAACTTTAAAAGTGCTCATCATTGGAAAACGTTCTTCGGGGCGAAAACTCTCAAGGATCTTACCGCTGTTGAGATCCAGTTCGATGTAACCCACTCGTGCACCCAACTGATCTTCAGCATCTTTTACTTTCACCAGCGTTTCTGGGTGAGCAAAAACAGGAAGGCAAAATGCCGCAAAAAAGGGAATAAGGGCGACACGGAAATGTTGAATACTCATACTCTTCCTTTTTCAATATTATTGAAGCATTTATCAGGGTTATTGTCTCATGAGCGGATACATATTTGAATGTATTTAGAAAAATAAACAAATAGGGGTTCCGCGCACATTTCCCCGAAAAGTGCCACCTGACGTCTAAGAAACCATTATTATCATGACATTAACCTATAAAAATAGGCGTATCACGAGGCCCTTTCGTCTCGCGCGTTTCGGTGATGACGGTGAAAACCTCTGACACATGCAGCTCCCGGAGACGGTCACAGCTTGTCTGTAAGCGGATGCCGGGAGCAGACAAGCCCGTCAGGGCGCGTCAGCGGGTGTTGGCGGGTGTCGGGGCTGGCTTAACTATGCGGCATCAGAGCAGATTGTACTGAGAGTGCACCATATGCGGTGTGAAATACCGCACAGATGCGTAAGGAGAAAATACCGCATCAGGCGCCATTCGCCATTCAGGCTGCGCAACTGTTGGGAAGGGCGATCGGTGCGGGCCTCTTCGCTATTACGCCAGCTGGCGAAAGGGGGATGTGCTGCAAGGCGATTAAGTTGGGTAACGCCAGGGTTTTCCCAGTCACGACGTTGTAAAACGACGGCCAGTGAATTCGAGCTCGGTACCCGGGGATCCTCTAGA
